# Eco-friendly and efficient catalyst-free synthesis of *N*-sulfonylimines from sulfonamides and aldehydes: crucial role of Al_2_O_3_ as a reusable dehydrating agent†

**DOI:** 10.1039/d2ra08304c

**Published:** 2023-02-02

**Authors:** Zaineb Litim, Hanen Slimi, Thierry Ollevier, Jamil Kraïem

**Affiliations:** a Laboratoire de Développement Chimique, Galénique et Pharmacologique des Médicaments, Faculté de Pharmacie de Monastir, Université de Monastir Rue Avicenne 5000 Monastir Tunisia jamil.kraiem@fphm.u-monastir.tn; b Département de Chimie, Université Laval 1045 Avenue de la Médecine Québec Québec G1V 0A6 Canada thierry.ollevier@chm.ulaval.ca

## Abstract

A green synthetic method for the synthesis of *N*-sulfonylimines was developed involving the straightforward condensation of sulfonamides with aldehydes under green and catalyst-free conditions, mediated by neutral Al_2_O_3_ as an efficient and reusable dehydrating agent. *N*-Sulfonylimines were produced in high yields and purity under simple experimental procedures.

## Abstract 2

A green synthesis of *N*-sulfonylimines was developed involving the straightforward condensation of sulfonamides with aldehydes under green and catalyst-free conditions, mediated by neutral Al_2_O_3_ as an efficient and reusable dehydrating agent.

## Graphical abstract

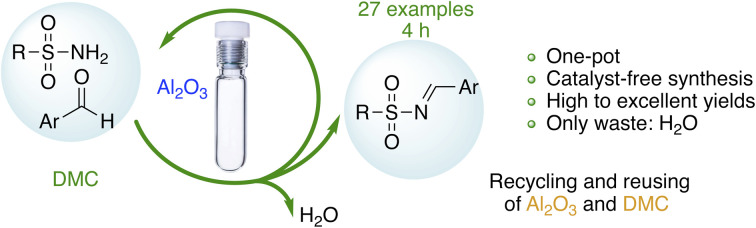

Imines containing electron-withdrawing *N*-substituents are well-recognized intermediates toward molecules with synthetic and biological interest.^1^ Especially, *N*-sulfonylimines are the most widely studied derivatives due to their unique characteristics that are not easily found in other electron-deficient imines. *N*-Sulfonylimines demonstrate a good compromise between being sufficiently stable imines but also reactive enough to be versatile substrates in many transformations as well. In addition, they have been extensively explored as excellent activated electrophiles in hetero-Diels–Alder reactions,^2^ nucleophilic additions to afford chiral amines,^3^ imino-aldol reactions,^4^ reductions,^5^ aza-Friedel–Crafts,^6^ ene-reactions,^7^ as well as in the asymmetric synthesis of β-amino acid derivatives.^8^ They have also been applied as important precursors in the preparation of a variety of heterocyclic compounds such as oxaziridines,^9^ imidazolines,^10^ and aziridines.^11^

So far, with the increasing demand for *N*-sulfonylimines, numerous methods to prepare these compounds have been reported. Most of them involve the direct condensation of sulfonamides with aldehydes and are mediated by: (i) strong Lewis acid catalysts, including FeCl_3_,^12^ WCl_6_,^13^ ZnCl_2_/SiO_2_,^14^ TiCl_4_/Et_3_N,^15^ and AlCl_3_,^16^ (ii) Brønsted acid catalysts, such as Amberlyst 15/molecular sieves,^17^ HY9 zeolite,^18^ and sulfamic acid (NH_2_SO_3_H),^19^ (iii) organocatalysts, such as pyrrolidine/molecular sieves,^20^ and Ar_3_CCl,^21^ and (iv) activating reagents, *i.e.* Si(OEt)_4_,^22^ TFAA,^23^ Silphox [POCl_3−*n*_(SiO_2_)_*n*_],^24^ and silica chloride (SiO_2_–Cl).^25^ Some methods are multi-step procedures, such as the reaction of aldehydes with isocyanates analogues,^26^ oxidation of *N*-sulfinimines,^27^ rearrangement of oxime *O*-sulfinates,^28^ and condensation of benzyl alcohols with chloramine-T or sulfonamides.^29^ It should be mentioned that most of these methods have one or more of the following disadvantages: harsh reaction conditions, unsatisfactory yields, use of hazardous reagents and toxic solvents, expensive and non-recyclable catalysts, generation of toxic waste, and tedious work-up.

The ideal synthetic route for the *N*-sulfonylimines formation is the direct condensation of sulfonamides with aldehydes. However, it is worth mentioning that the conversion rate may be limited due to the unfavorable thermodynamics because of water generated in the reaction medium as a by-product (Scheme 1), and by the weak nucleophilicity of sulfonamides, which usually required elevated temperatures and strong acidic conditions to achieve carbonyl activation. Nevertheless, these conditions are generally incompatible with acid-sensitive substrates and the limited stability of the resulting imines in the medium.

**Scheme 1:**
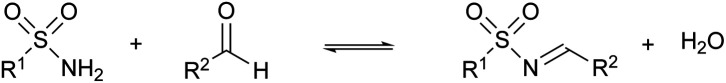
One-pot synthesis of *N*-sulfonylaldimines from sulfonamides and aldehydes.

From a green chemistry perspective, catalyst-free procedures have many advantages, due to the simple experimental procedure, low cost, and importantly the compatibility for acid or base sensitive substrates. To our knowledge, there is no efficient catalyst-free procedure for the synthesis of *N*-sulfonylimines from aldehydes and sulfonamides described in the literature. Poisson *et al.* have reported the condensation of *p*-tosylamide with benzaldehyde under catalyst-free conditions, and the conversion was reported not to exceed 75% under microwave irradiation, even when heating up to 180 °C.^30^ Therefore, these authors replaced this reaction by the condensation of sulfonamides with benzaldehyde dimethylacetal instead of benzaldehyde to achieve full conversions into the sulfonylimines.

Considering these precedents and in the context of our interest in the design and development of a green synthetic methodology,^9,31^ we think that the development of a new straightforward strategy for the *N*-sulfonylimine synthesis under mild and catalyst-free conditions would be highly desirable. For this reason, we carried out a systematic study in which we examined the influence of the solvent, temperature, and dehydrating agent on the condensation of sulfonamides with aldehydes. This study aims at finding the optimal conditions for an efficient synthesis of the corresponding *N*-sulfonylimines under green conditions. In this work, we used a pressure tube as an efficient and environmentally benign heating technique which received considerable attention as a preferable and practical alternative in many organic transformations. This technique meets with green chemistry protocols by affording a cleaner reaction profile in a shorter reaction time, energy saving, preventing the use of high temperature, excess of solvents, and release of toxic gas.^20,31,32^

Herein, we disclose a successful demonstration of the catalyst-free direct condensation of *N*-sulfonamides with aldehydes mediated by neutral Al_2_O_3_, as an efficient and reusable dehydrating agent, under simple and eco-friendly conditions. To investigate the optimum conditions for the direct synthesis of *N*-sulfonylimines under catalyst-free conditions, we chose *p*-tosylamide 1a and *p*-anisaldehyde 2a as the model substrates. The results of the optimization study are summarized in Table 1. We first examined the reaction at 110 °C (Table 1, entries 1–3) to evaluate the ability of sulfonamide 1a and aldehyde 2a to undergo condensation without any additive. We noticed that 58% of the corresponding imine 3a was formed under solvent-free conditions after 12 h of reaction time; 38% of 3a was formed after 4 h when anhydrous DMC (dimethyl carbonate) was used as the solvent of the reaction. In line with these promising results, it appeared that the reaction worked equally well in the absence of a catalyst (Table 1, entry 2). The incomplete conversion was unsurprisingly attained due to the water generation in the reaction media, leading to the hydrolysis of 3a and decreased the yield of the imine. This problem motivated us to study the effect of water scavenger additives on the *N*-sulfonylimine production as an alternative process for the removal of water produced during the reaction. Various common activated neutral and basic dehydrating agents such as 4 Å molecular sieves, neutral Al_2_O_3_, MgO, and Na_2_CO_3_ have been investigated to test their performance (Table 1, entries 6 and 8–10). Lower temperature (Table 1, entry 7) led to lower conversion (no conversion was observed at rt). The best results were obtained when neutral Al_2_O_3_ was used as the dehydrating agent at 110 °C and in the presence of anhydrous DMC as the solvent of the reaction (Table 1, entry 6). In this case, a total conversion of 1a and 2a into the imine 3a was observed after 4 h. The influence of the solvent on the reaction efficiency was also studied (Table 1, entries 6 and 11–15) which led to total conversion of 1a and 2a into the corresponding imine 3a in 4 h when DMC was used as the solvent, and after 6 h under solvent-free conditions.

**Table 1:** Optimization of the reaction conditions^a^

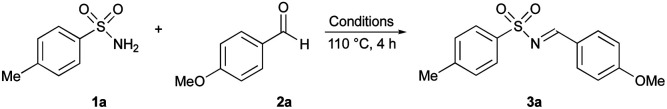			
Entry	Additive	Solvent	Conv.^b^ (%)
1	—	—	45
2	—	—	58^c^
3	—	DMC	38
4	Al_2_O_3_	—	97
5	Al_2_O_3_	—	100^d^
6	Al_2_O_3_	DMC	**100**
7	Al_2_O_3_	DMC	80^f^
8	MgO	DMC	50
9	MS (4 Å)	DMC	16
10	Na_2_CO_3_	DMC	31
11	Al_2_O_3_	PC^e^	96
12	Al_2_O_3_	AcOEt	47
13	Al_2_O_3_	EtOH	0
14	Al_2_O_3_	Toluene	61
15	Al_2_O_3_	CH_2_Cl_2_	39

a Reaction conditions: *p*-tosylamide 1a (1.2 mmol), *p*-anisaldehyde 2a (1 mmol), additive (2 equiv.), solvent (1 mL). The mixture was stirred in a pressure tube.

b Determined by ^1^H NMR analysis of the crude product.

c Conversion after 12 h.

d Full conversion (100%) after 6 h.

e Propylene carbonate.

f Reaction performed at 90 °C.

In this study, we emphasize that dialkyl carbonates, such as DMC and PC (propylene carbonate), have been found to be far better solvents than the other ones (Table 1, entries 6 and 8). In fact, DMC and PC are highly recommended as green and sustainable alternative solvents for chemical transformations, owing to their low toxicity, non-corrosive, high biodegradability, economical manufacturing, and the use of abundant and renewable sources such as CO_2_ in their production.^9,31,33^

Thus, the optimized reaction conditions obtained for the synthesis of *N*-sulfonylimine 3a were achieved by stirring a mixture of *N*-tosylamide 1a, aldehyde 2a, and neutral Al_2_O_3_ in dry DMC for 4 h at 110 °C. Pure product was obtained by filtration of the insoluble Al_2_O_3_ and purifying the crude by simple recrystallization. In order to demonstrate the feasibility, as well as the generality of the method, a wide variety of aryl aldehydes and sulfonamides were investigated under the optimized conditions (Table 2), and the corresponding *N*-sulfonylimines 3a–yc were successfully obtained in good to excellent yields. It is worth noting that the presence of electron-donating substituents, as well as electron-withdrawing substituents, on the aromatic ring of aldehydes had no significant influence on the efficiency of the process. Unsurprisingly, all *N*-sulfonylimines 3a–yc were obtained exclusively in the *E*-isomer form. Indeed, the less stable *Z*-isomer is thermodynamically unfavoured. These stereochemical results are in line with the ones obtained in other procedures described in the literature.^12–29^ Interestingly, when a carboxylic amide was used instead of a sulfonyl amide, the corresponding acyl imine was not formed.^34^

**Table 2:** Preparation of *N*-sulfonylimines 3a–yc from sulfonamides 1 and aldehydes 2^a^

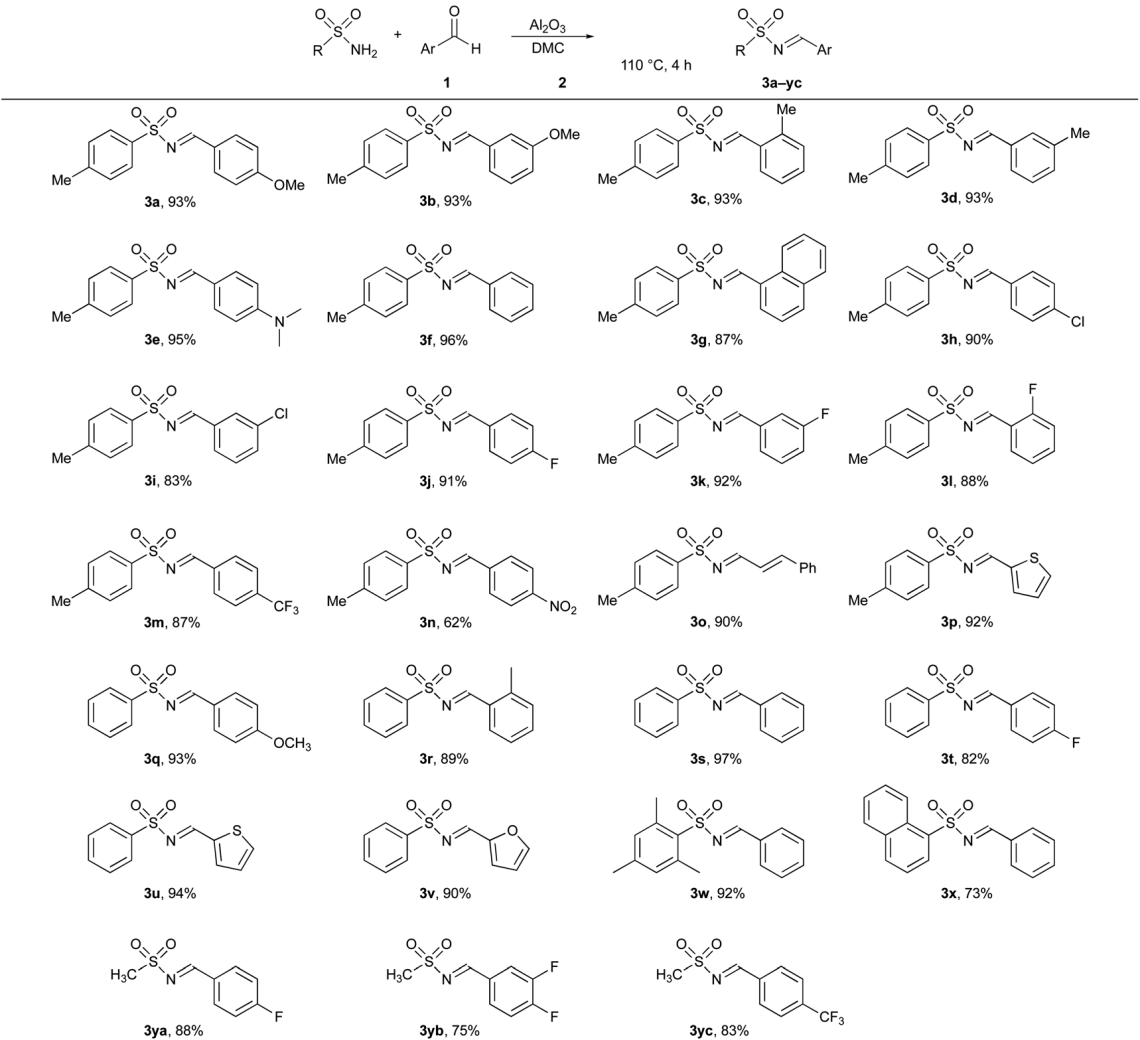

a Reaction conditions: *N*-sulfonamide (1.2 mmol), aldehyde (1 mmol), Al_2_O_3_ (2 mmol), DMC (1 mL), the reaction was heated at 110 °C in a pressure tube for 4 h (reaction monitored by TLC).

The catalyst-free approach for the synthesis of *N*-sulfonylimines was successfully demonstrated. In fact, it is worth pointing out the crucial role of the dehydration played by aluminium oxide in the described reaction. Al_2_O_3_ is well known for its high adsorption capacity when exposed to moist air or wet solution and has proved to be one of the most effective adsorbents applied for removing traces or bulk of water from fluids (gas, liquid).^35^ In addition, it is characterized by its wide surface area, high stability, and mechanical strength, also, it can be highlighted as inexpensive, reusable, and commercially available. The unique physical and chemical proprieties as well as the non-toxic and environmentally nature of Al_2_O_3_ make it a promising material for the application in the laboratory and in diverse areas of industry.^36^ Furthermore, from a practical point of view, this inorganic compound provides a significant solution to known synthetic drawbacks while using *N*-sulfonylimines. Interestingly, Al_2_O_3_ (i) is able to capture a large quantity of water from the reaction medium, consequently pushing the equilibrium toward the formation of *N*-sulfonylimines without the need of addition of a catalyst; (ii) does not react with the product, whereas conventional routes commonly involve harsh acidic conditions which make them unsuitable for acid-sensitive substrates, and (iii) can be easily separated from the reaction medium and be reused after simple washing with DMC. Indeed, it was found that the use of Al_2_O_3_ in the preparation of imine 3f afforded the same efficiency in the 2^nd^, 3^rd^, 4^th^, and 5^th^ runs (100% conversions, 92–96% yields) as in the first run.

A comparison of the efficiency of our method with some previously reported methods for the preparation of *N*-sulfonylimine 3f was highlighted in Table 3. The only catalyst-free approach described for the direct synthesis of *N*-sulfonylimines showed a conversion of 75% when molecular sieves were used as dehydrating agent.^30^ However, the present method furnished higher yields and similar efficiency as the reported methodologies mediated by catalysts. Noteworthily, the combination of an inexpensive dehydrating agent with the use of green and recyclable solvent (DMC) without the use of a catalyst makes this strategy a suitable alternative for the synthesis of *N*-sulfonylimines.

**Table 3:** The comparative synthesis of the *N*-sulfonylimine 3f using the present work *vs.* previous methods

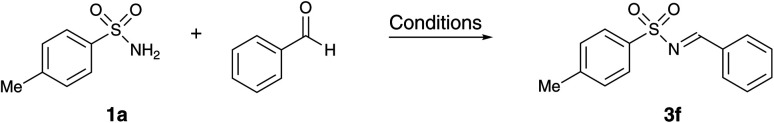			
Conditions	Additive	Catalyst	Yield 3f (%)
**DMC, 110 °C, 4 h**	**Al**_**2**_ **O**_**3**_	**—**	**96**
MW, 180 °C, 0.5 h	3 Å + 4 Å MS	—	75 (conv.)^30^
CH_2_Cl_2_, 0 °C, 0.5 h	—	TiCl_4_, Et_3_N	58 (ref. 15)
CH_2_Cl_2_, 60 °C, 24 h	4 Å MS	Pyrrolidine (10%)	99 (ref. 20)
PhMe, 110 °C, 16 h	5 Å MS	Amberlyst 15	90 (ref. 36)
EtOH, rt, 1 h	—	FeCl_3_ (4%)	92 (ref. 12)
160 °C, 6 h	—	Si(OEt)_4_	68 (ref. 22)

To sum up, we have developed herein an efficient catalyst-free one-pot synthesis of *N*-sulfonylaldimines by condensation of *N*-sulfonamides with aldehydes, in the presence of neutral Al_2_O_3_ as the dehydrating agent and DMC as a green and recyclable solvent. Al_2_O_3_ was found to be a highly effective, commercially available, inexpensive, and heterogeneous dehydrating additive. This new strategy appears to provide one of the most practical and environmentally benign routes to *N*-sulfonylimines. The method uses catalyst-free conditions, simple experimental procedures, and green chemistry protocols to afford *N*-sulfonylaldimines in high yield and low cost.

## Conflicts of interest

There are no conflicts to declare.

## Supplementary Material

RA-013-D2RA08304C-s001
