# Supplementary material for: Eco-friendly and efficient catalyst-free synthesis of N-sulfonylimines from sulfonamides and aldehydes: crucial role of Al2O3 as a reusable dehydrating agent
Source: RSC Adv. 2023 Feb 2;13(7):4431–5. doi: 10.1039/d2ra08304c (PMC9892885; doi:10.1039/d2ra08304c)
Supplement: RA-013-D2RA08304C-s001 [file RA-013-D2RA08304C-s001.pdf]

Electronic Supplementary Material (ESI) for RSC Advances.
This journal is © The Royal Society of Chemistry 2023

Supplementary Information for

Efficient and Eco-friendly Catalyst-free Synthesis of *N*-Sulfonylimines from Sulfonamides and Aldehydes: Crucial role of Al_2O_3 as a Reusable Dehydrating Agent

Zaineb Litim,[a] Hanen Slimi,[a,b] Thierry Ollevier,[*b] and Jamil Kraïem[*a]

[a.] *Laboratoire de Développement Chimique, Galénique et Pharmacologique des Médicaments, Faculté de Pharmacie de Monastir, Université de Monastir, Rue Avicenne, 5000, Monastir Tunisia. E-mail : jamil.kraiem@fphm.u-monastir.tn*

[b.] *Département de chimie, Université Laval, 1045 avenue de la Médecine, Québec (Québec) G1V 0A6, Canada. E-mail: thierry.ollevier@chm.ulaval.ca*

Table of contents

General procedure for the synthesis of *N*-sulfonylimines 2
References 3
Copies of NMR spectra 14

General methods

^{1}H NMR and ^{13}C NMR spectra were recorded on a Bruker AV 500 MHZ or AV 300 MHZ, using $CDCl_3$ as solvent. Chemical shifts (δ) are expressed in ppm relative to residual solvents ($CDCl_3$ δ_H 7.26, δ_C 77.16*).* In all ^{1}H NMR spectra, the number of protons for each signal and coupling constants values (Hertz) are indicated. Multiplicity is also reported and designated by the following abbreviations: s (singlet), d (doublet), t (triplet), q (quartet) m (multiplet). High-resolution mass spectroscopy (HRMS) was performed on a Q Extractive mass Spectrometer (Thermo Fisher). Melting points were measured with Kofler heating bench. Thin-layer chromatography (TLC) was performed on Merck 60F-254 precoated silica gel sheets (0.25 mm). Dimethyl carbonate (anhydrous ≥ 99%) and alumine (aluminum oxide, activated, neutral, 150 mesh, 58 Å, Fe_2O_3 max 0.03%) were purchased from Sigma-Aldrich. All chemicals obtained from commercial suppliers were used without further purification, except aldehydes were distilled or recrystallized (solid reagents) before use. Anhydrous solvents were prepared according to the standard methods. Alumine was activated at 200°C under reduced pressure (2 mbar) overnight before use.

General procedure for the synthesis of *N*-sulfonylimines

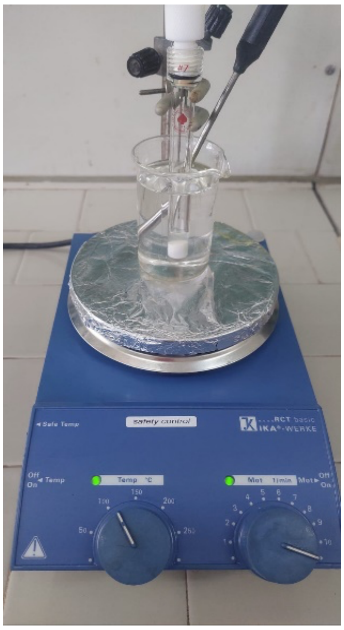

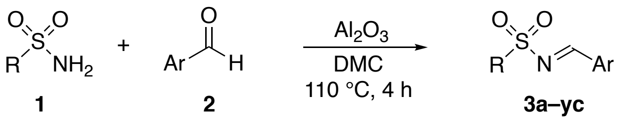

1 **2** **3a–yc**

A stirred mixture of sulfonamide **1** (1.2 mmol), aldehyde **2** (1 mmol), and 2 equiv. of activated Al_2O_3 in anhydrous DMC (1 mL) was heated in an 8-mL pressure tube at 110 °C for 4 h (reaction monitored by TLC: cyclohexane/AcOEt 8:3). After the reaction was complete, the mixture was filtered, and aluminum oxide was then washed thoroughly with 4 mL of hot DMC (60 °C). The collected filtrate was concentrated with the rotary evaporator under reduced pressure and crystallized from ethanol/water to yield pure *N*-sulfonylimines **3**. The DMC fractions, recovered after evaporation with the rotary evaporator, were subjected to fractional distillation at atmospheric pressure.

N-Sulfonylimines **3a**,[1] **3b**,[1] **3c**,[2] **3d**,[2] **3e**,[1] **3f**,[1] **3g**,[1] **3h**,[2] **3i** ,[3] **3j** ,[1] **3k**,[1] **3l** ,[1] **3m**,[1] **3n**,[2] **3o**,[2] **3p**,[1] **3q**,[2] **3r**,[4] **3s**,[5] **3t**,[5] **3v**,[5] **and 3x**[6] are known compounds and previously reported in the literature.

N-sulfonylimines **3u**, **3w**, **3ya**, **3yb**, and **3yz** are new compounds.

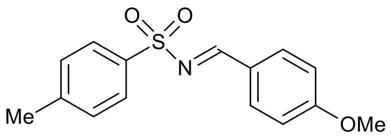

3a

***(E)-N*-(4-methoxybenzylidene)-4-methylbenzenesulfonamide 3a.**[1] According to the general procedure, the product was isolated as white solid; m = 269 mg; 93% (mp = 126–128 °C); ^{1}H NMR (300 MHz; $CDCl_3$) δ_H 8.94 (3H, s), 7.95–7.82 (4H, m), 7.33 (2H, d, *J* = 8.1 Hz), 6.96, (2H, d, *J* = 9 Hz), 3.88 (3H, s), 2.43 (3H, s); ^{13}C NMR (75 MHz; $CDCl_3$) δ 169.3, 165.4, 144.4, 135.9, 133.9, 129.9, 128.0, 125.4, 114.8, 55.8, 21.8.

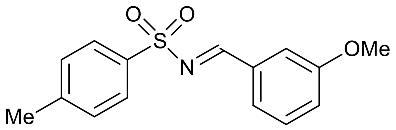

3b

***(E)-N*-(3-methoxybenzylidene)-4-methylbenzenesulfonamide 3b.**[1] According to the general procedure, the product was isolated as white solid; m = 270 mg; 93% (mp = 56–58 °C); ^{1}H NMR (300 MHz; $CDCl_3$) δ_H 8.98 (1H, s), 7.89 (2H, d, *J* = 8.1 Hz), 7.51–7.31 (5H, m), 7.20–7.10 (1H, m), 3.83 (3H, s), 2.44 (3H, s); ^{13}C NMR (75 MHz; $CDCl_3$) δ 170.3, 160.2, 144.8, 135.2, 133.8, 130.2, 130.0, 128.3, 125.5, 122.3, 113.4, 55.7, 21.8.

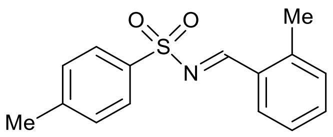

3c

***(E)*-4-methyl-*N*-(2-methylbenzylidene)benzenesulfonamide 3c.**[2] According to the general procedure, the product was isolated as white solid; m = 254 mg; 93% (mp = 91–92 °C); ^{1}H NMR (300 MHz; $CDCl_3$) δ_H 9.34 (1H, s), 8.00 (1H, d, *J* = 7.8 Hz), 7.89 (2H, d, *J* = 8.1 Hz), 7.47 (1H, t, *J* = 7.5 Hz), 7.34 (2H, d, *J* = 7.8 Hz), 7.31–7.20 (2H, m), 2.60 (3H, s), 2.43 (3H, s); ^{13}C NMR (75 MHz; $CDCl_3$) δ 168.8, 144.6, 142.4, 135.5, 134.7, 131.7, 130.8, 130.5, 129.9, 128.1, 126.7, 21.8, 19.8.

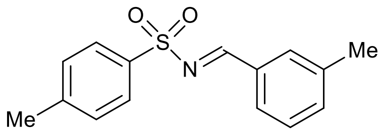

3d

***(E)*-4-methyl-*N*-(3-methylbenzylidene)benzenesulfonamide 3d.**[2] According to the general procedure, the product was isolated as white solid; m = 255 mg; 93% (mp = 88–90 °C); ^{1}H NMR (300 MHz; $CDCl_3$) δ_H 8.99 (1H, s), 7.88 (2H, d, *J* = 8.4 Hz), 7.76 (1H, s), 7.70 (1H, d, *J* = 7.2 Hz), 7.46–7.31 (4H, m), 2.43 (3H, s), 2.38 (3H, s); ^{13}C NMR (75 MHz; $CDCl_3$) δ 170.5, 144.7, 139.2, 136.0, 135.3, 132.5, 131.5, 129.9, 129.1, 128.2, 21.8, 21.3.

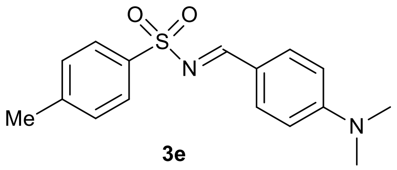

3e

***(E)*-*N*-(4-(dimethylamino)benzylidene)-4-methylbenzenesulfonamide 3e.**[1] According to the general procedure, the product was isolated as yellow solid; m = 287 mg; 95% (mp = 171–173 °C); ^{1}H NMR (300 MHz; $CDCl_3$) δ_H 8.81 (1H, s), 7.85 (2H, d, *J* = 8.4 Hz), 7.76 (2H, d, *J* = 9.0 Hz), 7.29 (2H, d, *J* = 8.4 Hz), 6.65 (2H, d, *J* = 9.0 Hz), 3.09 (6 H, s), 2.41 (3H, s); ^{13}C NMR (75 MHz; $CDCl_3$) δ 169.1, 155.0, 143.7, 137.0, 134.1, 129.7, 127.7, 119.9, 111.4, 40.2, 21.7.

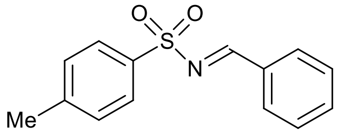

3f

***(E)-N*-benzylidene-4-methylbenzenesulfonamide 3f.**[1] According to the general procedure, the product was isolated as white solid; m = 249 mg; 96% (mp = 111–113 °C); ^{1}H NMR (300 MHz; $CDCl_3$) δ_H 9.03 (1H, s), 7.92 (2H, d, *J* = 7.2 Hz), 7.89 (2H, d, *J* = 8.4 Hz) 7.67–7.55 (1H, m), 7.53–7.45 (2H, m), 7.34 (2H, d, *J* = 7.8 Hz), 2.43 (3H, s); ^{13}C NMR (75 MHz; $CDCl_3$) δ 170.3, 144.7, 135.4, 135.0, 132.6, 131.4, 129.9, 129.3, 128.2, 21.8.

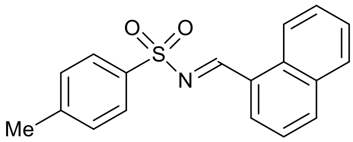

3g

***(E)*-4-methyl-*N*-(naphthalen-1-ylmethylene)benzenesulfonamide 3g.**[1] According to the general procedure, the product was isolated as white solid; m = 269 mg; 87% (mp = 133–135 °C); ^{1}H NMR (300 MHz; $CDCl_3$) δ_H 9.61 (1H, s), 8.99 (1H, d, *J* = 8.7 Hz), 8.15 (1H, dd, *J* = 7.3 Hz, *J* = 1.2 Hz), 8.10 (1H, d, *J* = 8.2 Hz), 8.00–7.87 (3H, m), 7.72–7.63 (1H, m), 7.62–7.53 (2H, m), 7.36 (2H, d, *J* = 8.0 Hz), 2.43 (3H, s); ^{13}C NMR (75 MHz; $CDCl_3$) δ 169.9, 144.7, 136.3, 135.6, 135.3, 133.9, 132.0, 130.0, 129.2, 129.1, 128.2, 127.8, 127.1, 125.2, 124.4, 21.8.

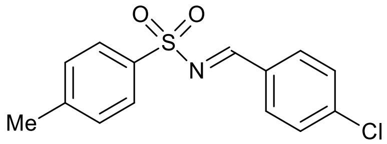

3h

***(E)-N*-(4-chlorobenzylidene)-4-methylbenzenesulfonamide 3h.**[2] According to the general procedure, the product was isolated as white solid; m = 264 mg; 90% (mp = 174–176 °C); ^{1}H NMR (300 MHz; $CDCl_3$) δ_H 8.99 (1H, s), 7.88 (2H, d, *J* = 8.5 Hz), 7.86 (2H, d, *J* = 9.0 Hz) 7.46 (2H, d, *J* = 8.5 Hz), 7.35 (2H, d, *J* = 8.0 Hz), 2.44 (3H, s); ^{13}C NMR (75 MHz; $CDCl_3$) δ 168.8, 144.9, 141.6, 135.0, 132.5, 130.9, 130.0, 129.7, 128.3, 21.8.

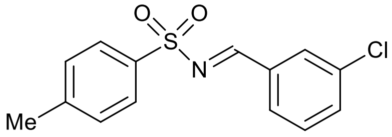

3i

***(E)-N*-(3-chlorobenzylidene)-4-methylbenzenesulfonamide 3i.**[3] According to the general procedure, the product was isolated as white solid ; m = 243 mg; 83% (mp = 80–82°C); ^{1}H NMR (300 MHz; CDCl$_3$) δ_H 8.97 (1H, s), 7.92 (1H, t, *J* = 1.8 Hz), 7.88 (2H, d, *J* = 8.1), 7.77 (1H, dt, *J* = 7.7 Hz, *J* = 1.3 Hz), 7.56 (1H, ddd, *J* = 8.1 Hz, *J* = 2.1 Hz, *J* = 1.2), 7.43 (1H, t, *J* = 7.8 Hz), 7.35 (2H, d, J = 8.1 Hz), 2.44 (3H, s); ^{13}C NMR (75 MHz; CDCl$_3$) δ 168.7, 145.0, 135.5, 134.8, 134.1, 130.5, 130.3, 130.0, 129.9, 128.3, 126.6, 21.8.

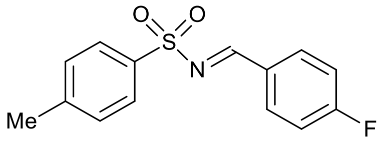

3j

***(E)-N*-(4-fluorobenzylidene)-4-methylbenzenesulfonamide 3j.**[1] According to the general procedure, the product was isolated as white solid ; m = 252 mg; 91% (mp = 109–111 °C); ^{1}H NMR (300 MHz; CDCl$_3$) δ_H 8.99 (1H, s), 7.95 (2H, dd, *J* = 8.7 Hz, *J* = 5.4 Hz), 7.88 (2H, d, *J* = 8.4 Hz), 7.35 (2H, d, *J* = 8.1 Hz), 7.17 (2H, t, *J* = 8.6 Hz), 2.44 (3H, s); ^{13}C NMR (75 MHz; CDCl$_3$) δ 168.7, 167.0 (d, *J* = 257.0 Hz), 144.8, 135.2, 133.9 (d, *J* = 9.8 Hz), 130.0, 128.9 (d, *J* = 3.0 Hz), 128.2, 116.8 (2C, d, *J* = 22.5), 21.8.

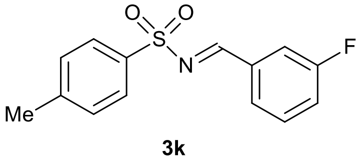

3k

***(E)-N*-(3-fluorobenzylidene)-4-methylbenzenesulfonamide 3k.**[1] According to the general procedure, the product was isolated as white solid ; m = 255 mg; 92% (mp = 82–84 °C); ^{1}H NMR (300 MHz; CDCl$_3$) δ_H 9.00 (1H, d, *J* = 1.5 Hz), 7.88 (2H, d, *J* = 8.4 Hz), 7.71–7.61 (2H, m), 7.53–7.43 (1H, m), 7.35 (2H, d, *J* = 8.1 Hz), 7.32–7.27 (1H, m), 2.44 (3H, s); ^{13}C NMR (75 MHz; CDCl$_3$) δ 168.8 (d, *J* = 2.3 Hz), 163.0 (d, *J* = 247.7 Hz), 145.0, 134.9, 134.6 (d, *J* = 7.5 Hz), 130.9 (d, *J* = 8.3 Hz), 130.0, 128.3, 128.0 (d, *J* = 3.0 Hz), 122.1 (d, *J* = 21.8 Hz), 116.7 (d, *J* = 22.5 Hz), 21.8.

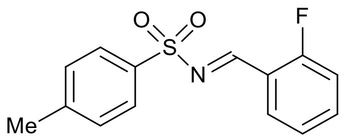

3l

***(E)*-*N*-(2-fluorobenzylidene)-4-methylbenzenesulfonamide 3l.**[1] According to the general procedure, the product was isolated as white solid ; m = 244 mg; 88% (mp = 135–137 °C); ^{1}H NMR (300 MHz; $CDCl_3$) δ_H 9.35 (1H, s), 8.16–8.00 (1H, m), 7.89 (2H, d, *J* = 8.4 Hz), 7.68–7.53 (1H, m), 7.35 (2H, d, *J* = 8.1 Hz), 7.23 (1H, t, *J* = 7.7 Hz), 7.19–7.11 (1H, m), 2.44 (3H, s); ^{13}C NMR (75 MHz; $CDCl_3$) δ 164.4 (d, *J* = 258,8 Hz), 163.8 (d, *J* = 6.0 Hz), 144.9, 137.1 (d, *J* = 9.0 Hz), 134.9, 130.0, 129.5 (d, *J* = 0.8 Hz), 128.4, 125.0 (d, *J* = 3.8 Hz), 120.6 (d, *J* = 8.3 Hz), 116.5 (d, *J* = 21.0 Hz), 21.8.

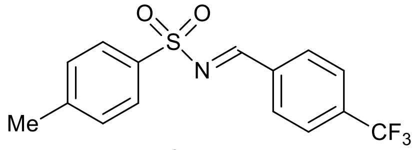

3m

***(E)*-4-methyl-*N*-(4-(trifluoromethyl)benzylidene)benzenesulfonamide 3m.**[1] According to the general procedure, the product was isolated as white solid ; m = 284 mg; 87% (mp = 154–156 °C); ^{1}H NMR (300 MHz; $CDCl_3$) δ_H 9.07 (1H, s), 8.04 (2H, d, *J* =8.1 Hz), 7.90 (2H, d, *J* = 8.1 Hz), 7.74 (2H, d, *J* = 8.1 Hz), 7.36 (2H, d, *J* = 8.1 Hz), 2.44 (3H, s); ^{13}C NMR (75 MHz; $CDCl_3$) δ 168.5, 145.2, 135.9 (q, *J* = 33.0 Hz), 135.7, 134.9, 131.4, 130.1, 128.4, 126.2 (q, *J* = 3.8 Hz), 123.5 (q, *J* = 271.0 Hz), 21.7.

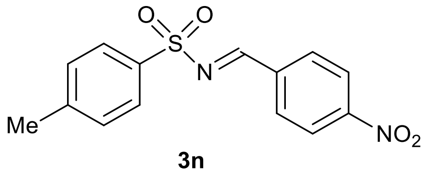

3n

***(E)*-4-methyl-*N*-(4-nitrobenzylidene)benzenesulfonamide 3n.**[2] According to the general procedure, the product was isolated as yellow solid; m = 188 mg; 62% (mp = 207–209 °C); ^{1}H NMR (300 MHz; $CDCl_3$) δ_H 9.10 (1H, s), 8.33 (2H, d, *J* = 8.9 Hz), 8.11 (2H, d, *J* = 8.9 Hz), 7.90 (2H, d, *J* = 8.4 Hz), 7.38 (2H, d, *J* = 8.1 Hz), 2.46 (3H, s); ^{13}C NMR (75 MHz; $CDCl_3$) δ 167.4, 151.3, 145.5, 138.0, 134.3, 132.0, 130.2, 128.5, 124.3, 21.9.

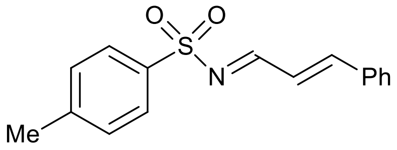

3o

***(E)*-4-methyl-*N*-(*(E)*-3-phenylallylidene)benzenesulfonamide 3o.**[2] According to the general procedure, the product was isolated as yellow solid ; m = 256 mg; 90% (mp = 110–112 °C); ^{1}H NMR (300 MHz; $CDCl_3$) δ_H 8.77 (1H, d, *J* = 9.4 Hz), 7.86 (2H, d, *J* = 8.3 Hz), 7.60–7.53 (2H, m), 7.49 (1H, d, *J* = 15.9 Hz), 7.45–7.38 (3H, m), 7.34 (2H, d, *J* = 8.0 Hz), 6.98 (1H, dd, *J* = 15.8 Hz, *J* = 9.4 Hz), 2.43 (3H, s); ^{13}C NMR (75 MHz; $CDCl_3$) δ 171.0, 154.0, 144.6, 135.5, 134.3, 131.8, 129.9, 129.3, 128.8, 128.1, 124.9, 21.8.

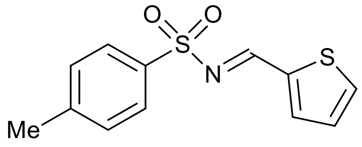

3p

***(E)*-4-methyl-*N*-(thiophen-2-ylmethylene)benzenesulfonamide 3p.**[1] According to the general procedure, the product was isolated as yellow solid; m = 244 mg; 92% (mp = 98–100 °C); ^{1}H NMR (300 MHz; $CDCl_3$) δ_H 9.11 (1H, s), 7.86 (2H, d, *J* = 8.1 Hz), 7.77 (2H, d, *J* = 4.2 Hz), 7.33 (2H, d, *J* = 8.1 Hz), 7.20 (1H, t, *J* = 4.5 Hz), 2.43 (3H, s); ^{13}C NMR (75 MHz; $CDCl_3$) δ 162.4, 144.6, 139.2, 138.3, 136.8, 135.6, 129.9, 129.0, 128.1, 21.8.

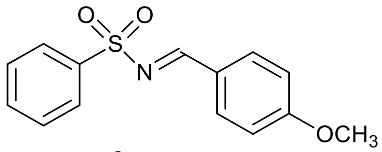

3q

***(E)*-*N*-(4-methoxybenzylidene)benzenesulfonamide 3q.**[2] According to the general procedure, the product was isolated as white solid; m = 256 mg; 93% (mp = 130–132 °C); ^{1}H NMR (300 MHz; $CDCl_3$) δ_H 8.96 (1H, s), 8.10–7.94 (2H, m), 7.94–7.80 (2H, m), 7.72–7.57 (1H, m), 7.57–7.49 (2H, m), 7.07–6.85 (2H, m), 3.88 (3H, s); ^{13}C NMR (75 MHz; $CDCl_3$) δ 169.7, 165.4, 138.8, 133.9, 133.3, 129.1, 127.9, 125.2, 114.8, 55.7.

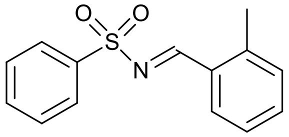

3r

***(E)-N*-(2-methylbenzylidene)benzenesulfonamide 3r.**[4] According to the general procedure, the product was isolated as white solid; m = 230 mg; 89% (mp = 77–79 °C); ^{1}H NMR (300 MHz; $CDCl_3$) δ_H 9.36 (1H, s), 8.10–7.96 (3H, m), 7.67–7.44 (4H, m), 7.34–7.22 (2H, m), 2.61 (3H, s); ^{13}C NMR (75 MHz; $CDCl_3$) δ 169.3, 142.5, 138.5, 134.9, 133.6, 131.7, 130.8, 130.4, 129.3,128.0, 126.8, 19.8.

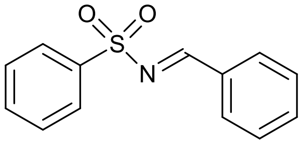

3s

***(E)-N*-benzylidenebenzenesulfonamide 3s.**[5] According to the general procedure, the product was isolated as white solid; m = 237 mg; 97% (mp = 77–79°C); ^{1}H NMR (300 MHz; $CDCl_3$) δ_H 9.06 (1H, s), 8.12–7.97 (2H, m), 7.96–7.90 (2H, m), 7.66–7.46 (6H, m); ^{13}C NMR (75 MHz; $CDCl_3$) δ 170.8, 138.3, 135.2, 133.7, 132.4, 131.5, 129.3, 128.2.

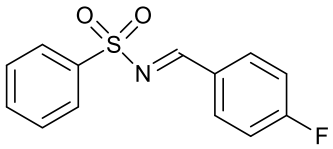

3t

***(E)-N*-(4-fluorobenzylidene)benzenesulfonamide 3t.**[5] According to the general procedure, the product was isolated as white solid; m = 216 mg; 82% (mp = 110–112 °C); ^{1}H NMR (300 MHz; $CDCl_3$) δ_H 9.03 (1H, s), 8.10–7.93 (4H, m), 7.70–7.60 (1H, m), 7.59–7.51 (2H, m), 7.18 (2H, t, J = 8.5 Hz); ^{13}C NMR (75 MHz; $CDCl_3$) δ 169.2, 167.0 (d, J = 257.3 Hz), 138.2, 134.0 (d, J = 9.8 Hz), 132.8, 129.3, 128.8 (d, J = 2.8 Hz), 128.1, 116.6 (d, J = 22.1 Hz).

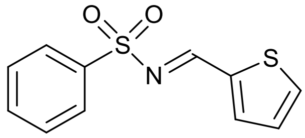

3u

***(E)-N*-(thiophen-2-ylmethylene)benzenesulfonamide 3u.** According to the general procedure, the product was isolated as white solid; m = 236 mg; 94% (mp = 130–132 °C); ^{1}H NMR (300 MHz; $CDCl_3$) δ_H 9.14 (1H, br s), 8.03- 7.91 (2H, m), 7.79 (2H, d, J = 4.2 Hz), 7.66–7.58 (1H, m), 7.57–7.49 (2H, m), 7.21 (1H, t, J = 4.4 Hz); ^{13}C NMR (75 MHz; $CDCl_3$) δ 162.8, 139.4, 138.6, 138.2, 137.1, 133.6, 129.3, 129.1, 128.0; HRMS (TOF MS Cl^+) calculated for $C_{11}H_{10}NO_2S_2$ $[M+H]^+$: 252.0153, found: 252.0151.

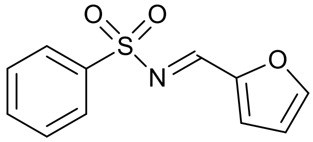

3v

***(E)-N*-(furan-2-ylmethylene)benzenesulfonamide 3v.**[5] According to the general procedure, the product was isolated as white solid; m = 212 mg; 90% (mp = 126–128 °C); ^{1}H NMR (300 MHz; $CDCl_3$) δ_H 8.83 (1H, s), 8.06–7.95 (2H, m), 7.75 (1H, d, J = 1.5 Hz), 7.67–7.58 (1H, m), 7.57–7.48 (2H, m), 7.35 (1H, dd, J = 3,6 Hz, J = 0.6 Hz), 6.65 (1H, dd, J = 3,6 Hz, J = 1,5 Hz); ^{13}C NMR (75 MHz; $CDCl_3$) δ 156.2, 150.0, 149.3, 138.5, 133.6, 129.3, 128.1, 124.9, 113.9.

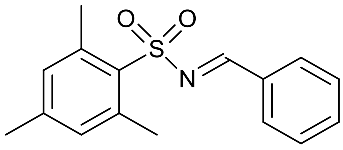

3w

***(E)-N*-benzylidene-2,4,6-trimethylbenzenesulfonamide 3w.** According to the general procedure, the product was isolated as white solid; m = 264 mg; 92% (mp = 87–89 °C); ^{1}H NMR (300 MHz; $CDCl_3$) δ_H 9.03 (1H, s), 7.92 (2H, d, J = 7.2 Hz), 7.61 (1H, t, J = 7.4 Hz), 7.49 (2H, t, J= 7.5 Hz), 6.98 (2H, s), 2.71 (6H, s), 2.31 (3H, s); ^{13}C NMR (75 MHz; $CDCl_3$) δ 169.1, 143.4, 140.4, 134.8, 132.8, 132.3, 132.1, 131.3, 129.3, 23.2, 21.2; HRMS (TOF MS Cl^+) calculated for $C_{16}H_{18}NO_2S$ $[M+H]^+$: 288.1058, found: 288.1062.

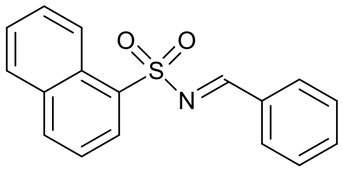

3x

***(E)-N*-benzylidenenaphthalene-1-sulfonamide 3x.**[6] According to the general procedure, the product was isolated as white solid; m = 215 mg; 73% (mp = 121–123 °C); ^{1}H NMR (300 MHz; $CDCl_3$) δ_H 9.16(1H, s), 8.88 (1H, d, *J* = 8.4 Hz), 8.41 (1H, dd, *J* = 7.5 Hz, *J* = 1.1 Hz), 8.11 (1H, d, *J* = 8.1 Hz), 8.00–7.82 (3H, m), 7.77–7.67 (1H, m), 7.65–7.52 (3H, m), 7.50–7.38 (2H, m); ^{13}C NMR (75 MHz; $CDCl_3$) δ 170.8, 135.3, 135.2, 134.3, 133.8, 132.5, 131.5, 129.7, 129.3, 129.2, 129.0, 128.5, 127.1, 125.6, 124.4.

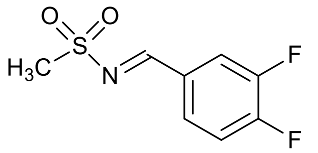

3ya

***(E)-N*-(4-fluorobenzylidene)methanesulfonamide 3ya.** According to the general procedure, the product was isolated as white solid; m = 177 mg; 88% (mp = 109–111 °C); ^{1}H NMR (300 MHz; $CDCl_3$) δ_H 8.99 (1H, s), 7.99 (2H, dd, *J* = 8.8 Hz, *J* = 5.4 Hz), 7.22 (2H, t, *J* = 8.6 Hz), 3.13 (3H, s); ^{13}C NMR (75 MHz; $CDCl_3$) δ 170.2, 167.1 (d, *J* = 257.3 Hz), 140.0 (d, *J* = 9.8 Hz), 128.6 (d, *J* = 3.0 Hz), 116.9 (d, *J* = 22.5 Hz), 40.4; ^{19}F NMR (282 MHz; $CDCl_3$) δ –100.54; HRMS (TOF MS CI^+) calculated for $C_8H_9FNO_2S$ $[M+H]^+$: 202.0338, found: 202.0336.

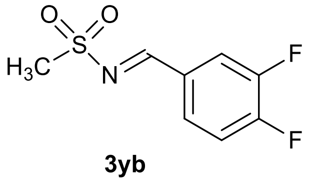

3yb

***(E)-N*-(3,4-difluorobenzylidene)methanesulfonamide 3yb.** According to the general procedure, the product was isolated as white solid; m = 164 mg; 75% (mp = 129–131 °C); ^{1}H NMR (300 MHz; $CDCl_3$) δ_H 8.96 (1H, s), 7.96–7.80 (1H, m), 7.79–7.58 (1H, m), 7.42–7.28 (1H, m), 3.14 (3H, s); ^{13}C NMR (75 MHz; $CDCl_3$) δ 169.4 (d, *J* = 0.8 Hz), 155.0 (dd, *J* = 259.2 Hz, *J* = 12.8 Hz), 151.1 (dd, *J* =251.1 Hz, *J* = 13.3 Hz), 129.4 (dd, *J* = 7.8 Hz, *J* = 3.3 Hz), 119.0 (dd, *J* = 18.2 Hz, *J* = 2.0 Hz), 118.7, 118.4, 40.4; ^{19}F NMR (282 MHz; $CDCl_3$) δ –125.03 (d, *J* = 20.7

Hz), –134.32 (d, J = 20.7 Hz); HRMS (TOF MS CI^+) calculated for $C_8H_8F_2NO_2S$ $[M+H]^+$: 220.0244, found: 220.0240.

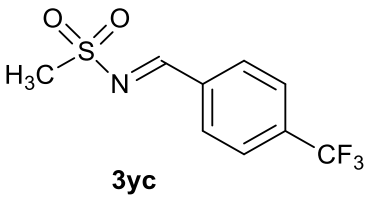

3yc

***(E)-N*-(4-(trifluoromethyl)benzylidene)methanesulfonamide yc.** According to the general procedure, the product was isolated as white solid; m = 208 mg; 83% (mp = 88–90 °C); ^{1}H NMR (300 MHz; $CDCl_3$) δ_H 9.09 (1H, s), 8.09 (2H, d, J = 8.1 Hz), 7.79 (2H, d, J = 8.2 Hz), 3.17 (3H, s); ^{13}C NMR (75 MHz; $CDCl_3$) δ 170.3, 136.2 (1C, q, J = 33.0 Hz), 135.2, 131.5, 126.3 (2C, q, J = 3.8 Hz), 123.4 (1C, q, J = 271.5 Hz), 40.3; ^{19}F NMR (282 MHz; $CDCl_3$) δ –63.32; HRMS (TOF MS CI^+) calculated for $C_9H_9F_3NO_2S$ $[M+H]^+$: 252.0306, found: 252.0308.

Recycling and reuse of alumina

We have found that the use of Al_2O_3 for 2nd, 3rd, 4th, and 5th runs have shown the same efficiency as when it was used for the first time. Recovered Al_2O_3 after the filtration of the reaction crude was activated at 200 °C under reduced pressure (2 mbar) overnight and reused in another reaction. We found that, in all cases, the conversion to imine was complete (100%), and the isolated yield was always between 92 and 96% (product purified by recrystallization from ethanol–water).

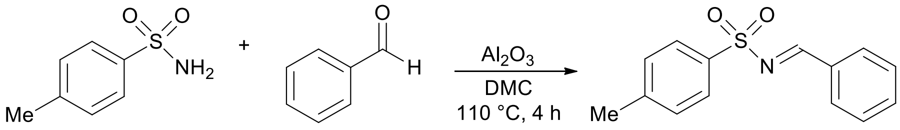

3f

	Conversion (%)	Isolated yield (%)
**Run 1**	100	96
**Run 2**	100	92
**Run 3**	100	95
**Run 4**	100	96
**Run 5**	100	96

References

1 W. W. Yao, R. Li, J. F Li, J Sun, and M.Ye, *Green Chem*. 2019, **21**, 2240–2244.

2 D. Huang, X. Wang, X. Wang, W. Chen, X. Wang, and Y. Hu, *Org. Lett.,* 2016, **18**, 604–607.

3 T. S.Jin, M. J. Yu, L. B. Liu, Y. Zhao, and T. S. Li, *Synth. Commun*., 2006, **36**, 2339–2344.

4 M. C.Kohler, J. M Yost, M. R Garnsey, and D. M. Coltart, *Org. Lett*., 2010, **12**, 3376–3379.

5 D. Chen, X. Chen, T. Du, L. Kong, R. Zhen, S. Zhen, Y. Wen, and G. Zhu, *Tetrahedron Lett*., 2010, **51**, 5131–5133.

6 M. Shi, Y. W. Guo, and H. B. Li, *Chin. J. Chem*., 2007, **25**, 828–835.

Copies of NMR spectra

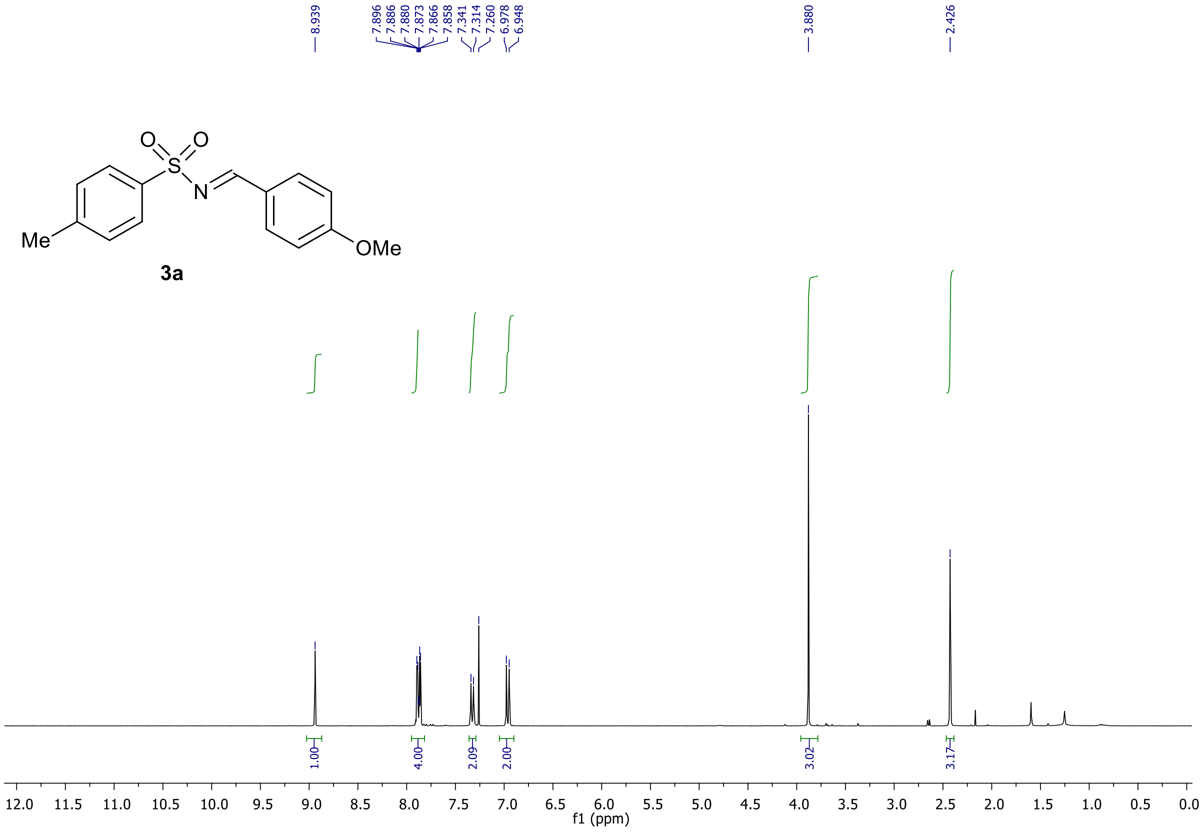

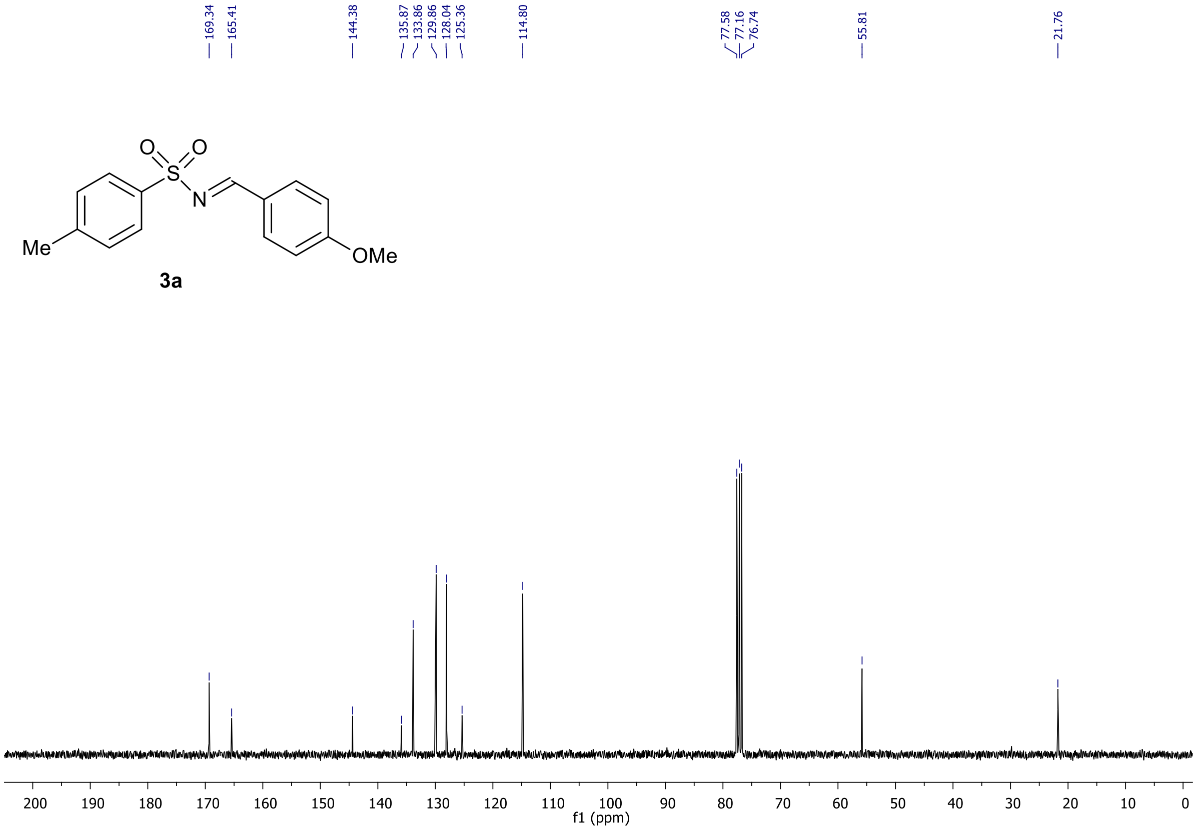

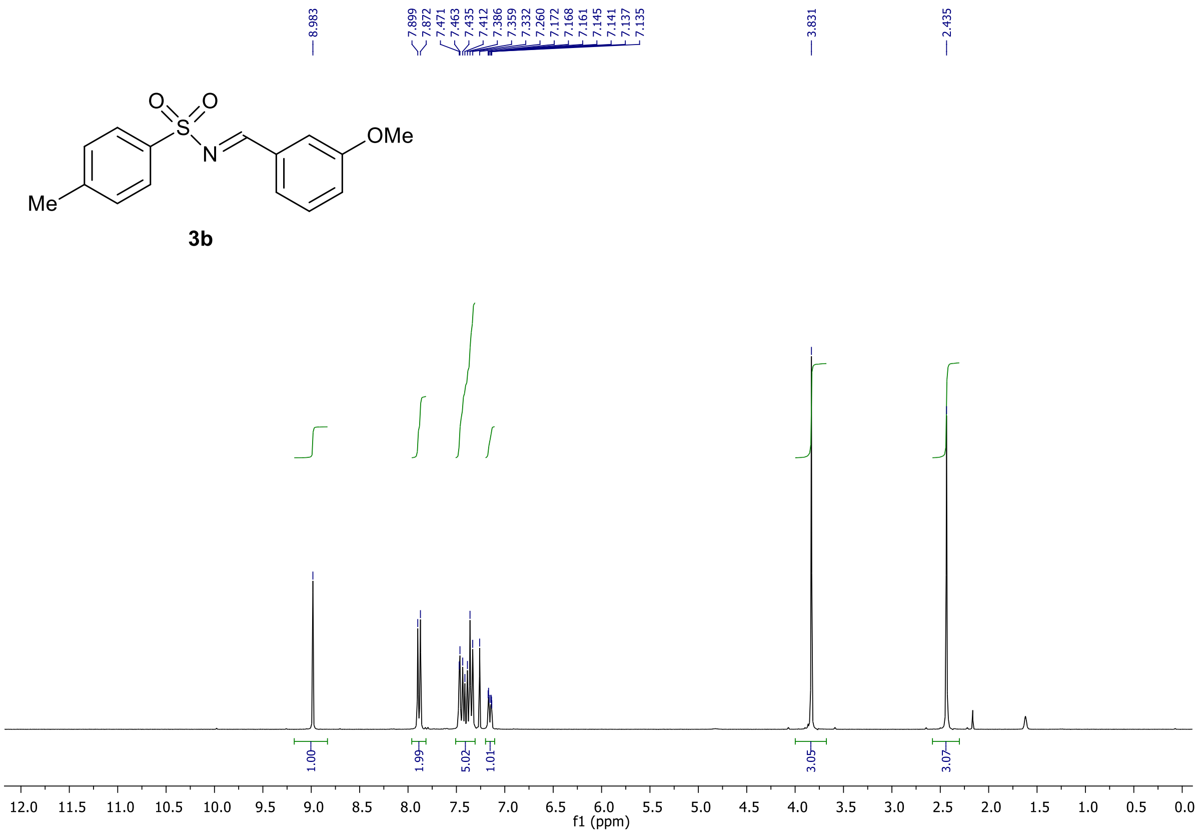
8.983
7.899
7.872
7.471
7.463
7.435
7.412
7.386
7.359
7.332
7.260
7.172
7.168
7.161
7.145
7.141
7.137
7.135
3.831
2.435
OMe
Me
N
S
O
O
3b
1.00
1.99
5.02
1.01
3.05
3.07
f1 (ppm)

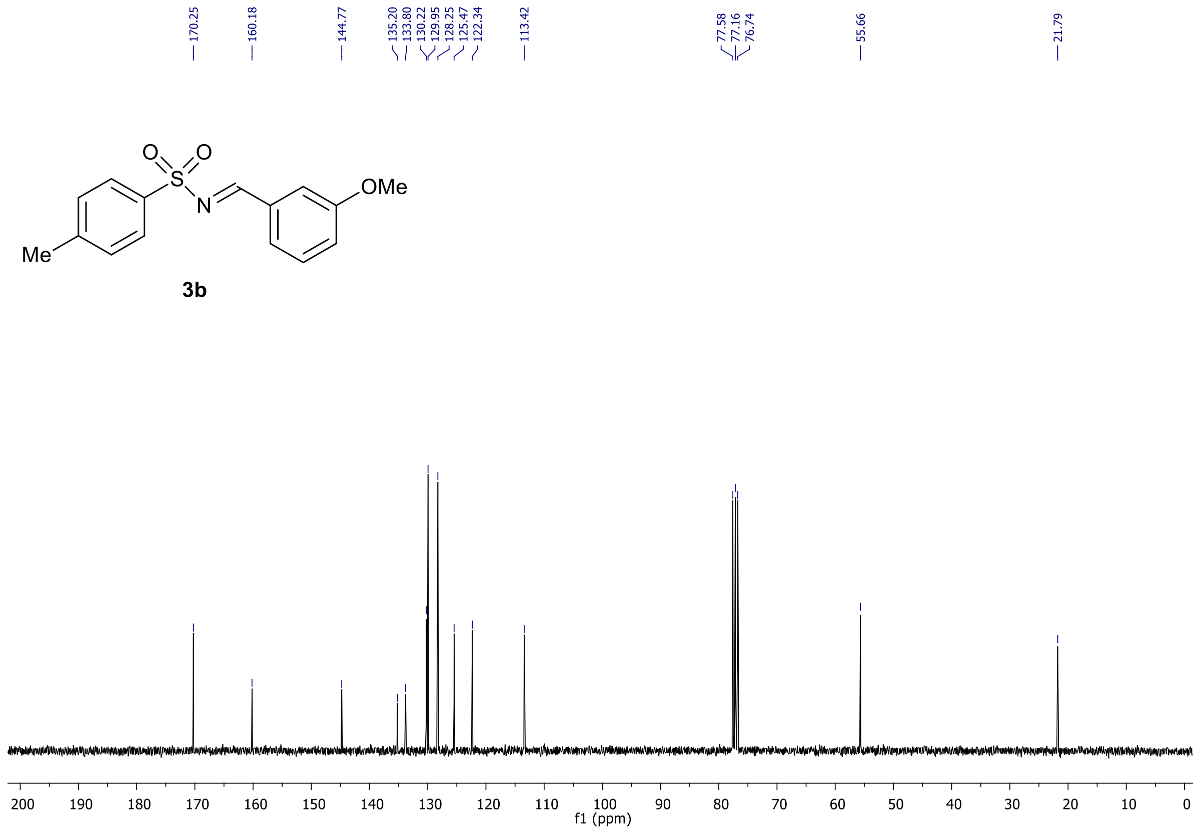
170.25
160.18
144.77
135.20
133.80
130.22
129.95
128.25
125.47
122.34
113.42
77.58
77.16
76.74
55.66
21.79
OMe
Me
N
S
O
O
3b
f1 (ppm)

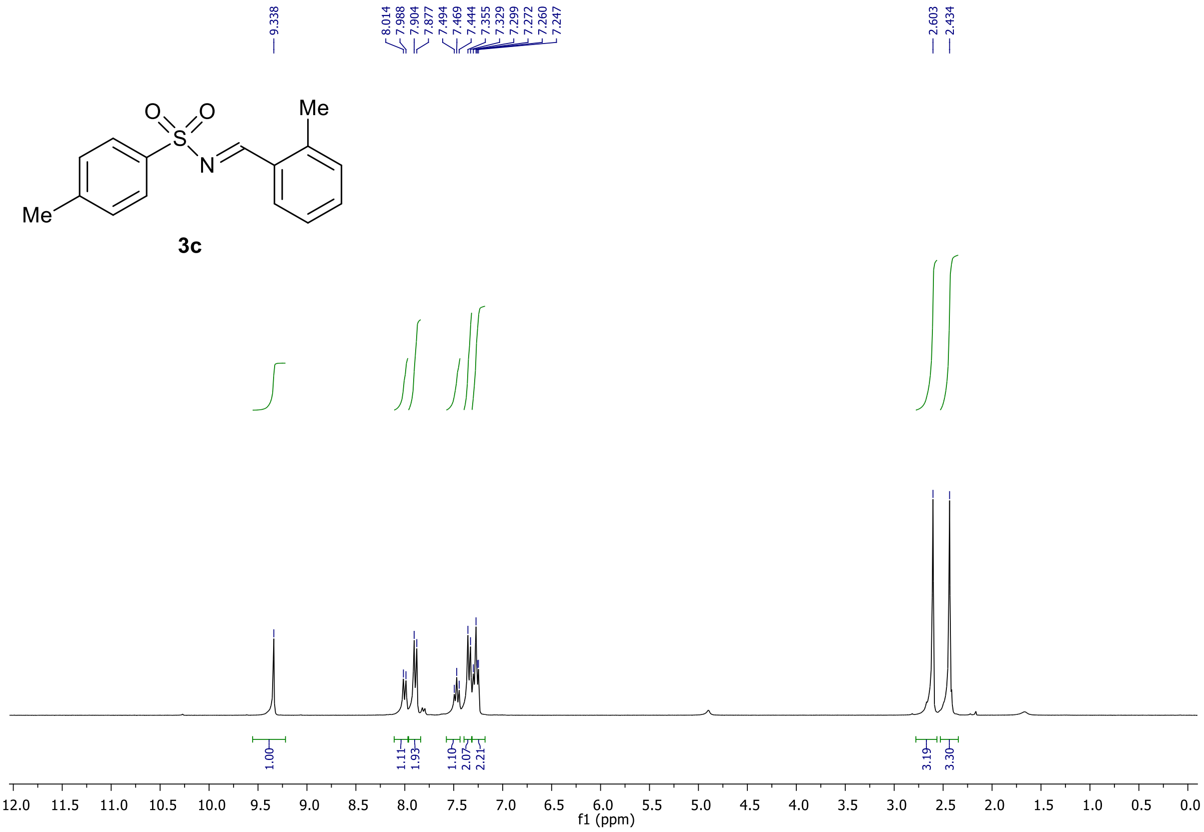
9.338
8.014
7.988
7.904
7.877
7.494
7.469
7.444
7.355
7.329
7.299
7.272
7.260
7.247
2.603
2.434
Me
O
S
O
N
Me
3c
1.00
1.11
1.93
1.10
2.07
2.21
3.19
3.30
12.0 11.5 11.0 10.5 10.0 9.5 9.0 8.5 8.0 7.5 7.0 6.5 6.0 5.5 5.0 4.5 4.0 3.5 3.0 2.5 2.0 1.5 1.0 0.5 0.0
f1 (ppm)

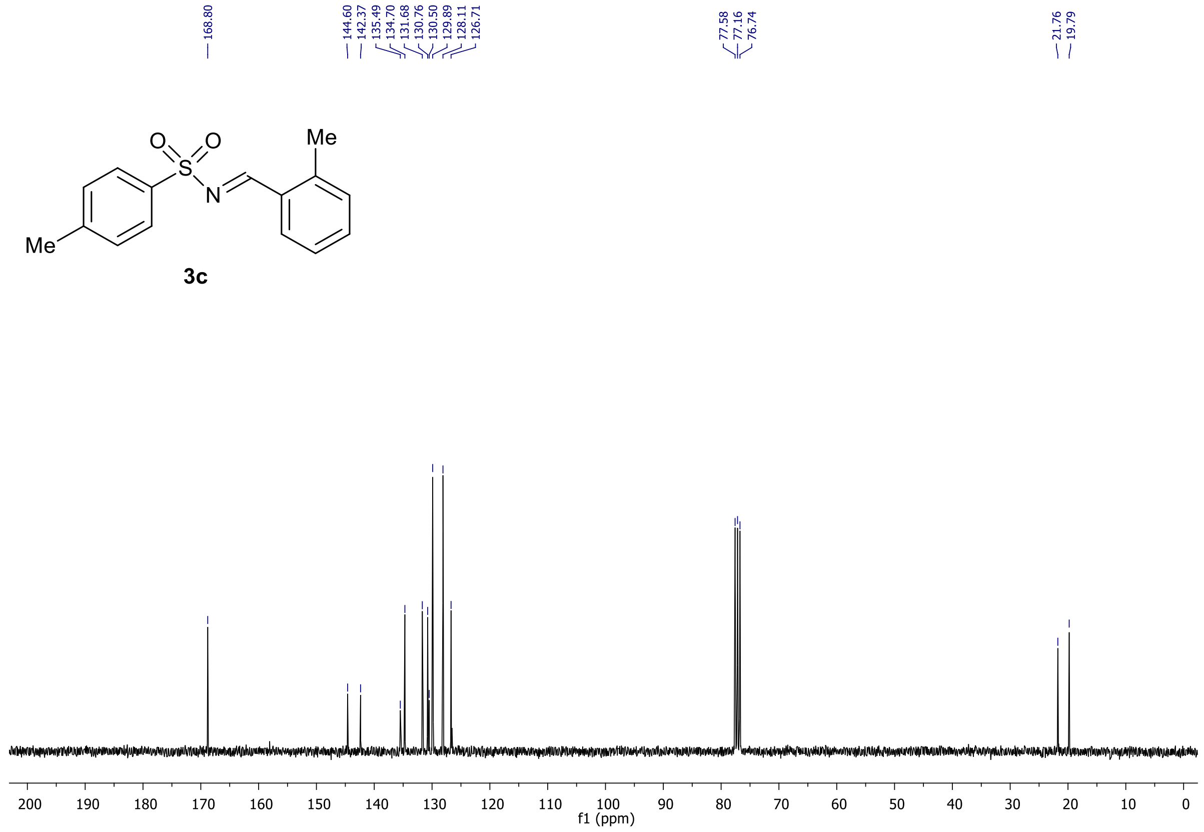
168.80
144.60
142.37
135.49
134.70
131.68
130.76
130.50
129.89
128.11
126.71
77.58
77.16
76.74
21.76
19.79
Me
O
S
O
N
Me
3c
200 190 180 170 160 150 140 130 120 110 100 90 80 70 60 50 40 30 20 10 0
f1 (ppm)

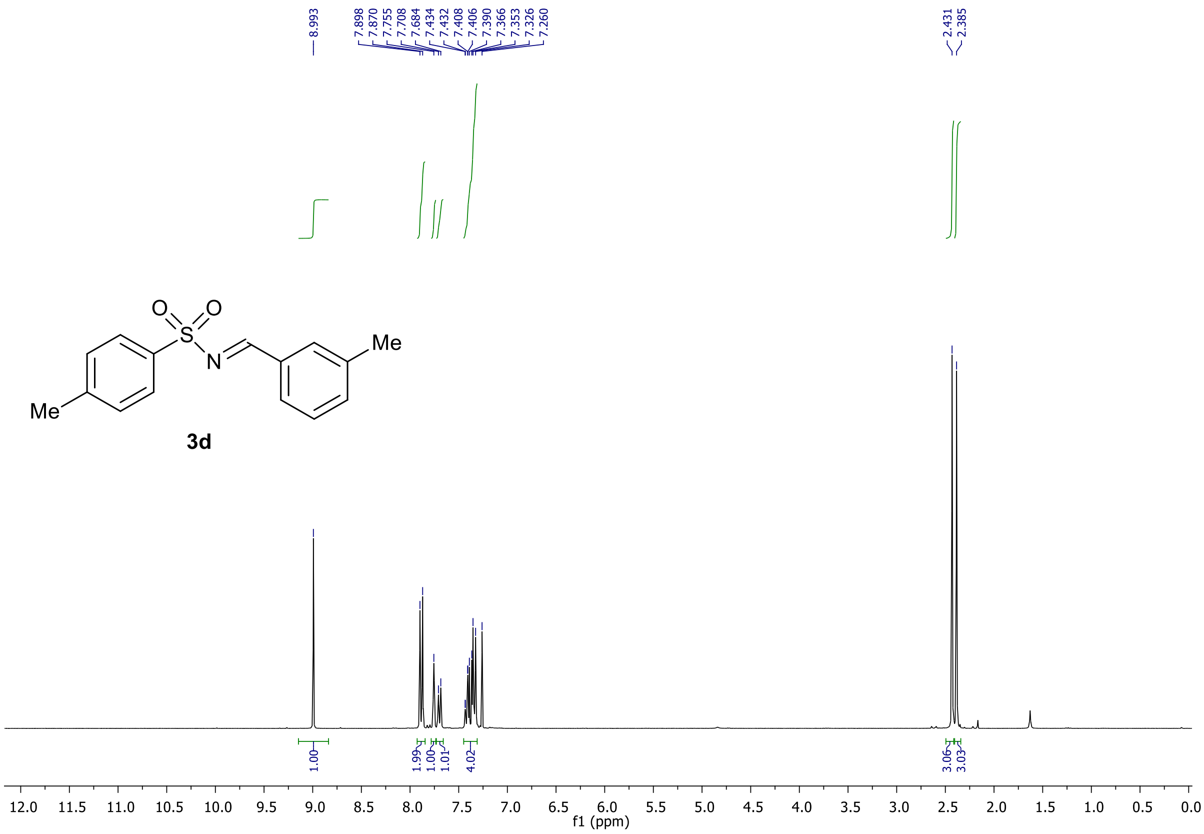
8.993
7.898
7.870
7.755
7.708
7.684
7.434
7.432
7.408
7.406
7.390
7.366
7.353
7.326
7.260
2.431
2.385
Me
Me
3d
1.00
1.99
1.00
1.01
4.02
3.06
3.03
f1 (ppm)

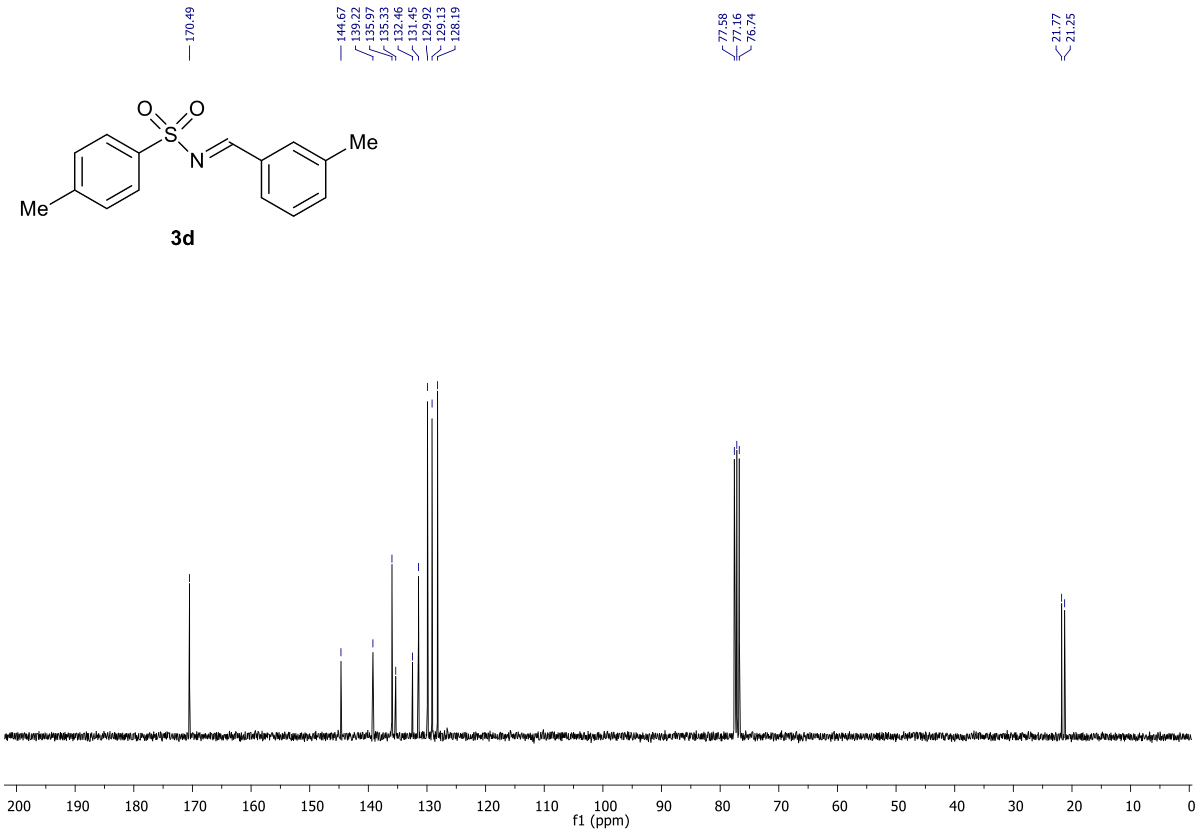
170.49
144.67
139.22
135.97
135.33
132.46
131.45
129.92
129.13
128.19
77.58
77.16
76.74
21.77
21.25
Me
Me
3d
f1 (ppm)

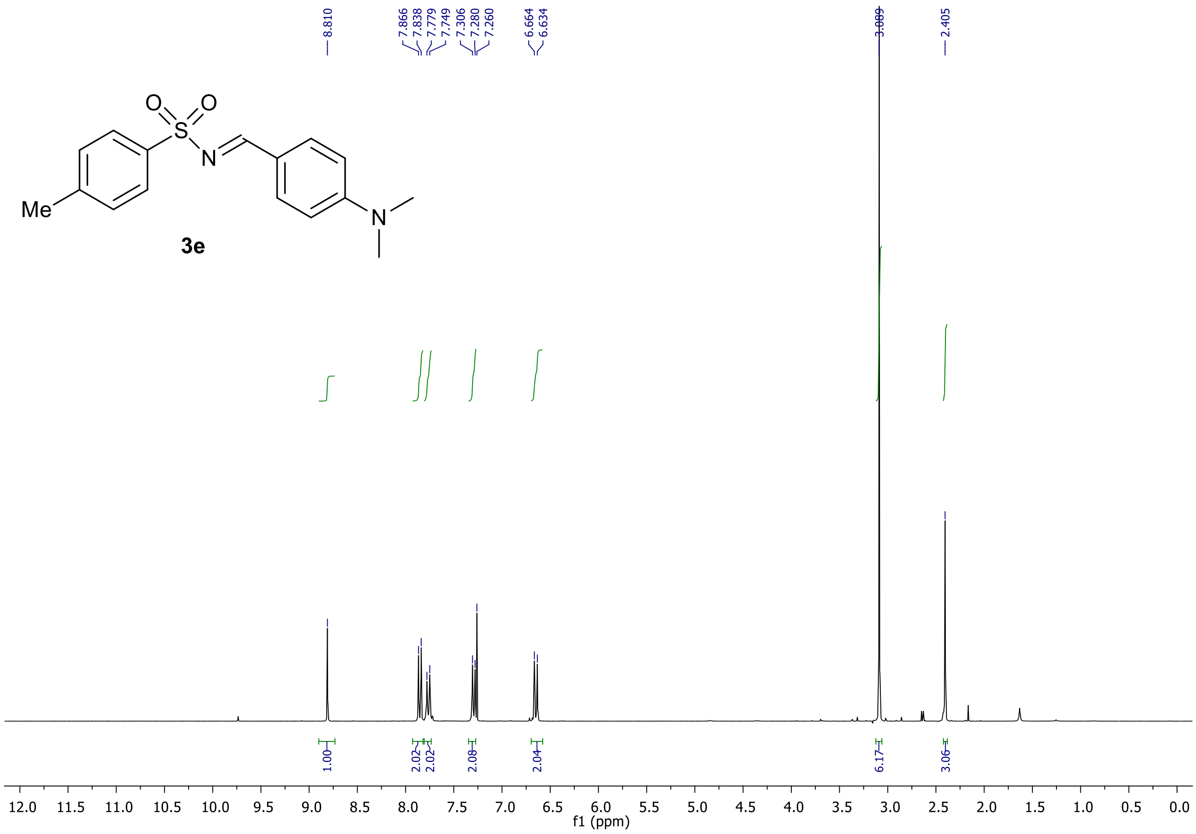
8.810
7.866
7.838
7.779
7.749
7.306
7.280
7.260
6.664
6.634
3.089
2.405
Me
N
S
O
O
N
3e
1.00
2.02
2.02
2.08
2.04
6.17
3.06
12.0 11.5 11.0 10.5 10.0 9.5 9.0 8.5 8.0 7.5 7.0 6.5 6.0 5.5 5.0 4.5 4.0 3.5 3.0 2.5 2.0 1.5 1.0 0.5 0.0
f1 (ppm)

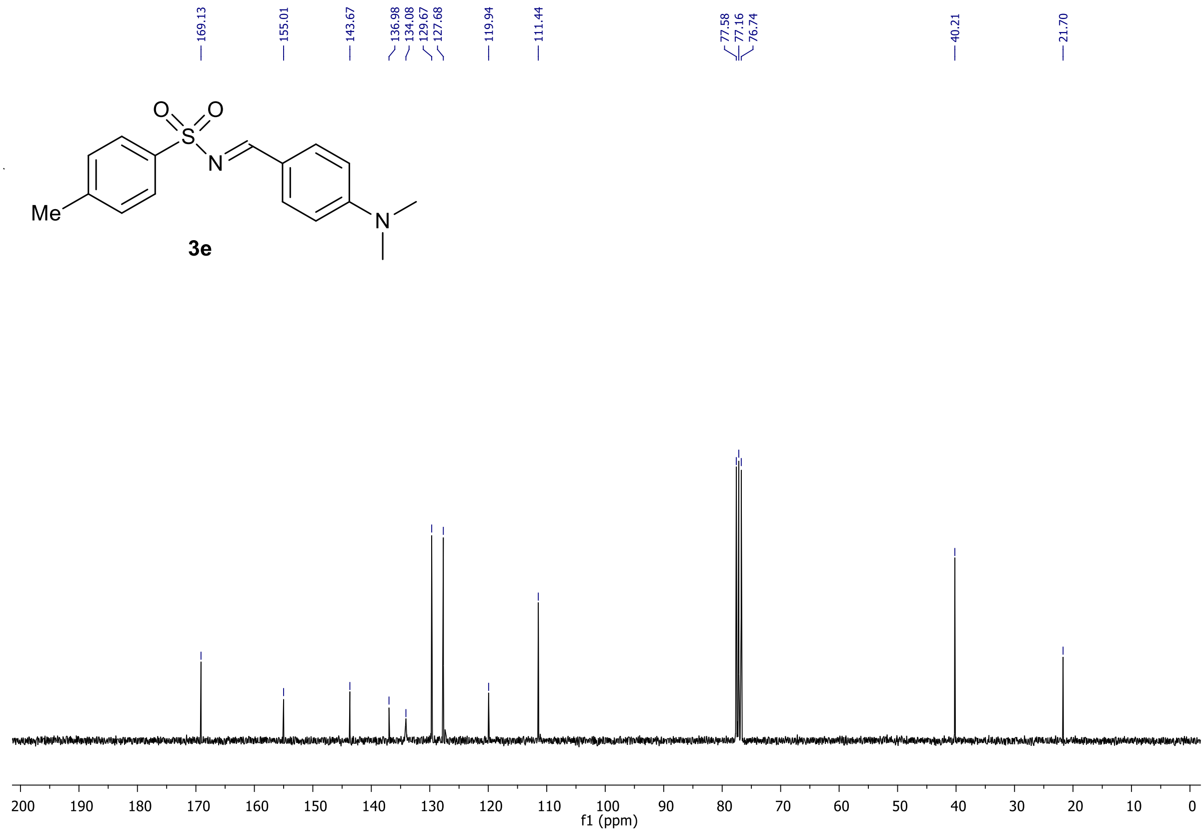
169.13
155.01
143.67
136.98
134.08
129.67
127.68
119.94
111.44
77.58
77.16
76.74
40.21
21.70
Me
N
S
O
O
N
3e
200 190 180 170 160 150 140 130 120 110 100 90 80 70 60 50 40 30 20 10 0
f1 (ppm)

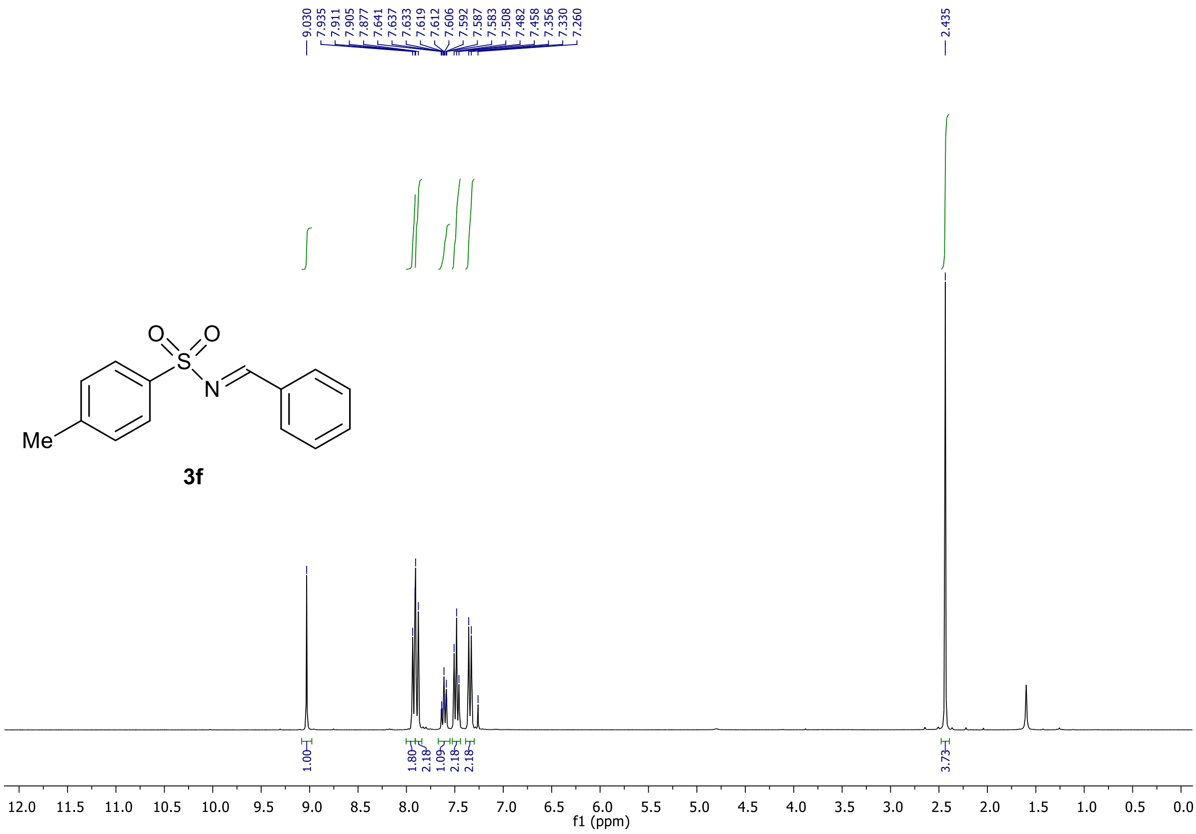
9.030
7.935
7.911
7.905
7.877
7.641
7.637
7.633
7.619
7.612
7.606
7.592
7.587
7.583
7.508
7.482
7.458
7.356
7.330
7.260
2.435
Me
N
S
O
O
3f
1.00
1.80
2.18
1.09
2.18
2.18
3.73
12.0 11.5 11.0 10.5 10.0 9.5 9.0 8.5 8.0 7.5 7.0 6.5 6.0 5.5 5.0 4.5 4.0 3.5 3.0 2.5 2.0 1.5 1.0 0.5 0.0
f1 (ppm)

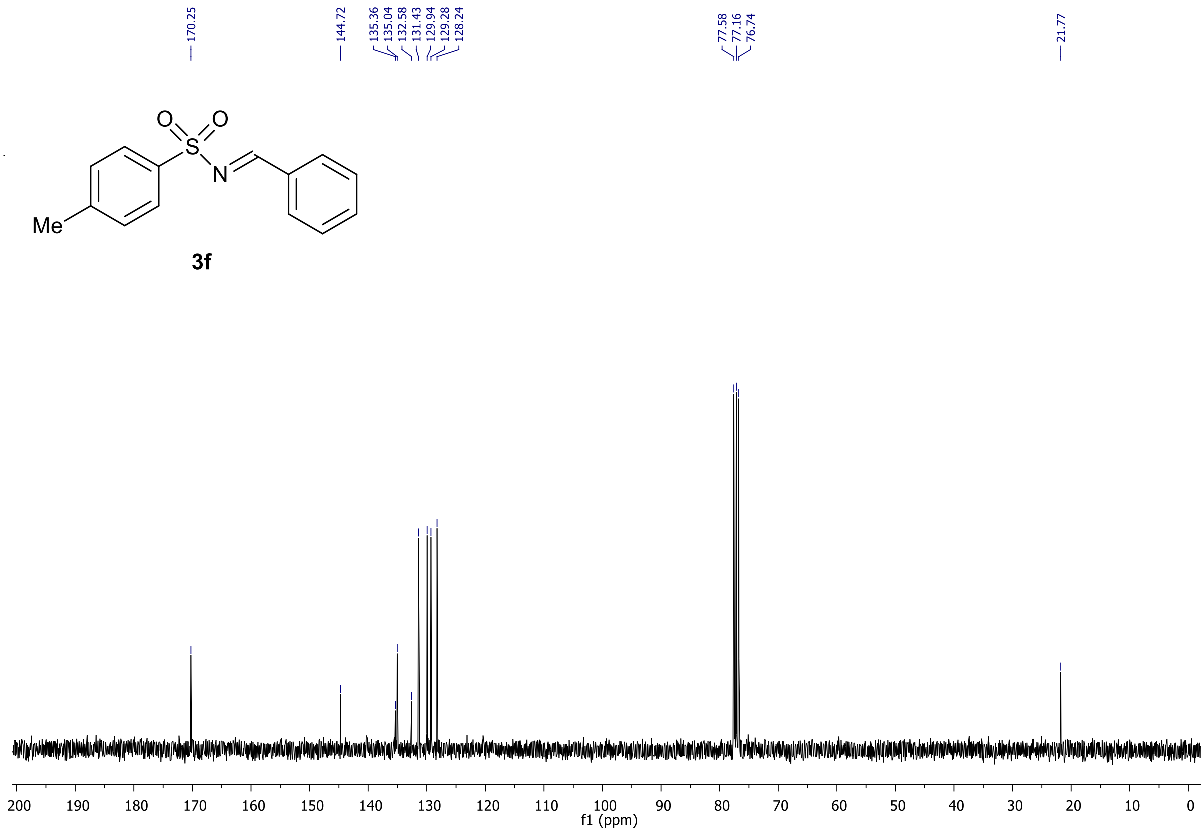
170.25
144.72
135.36
135.04
132.58
131.43
129.94
129.28
128.24
77.58
77.16
76.74
21.77
Me
N
S
O
O
3f
200 190 180 170 160 150 140 130 120 110 100 90 80 70 60 50 40 30 20 10 0
f1 (ppm)

3g

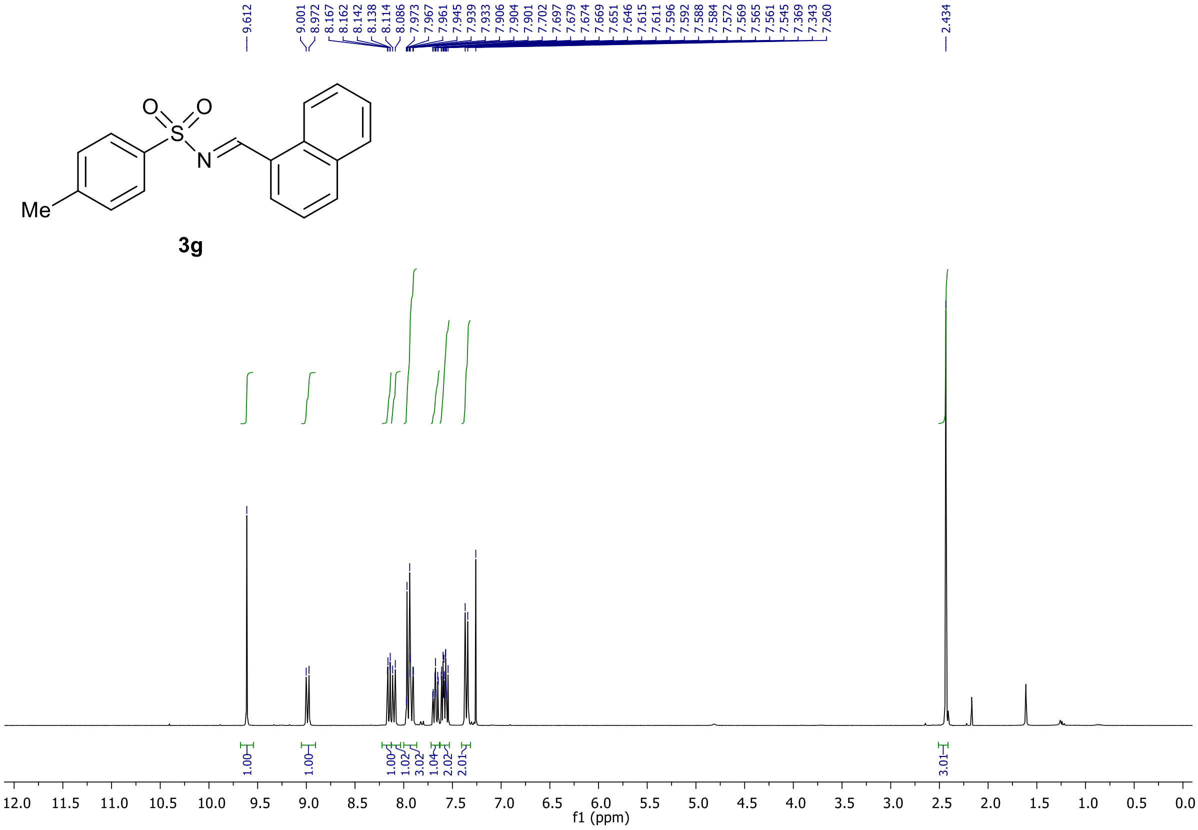

3g

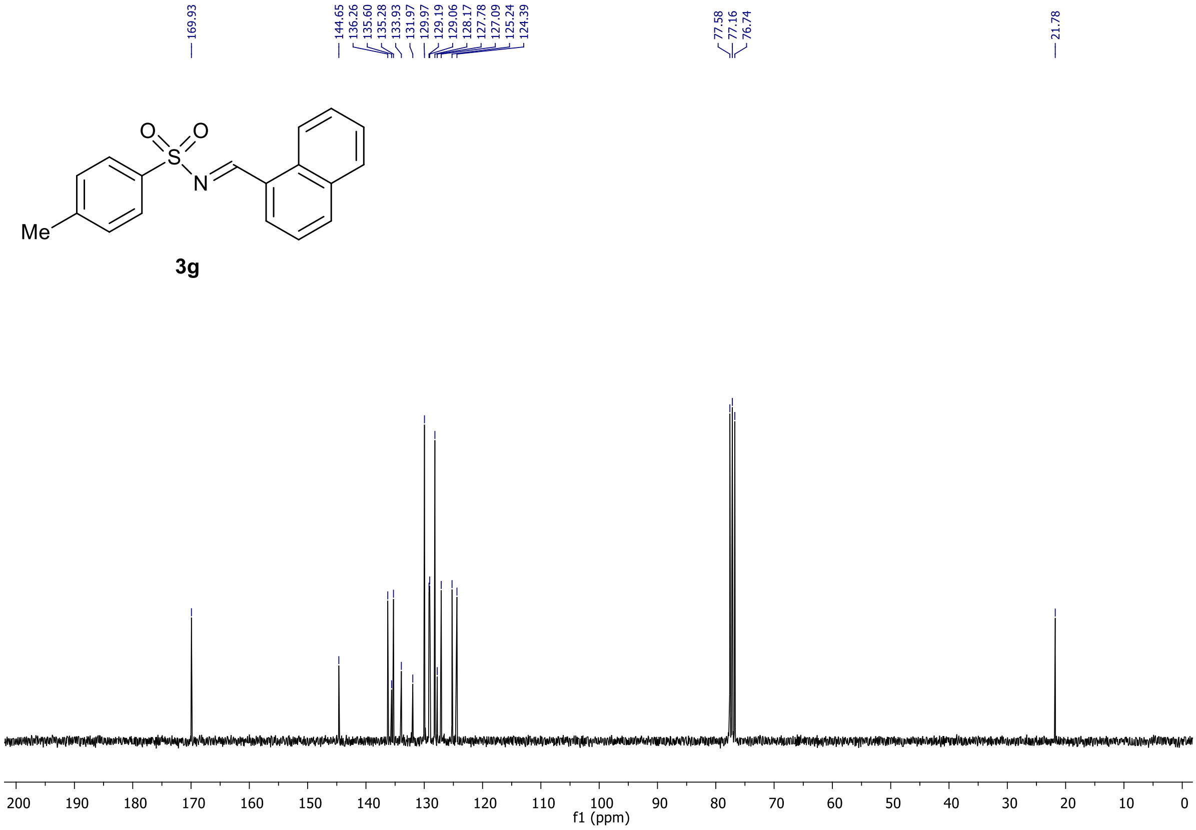

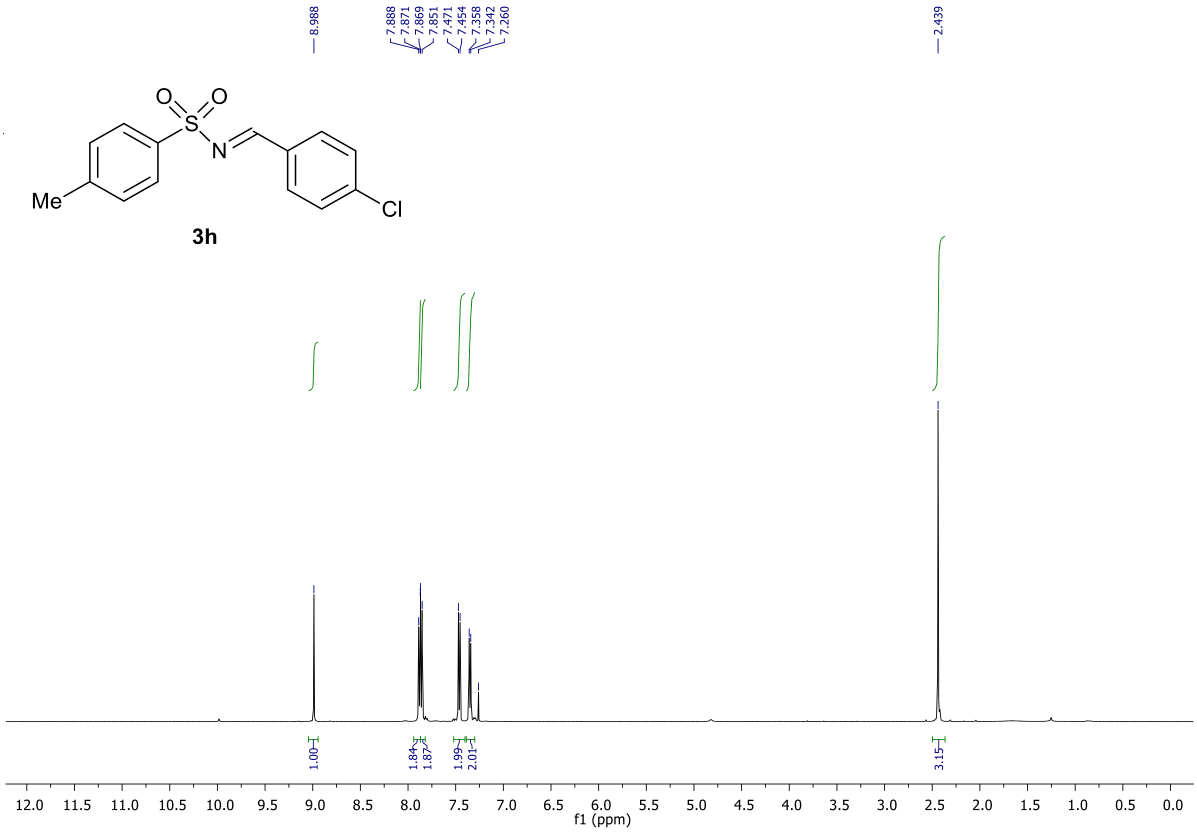
8.988
7.888
7.871
7.869
7.851
7.471
7.454
7.358
7.342
7.260
2.439
Me
Cl
3h
1.00
1.84
1.87
1.99
2.01
3.15
f1 (ppm)

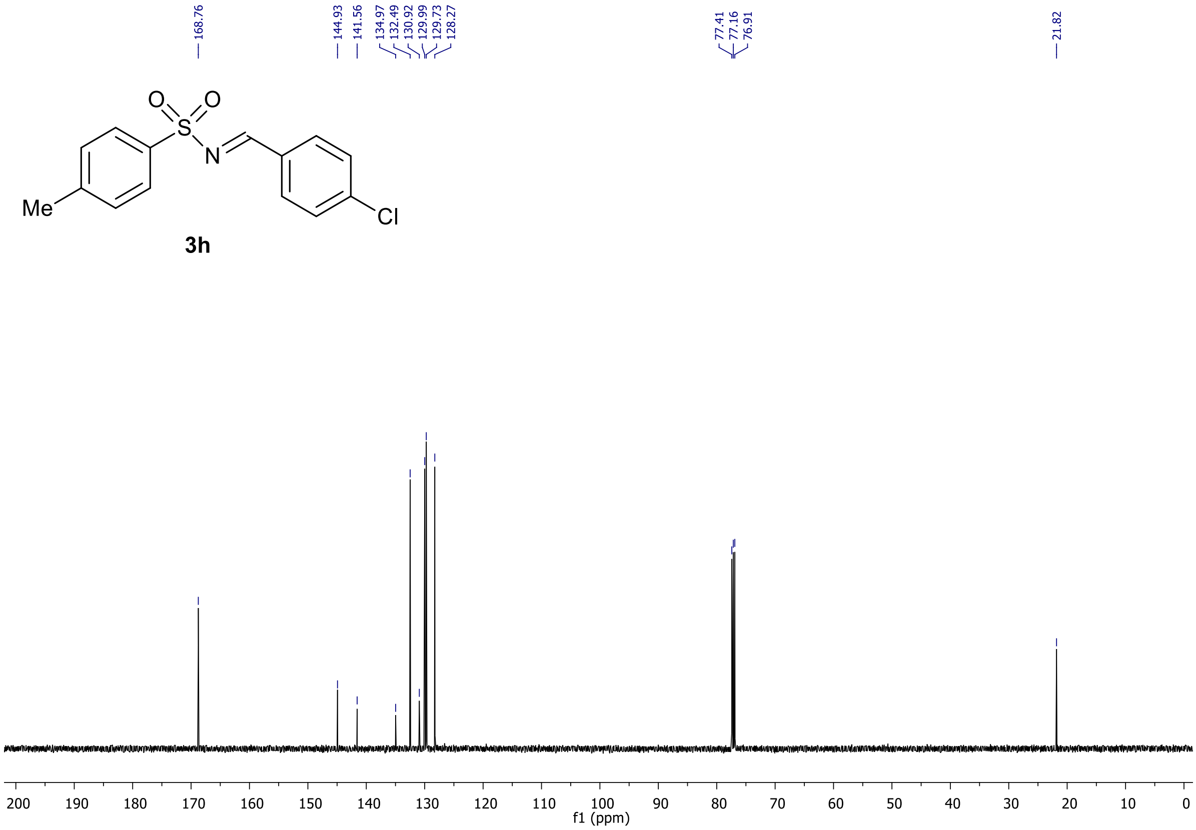
168.76
144.93
141.56
134.97
132.49
130.92
129.99
129.73
128.27
77.41
77.16
76.91
21.82
Me
Cl
3h
f1 (ppm)

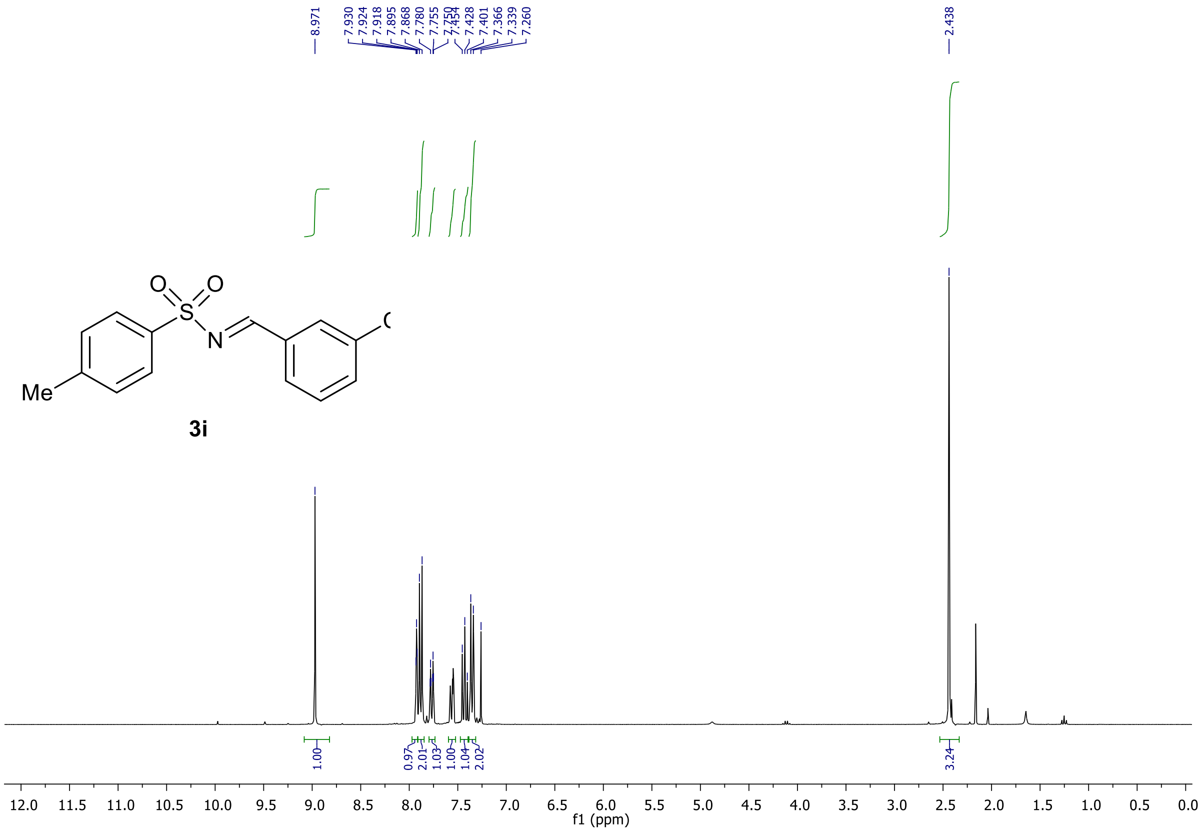
8.971
7.930 7.924 7.918 7.895 7.868 7.780 7.755 7.750 7.454 7.428 7.401 7.366 7.339 7.260
2.438
1.00 0.97 2.01 1.03 1.00 1.04 2.02 3.24
3i
f1 (ppm)

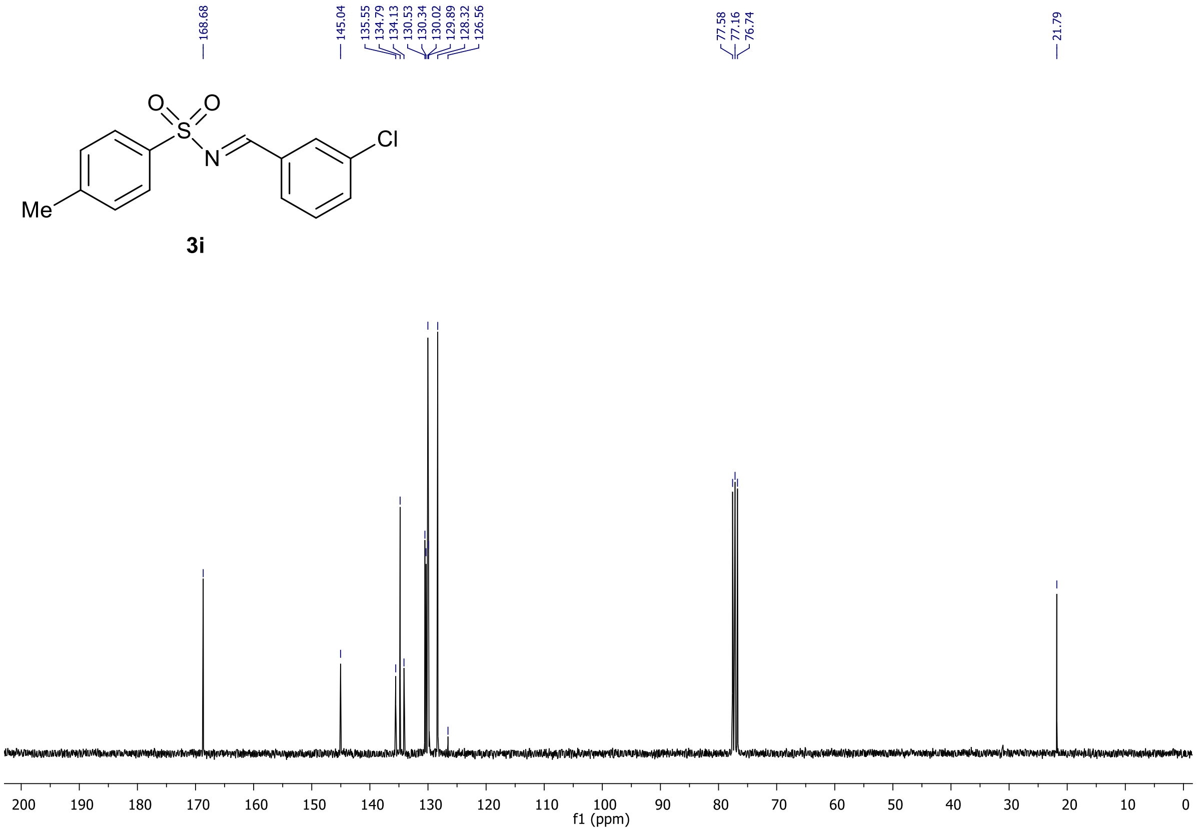
168.68
145.04 135.55 134.79 134.13 130.53 130.34 130.02 129.89 128.32 126.56
77.58 77.16 76.74
21.79
Cl
3i
f1 (ppm)

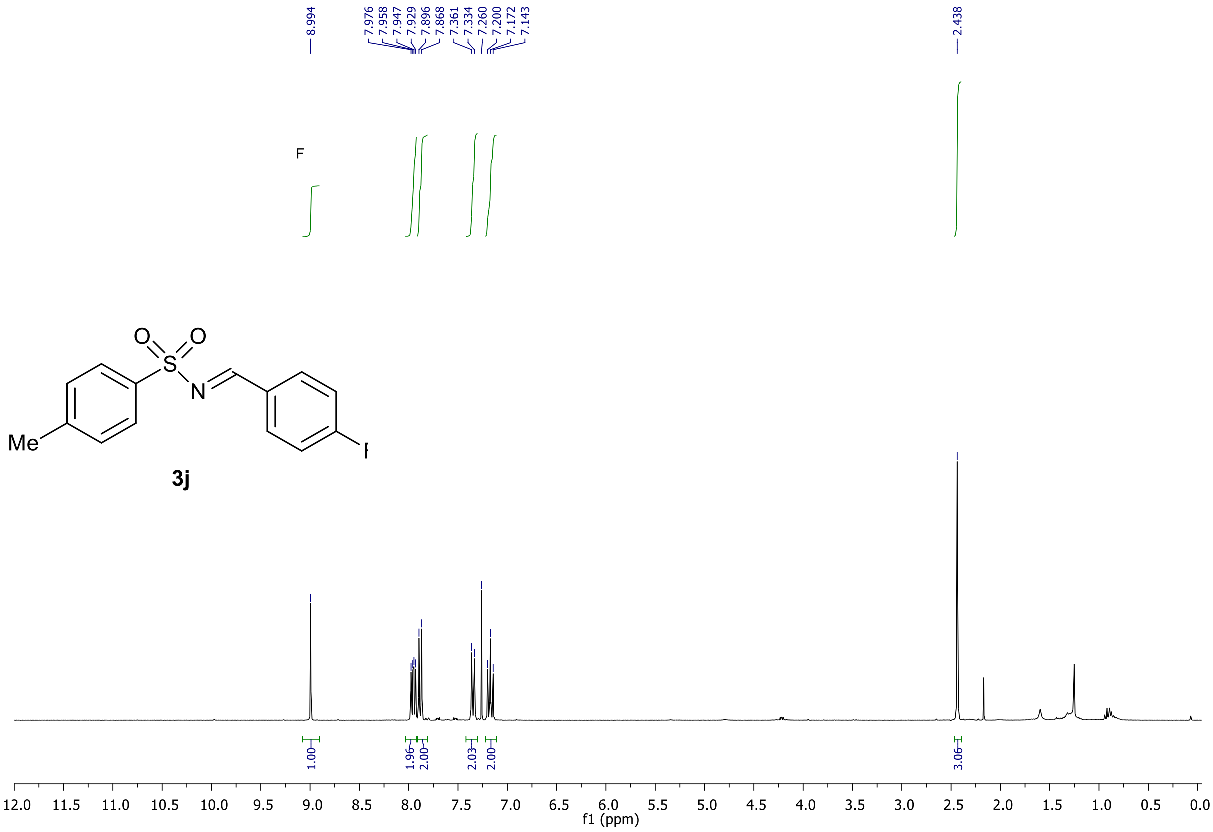
8.994
7.976 7.958 7.947 7.929 7.896 7.868 7.361 7.334 7.260 7.200 7.172 7.143
2.438
F
Me
N
S
O O
3j
1.00 1.96 2.00 2.03 2.00 3.06
12.0 11.5 11.0 10.5 10.0 9.5 9.0 8.5 8.0 7.5 7.0 6.5 6.0 5.5 5.0 4.5 4.0 3.5 3.0 2.5 2.0 1.5 1.0 0.5 0.0
f1 (ppm)

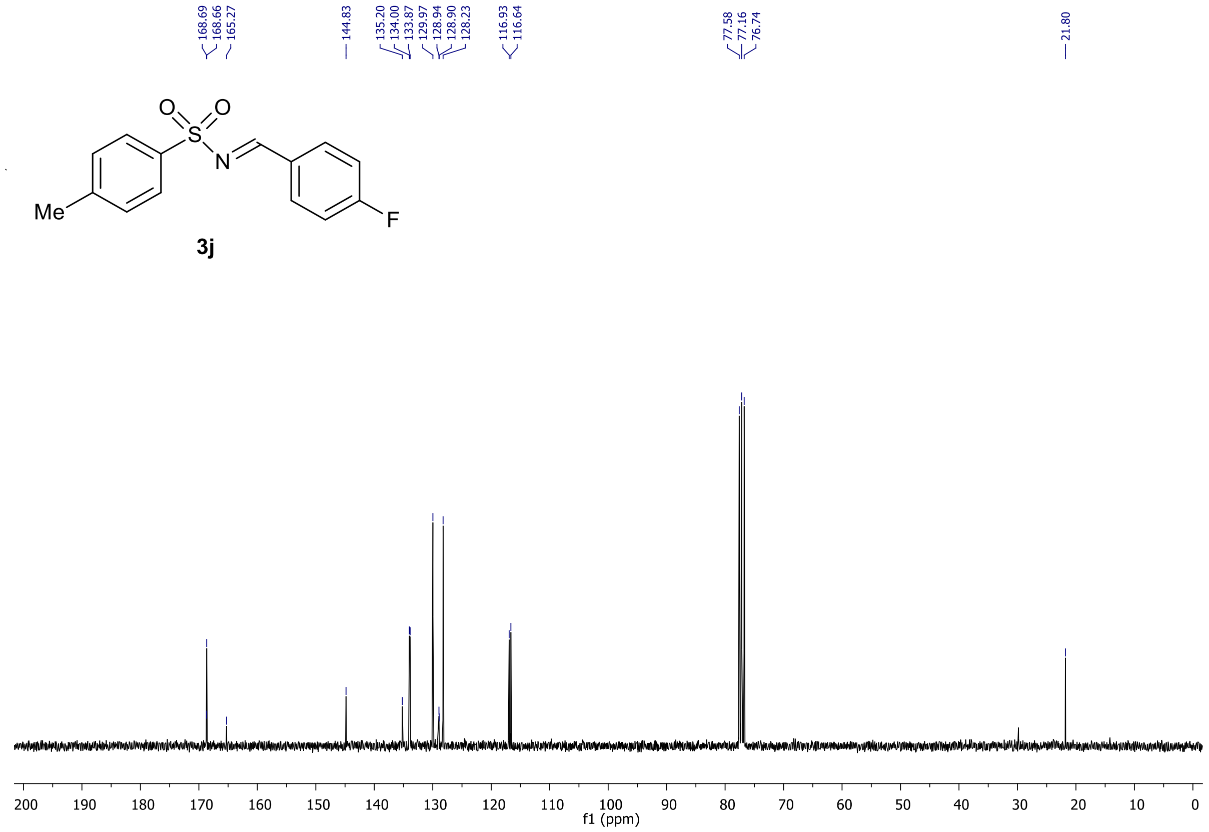
168.69 168.66 165.27
144.83
135.20 134.00 133.87 129.97 128.94 128.90 128.23
116.93 116.64
77.58 77.16 76.74
21.80
Me
N
S
O O
F
3j
200 190 180 170 160 150 140 130 120 110 100 90 80 70 60 50 40 30 20 10 0
f1 (ppm)

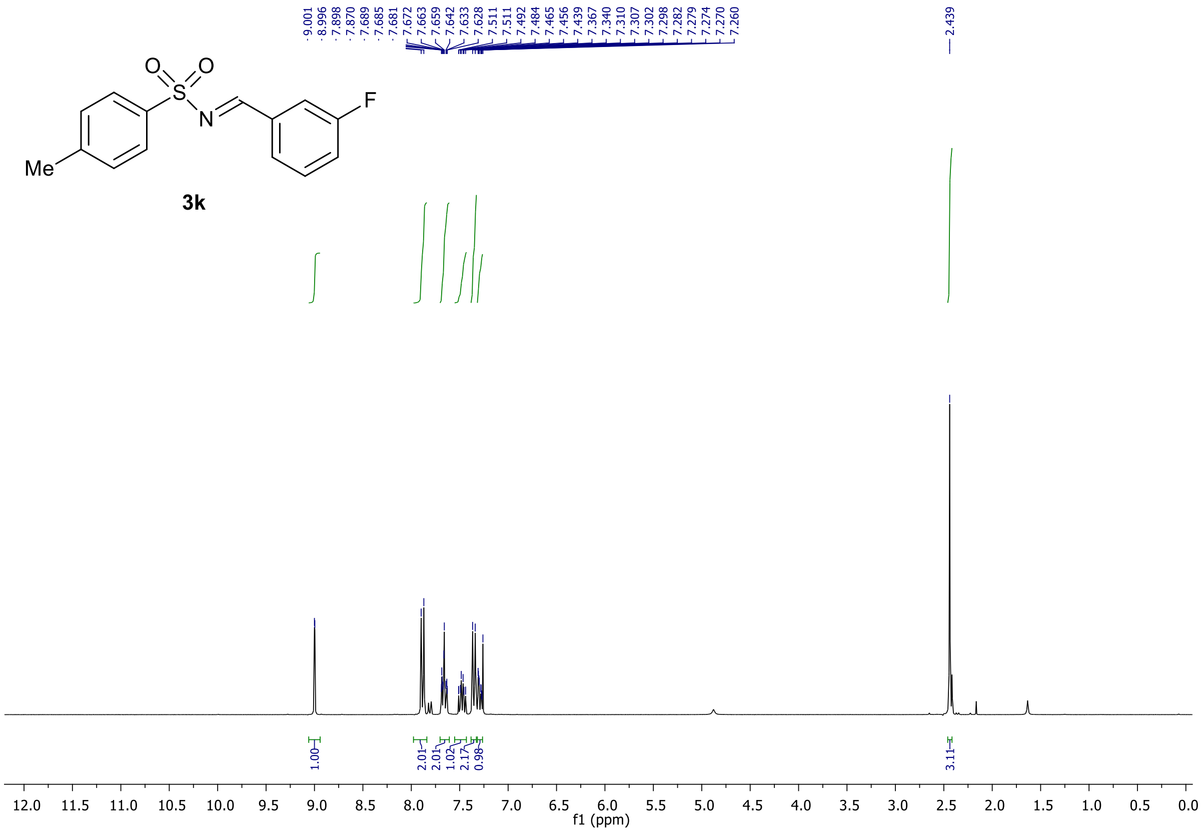

Me
3k
F
O O
S
N
9.001 8.996 7.898 7.870 7.689 7.685 7.681 7.672 7.663 7.659 7.642 7.633 7.628 7.511 7.511 7.492 7.484 7.465 7.456 7.439 7.367 7.340 7.310 7.307 7.302 7.298 7.282 7.279 7.274 7.270 7.260
2.439
1.00
2.01 2.01 1.02 2.17 0.98
3.11
12.0 11.5 11.0 10.5 10.0 9.5 9.0 8.5 8.0 7.5 7.0 6.5 6.0 5.5 5.0 4.5 4.0 3.5 3.0 2.5 2.0 1.5 1.0 0.5 0.0
f1 (ppm)

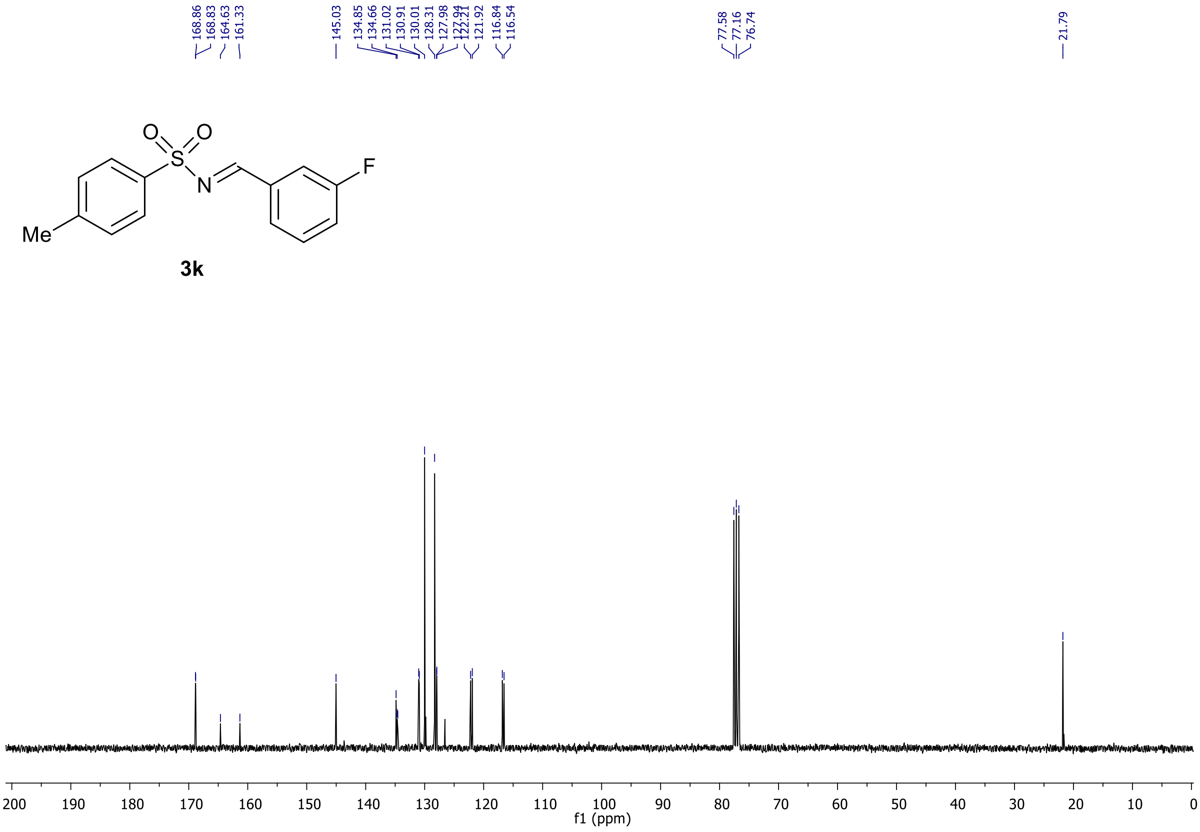

168.86 168.83 164.63 161.33
145.03 134.85 134.66 131.02 130.91 130.01 128.31 127.98 127.94 122.21 121.92 116.84 116.54
77.58 77.16 76.74
21.79
Me
3k
F
O O
S
N
200 190 180 170 160 150 140 130 120 110 100 90 80 70 60 50 40 30 20 10 0
f1 (ppm)

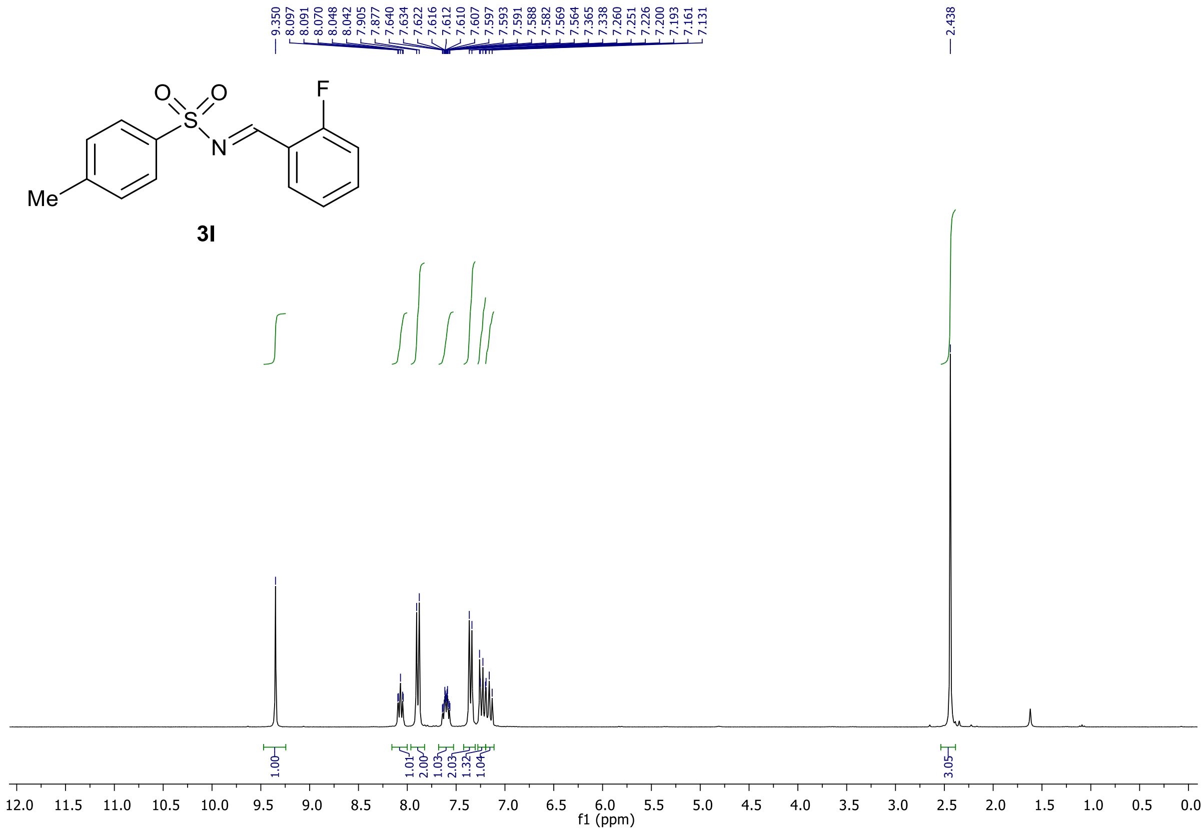
9.350 8.097 8.091 8.070 8.048 8.042 7.905 7.877 7.640 7.634 7.622 7.616 7.612 7.610 7.607 7.597 7.593 7.591 7.588 7.582 7.569 7.564 7.365 7.338 7.260 7.251 7.226 7.200 7.193 7.161 7.131
2.438
F
O
S
O
N
Me
3l
1.00
1.01
2.00
1.03
2.03
1.32
1.04
3.05
12.0 11.5 11.0 10.5 10.0 9.5 9.0 8.5 8.0 7.5 7.0 6.5 6.0 5.5 5.0 4.5 4.0 3.5 3.0 2.5 2.0 1.5 1.0 0.5 0.0
f1 (ppm)

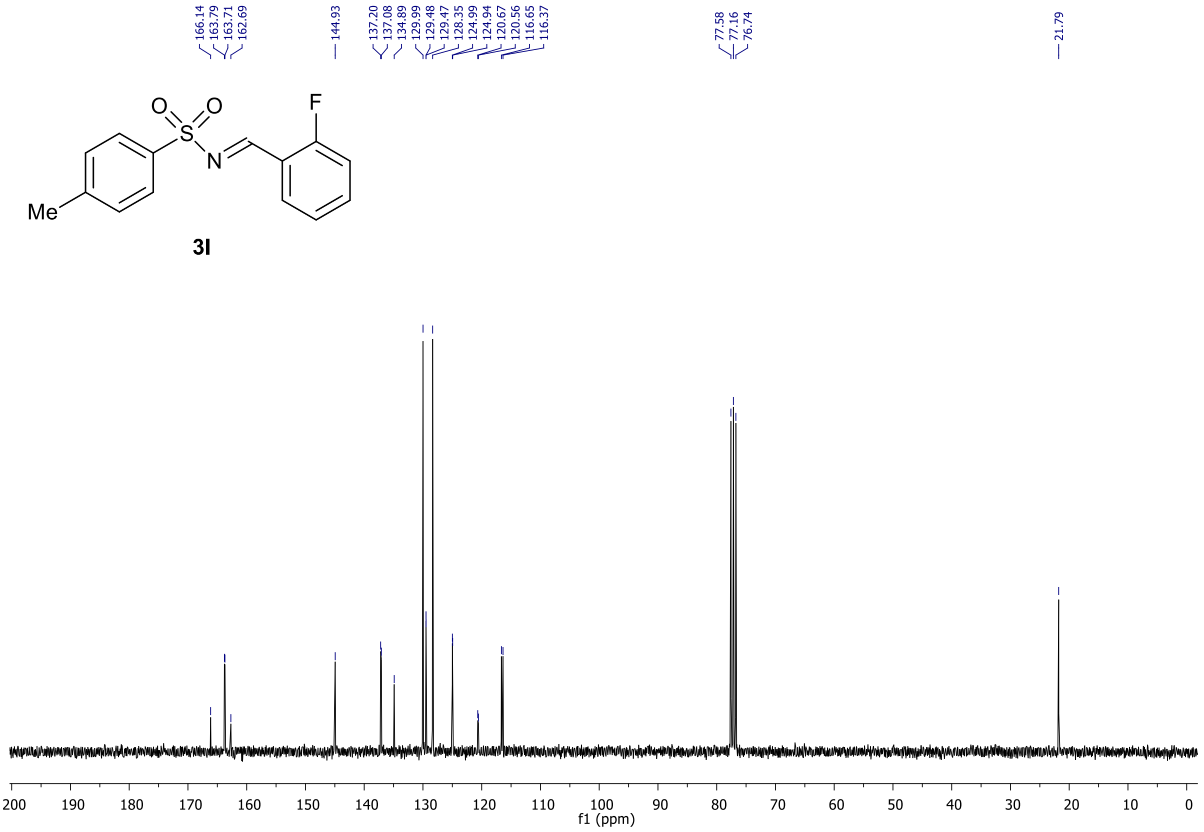
166.14 163.79 163.71 162.69
144.93
137.20 137.08 134.89 129.99 129.48 129.47 128.35 124.99 124.94 120.67 120.56 116.65 116.37
77.58 77.16 76.74
21.79
F
O
S
O
N
Me
3l
200 190 180 170 160 150 140 130 120 110 100 90 80 70 60 50 40 30 20 10 0
f1 (ppm)

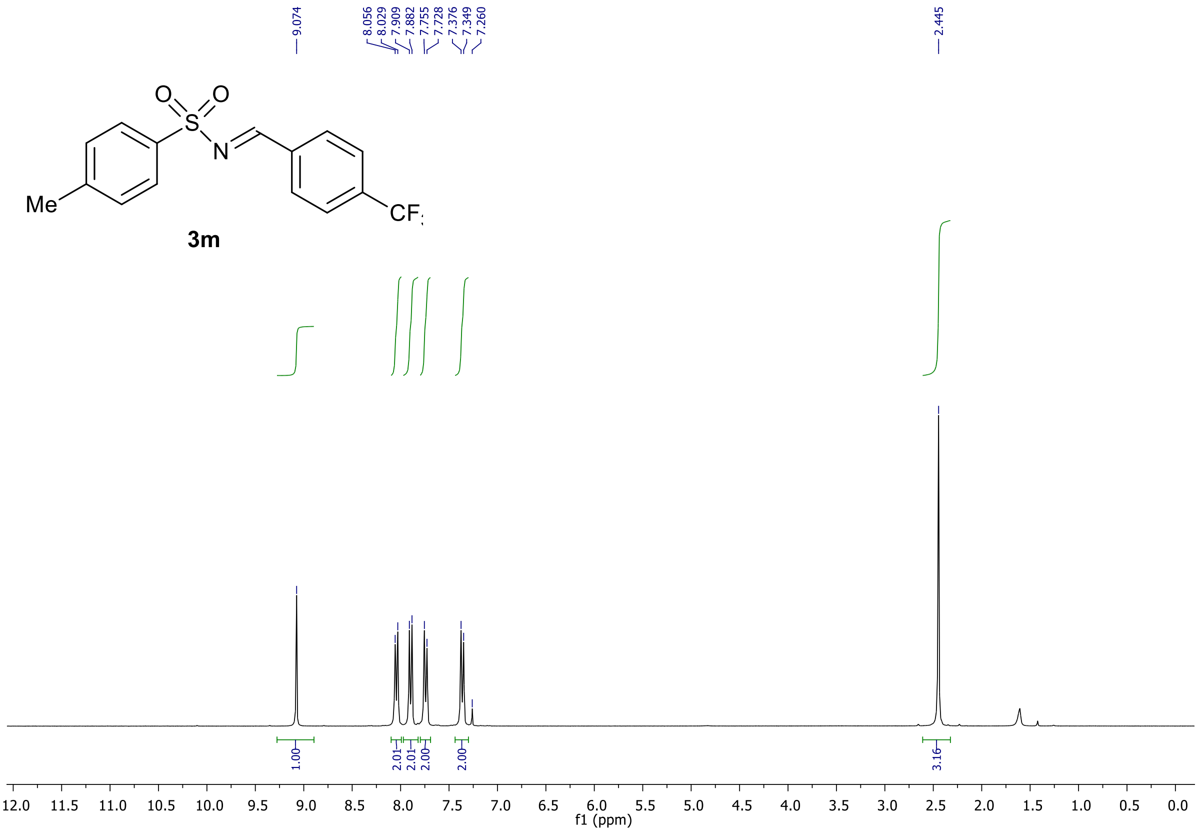

9.074
8.056
8.029
7.909
7.882
7.755
7.728
7.376
7.349
7.260
2.445
Me
3m
1.00
2.01
2.01
2.00
2.00
3.16
12.0 11.5 11.0 10.5 10.0 9.5 9.0 8.5 8.0 7.5 7.0 6.5 6.0 5.5 5.0 4.5 4.0 3.5 3.0 2.5 2.0 1.5 1.0 0.5 0.0
f1 (ppm)

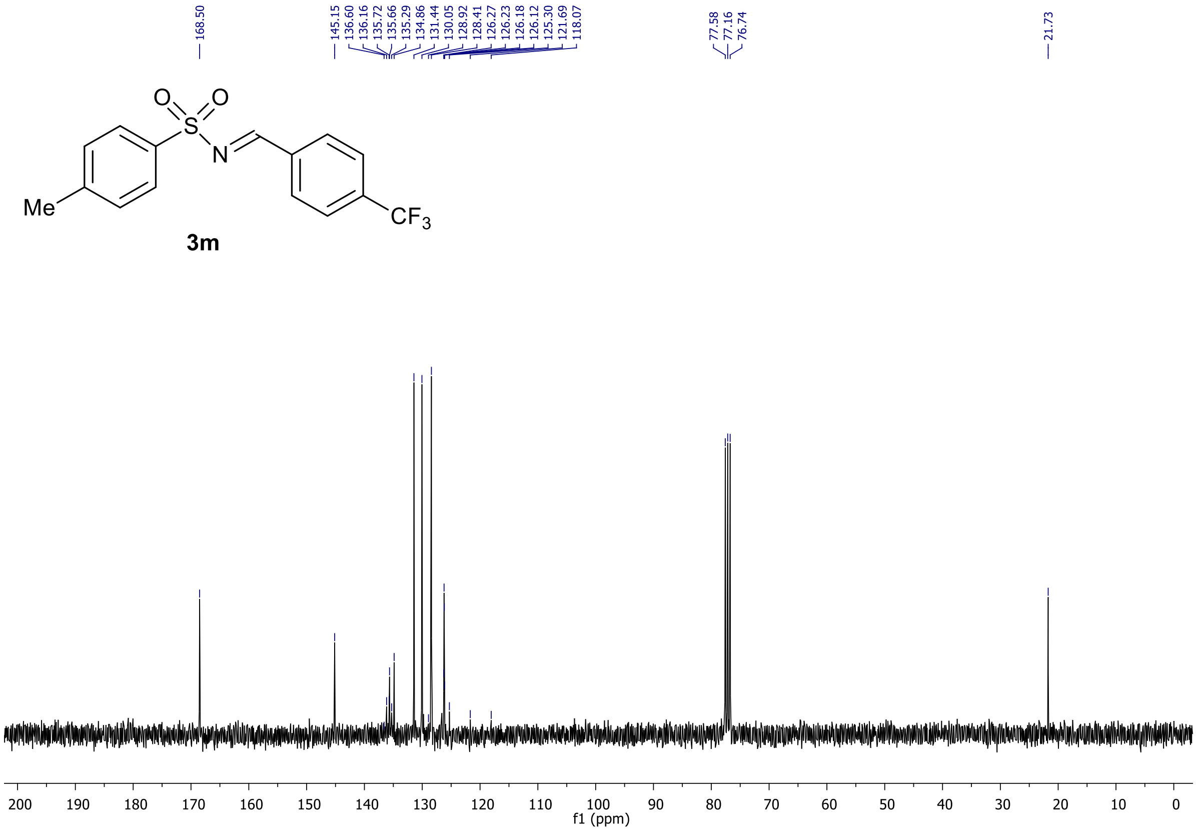

168.50
145.15
136.60
136.16
135.72
135.66
135.29
134.86
131.44
130.05
128.92
128.41
126.27
126.23
126.18
126.12
125.30
121.69
118.07
77.58
77.16
76.74
21.73
Me
CF_3
3m
200 190 180 170 160 150 140 130 120 110 100 90 80 70 60 50 40 30 20 10 0
f1 (ppm)

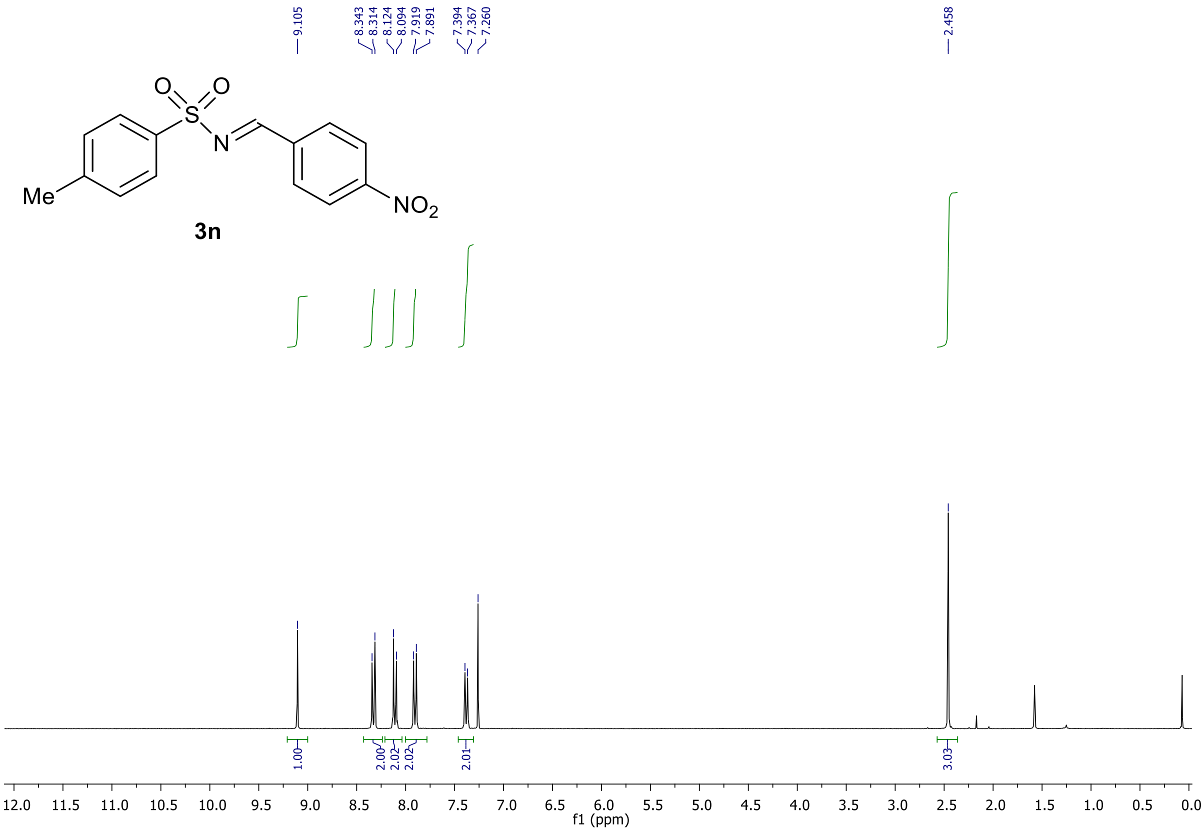
9.105
8.343
8.314
8.124
8.094
7.919
7.891
7.394
7.367
7.260
2.458
Me
NO2
1.00
2.00
2.02
2.02
2.01
3.03
12.0 11.5 11.0 10.5 10.0 9.5 9.0 8.5 8.0 7.5 7.0 6.5 6.0 5.5 5.0 4.5 4.0 3.5 3.0 2.5 2.0 1.5 1.0 0.5 0.0
f1 (ppm)

3n

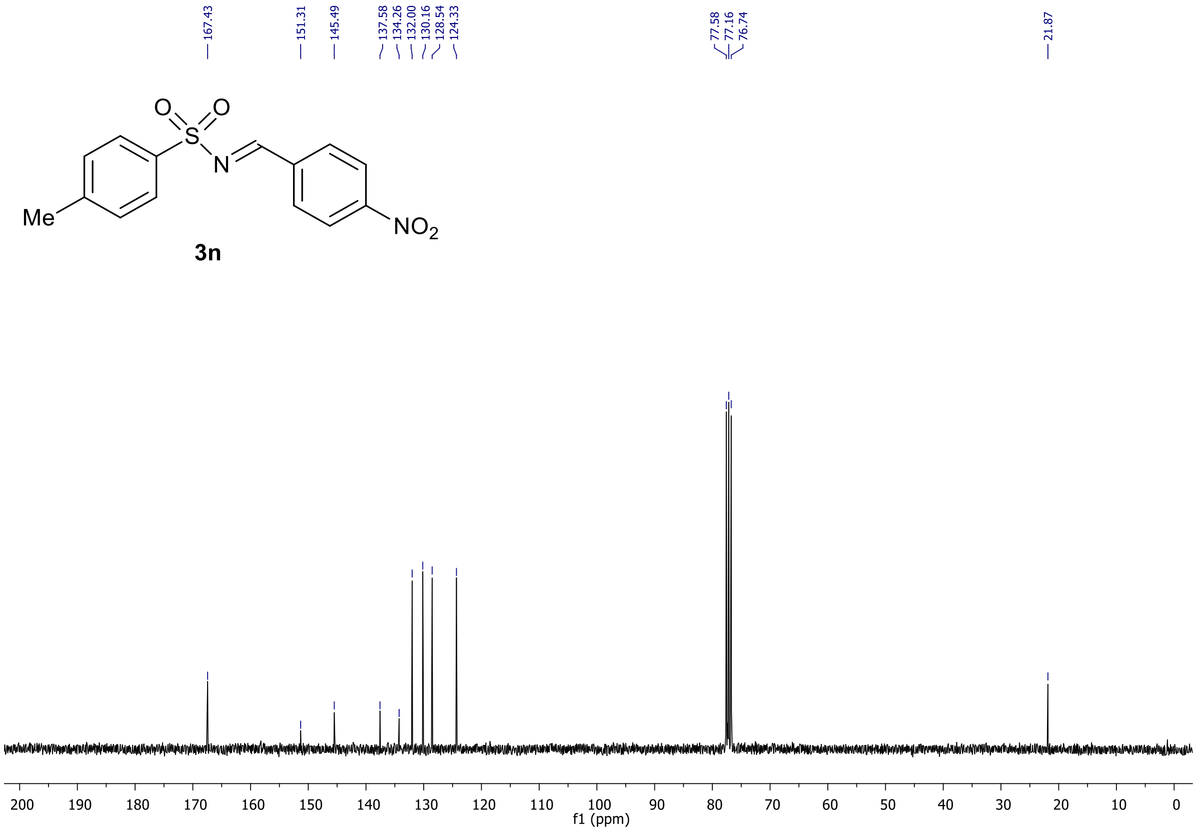
167.43
151.31
145.49
137.58
134.26
132.00
130.16
128.54
124.33
77.58
77.16
76.74
21.87
Me
NO2
200 190 180 170 160 150 140 130 120 110 100 90 80 70 60 50 40 30 20 10 0
f1 (ppm)

3n

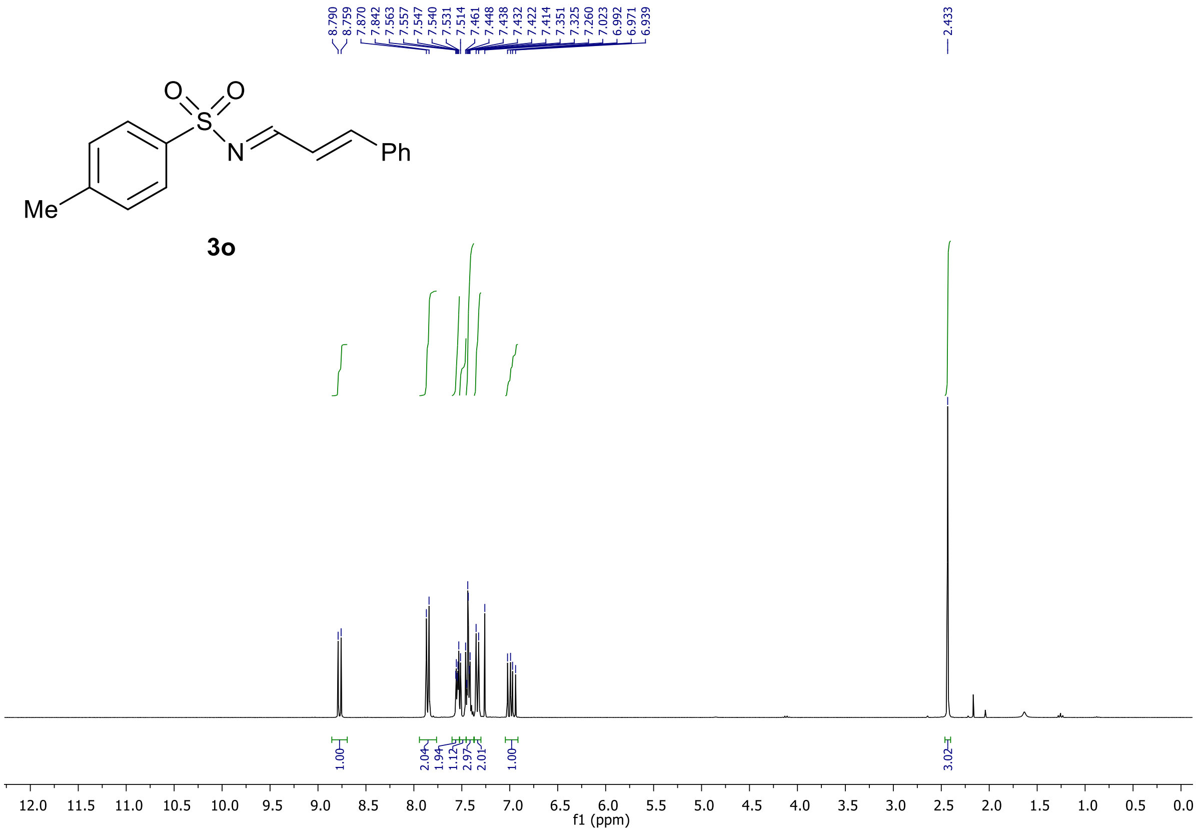
8.790
8.759
7.870
7.842
7.563
7.557
7.547
7.540
7.531
7.514
7.461
7.448
7.438
7.432
7.422
7.414
7.351
7.325
7.260
7.023
6.992
6.971
6.939
2.433
Ph
Me
3o
1.00
2.04
1.94
1.12
2.97
2.01
1.00
3.02
f1 (ppm)

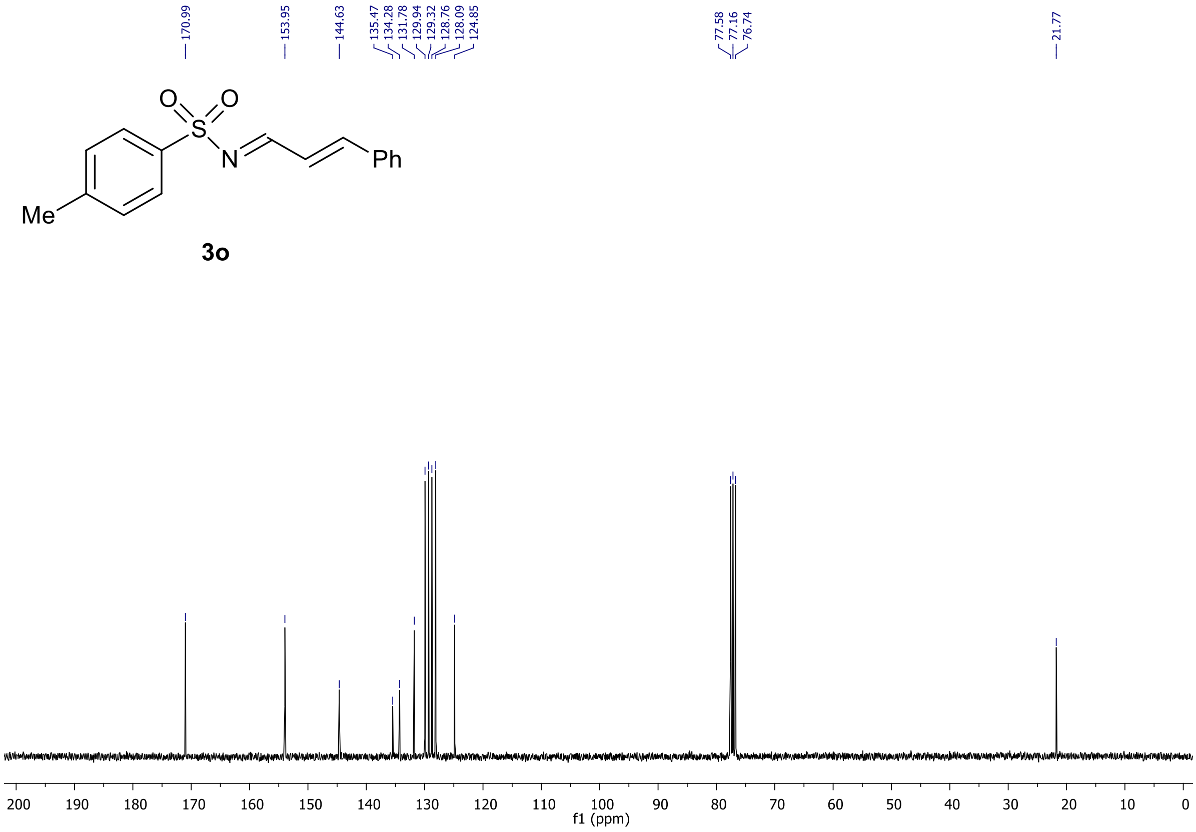
170.99
153.95
144.63
135.47
134.28
131.78
129.94
129.32
128.76
128.09
124.85
77.58
77.16
76.74
21.77
Ph
Me
3o
f1 (ppm)

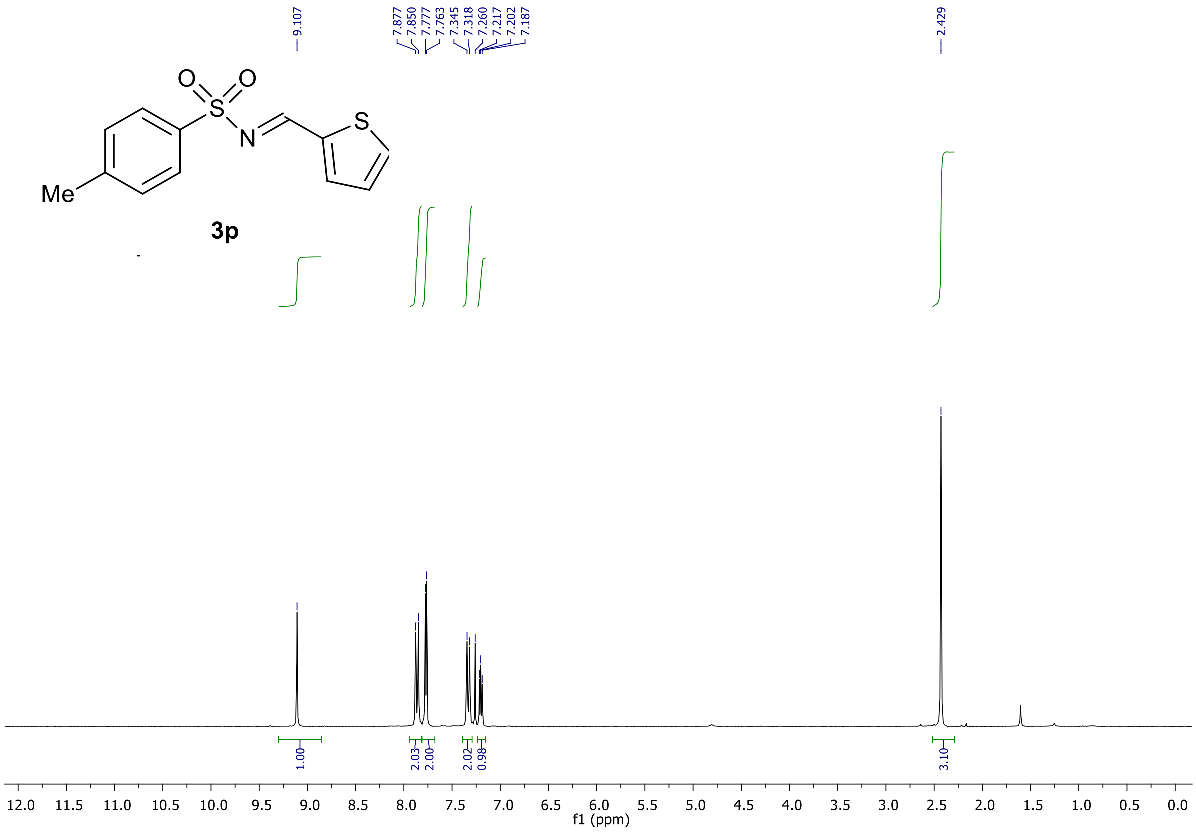
9.107
7.877
7.850
7.777
7.763
7.345
7.318
7.260
7.217
7.202
7.187
2.429
Me
S
N
O
O
3p
1.00
2.03
2.00
2.02
0.98
3.10
12.0 11.5 11.0 10.5 10.0 9.5 9.0 8.5 8.0 7.5 7.0 6.5 6.0 5.5 5.0 4.5 4.0 3.5 3.0 2.5 2.0 1.5 1.0 0.5 0.0
f1 (ppm)

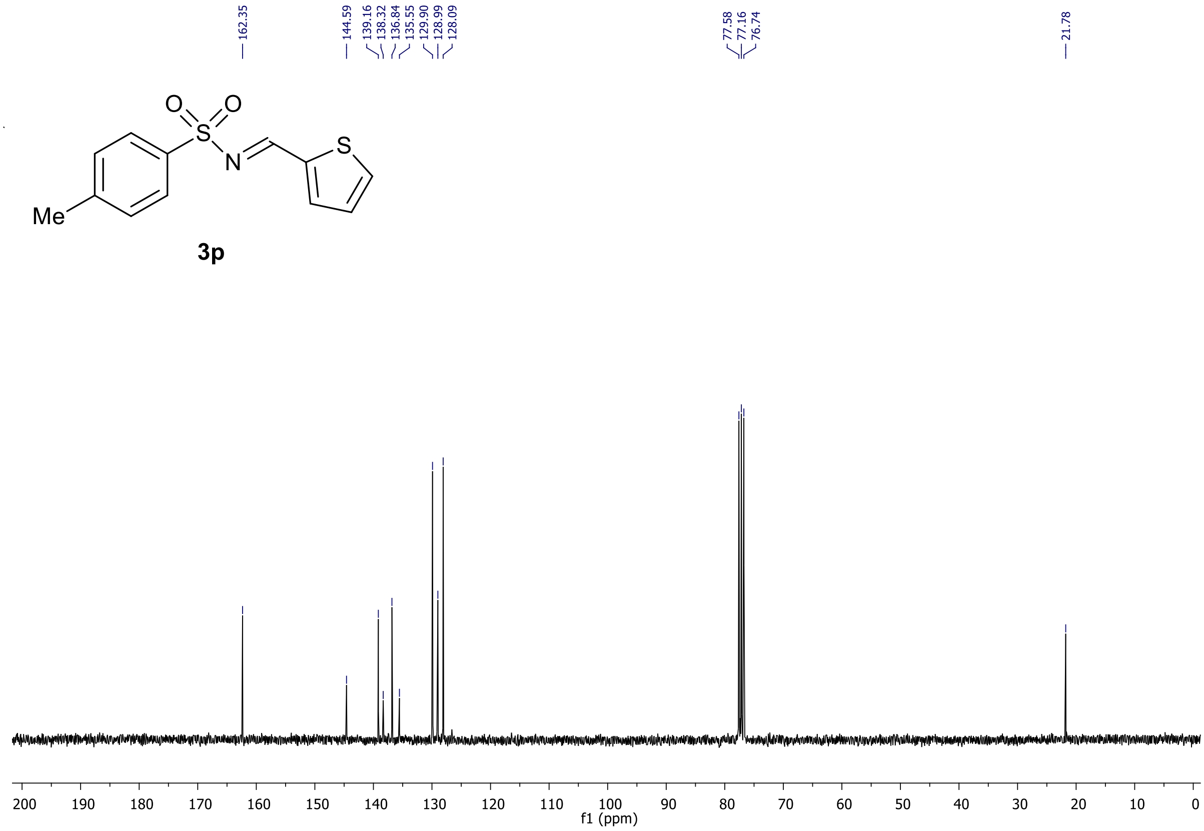
162.35
144.59
139.16
138.32
136.84
135.55
129.90
128.99
128.09
77.58
77.16
76.74
21.78
Me
S
N
O
O
3p
200 190 180 170 160 150 140 130 120 110 100 90 80 70 60 50 40 30 20 10 0
f1 (ppm)

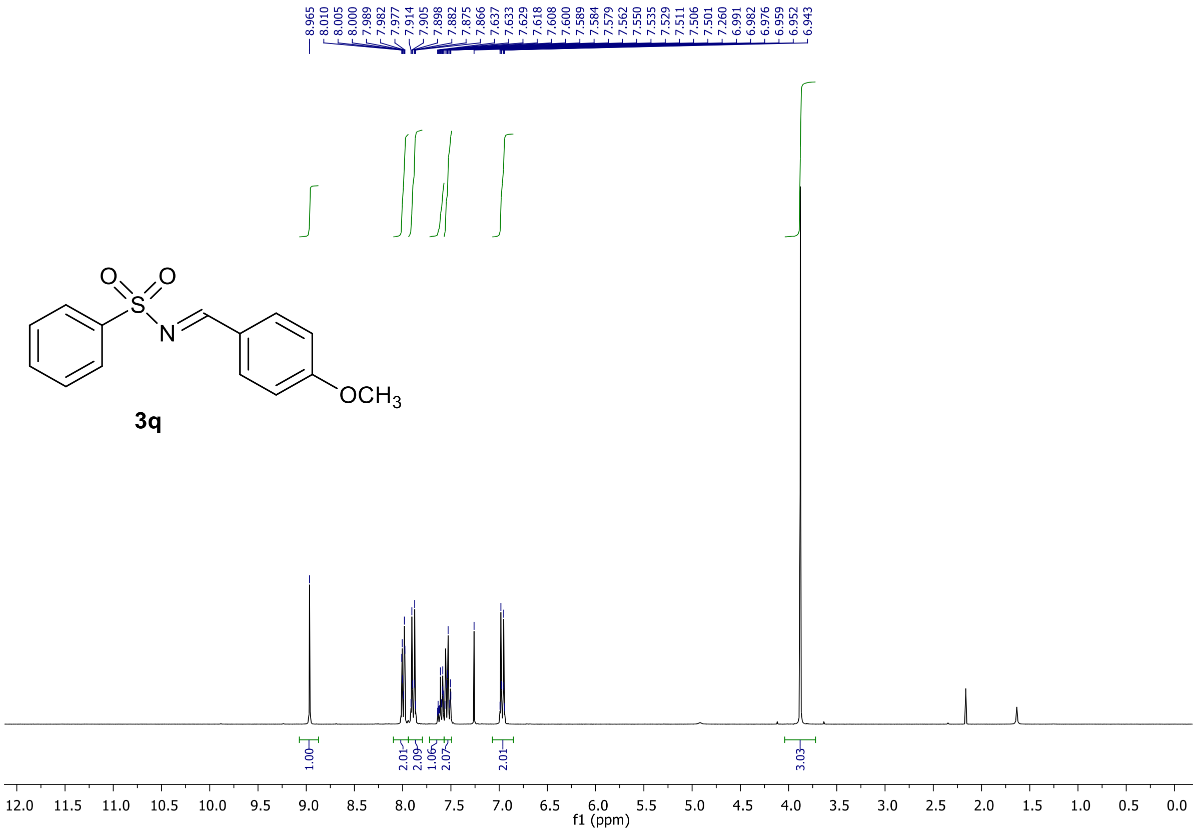
8.965 8.010 8.005 8.000 7.989 7.982 7.977 7.914 7.905 7.898 7.882 7.875 7.866 7.637 7.633 7.629 7.618 7.608 7.600 7.589 7.584 7.579 7.562 7.550 7.535 7.529 7.511 7.506 7.501 7.260 6.991 6.982 6.976 6.959 6.952 6.943
OCH3
3q
1.00
2.01
2.09
1.06
2.07
2.01
3.03
f1 (ppm)

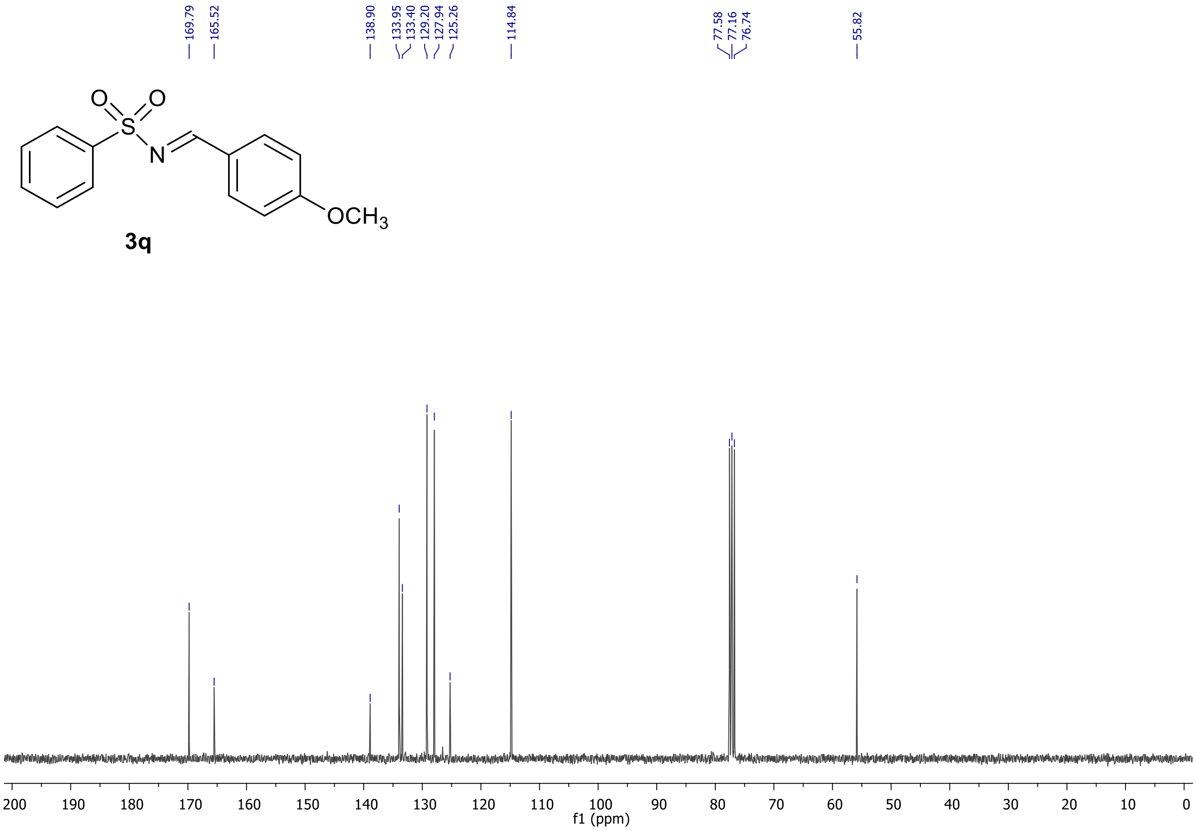
169.79 165.52 138.90 133.95 133.40 129.20 127.94 125.26 114.84 77.58 77.16 76.74 55.82
OCH3
3q
f1 (ppm)

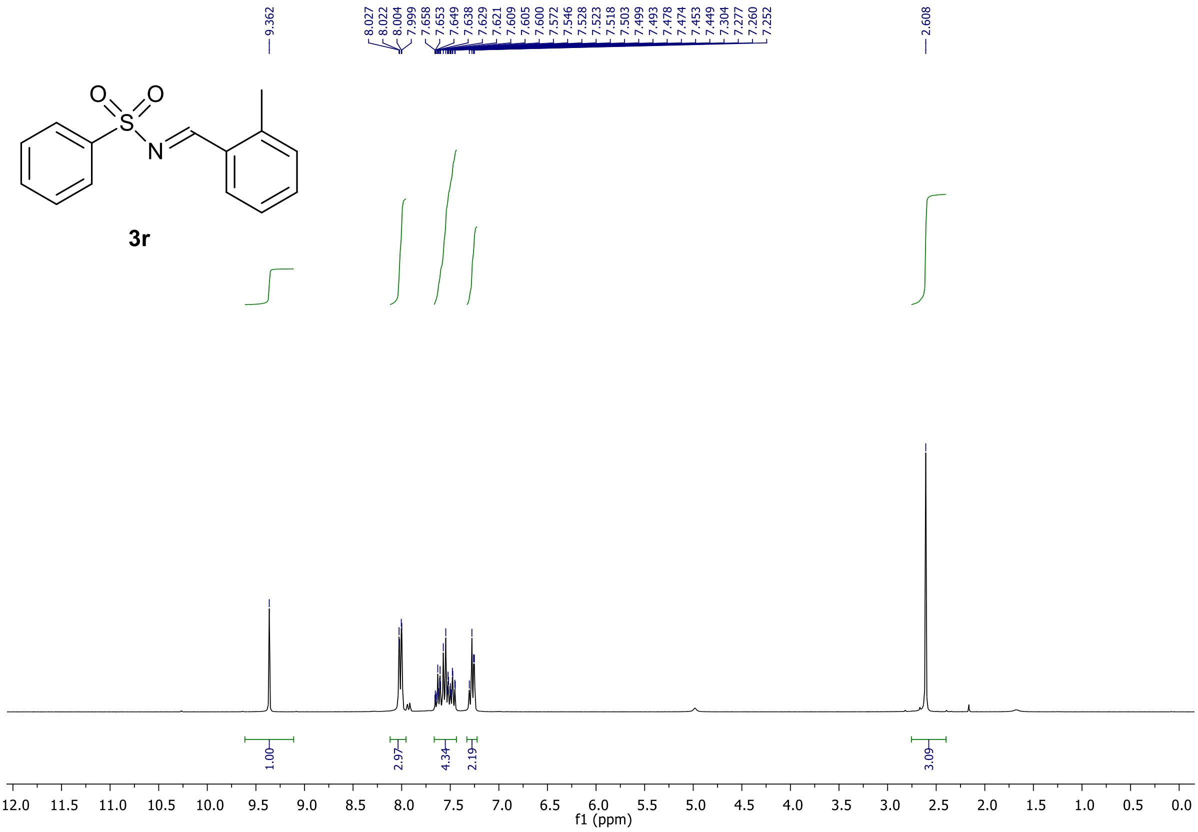
3r

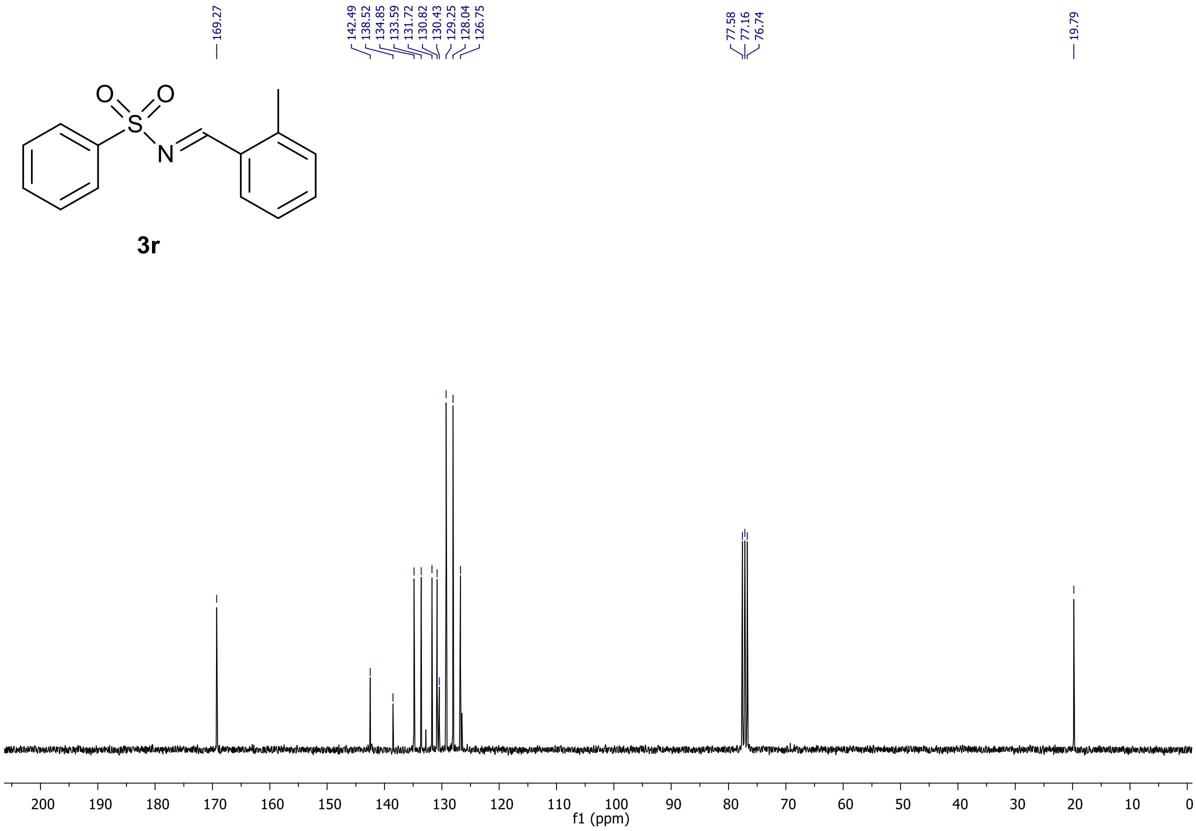
3r

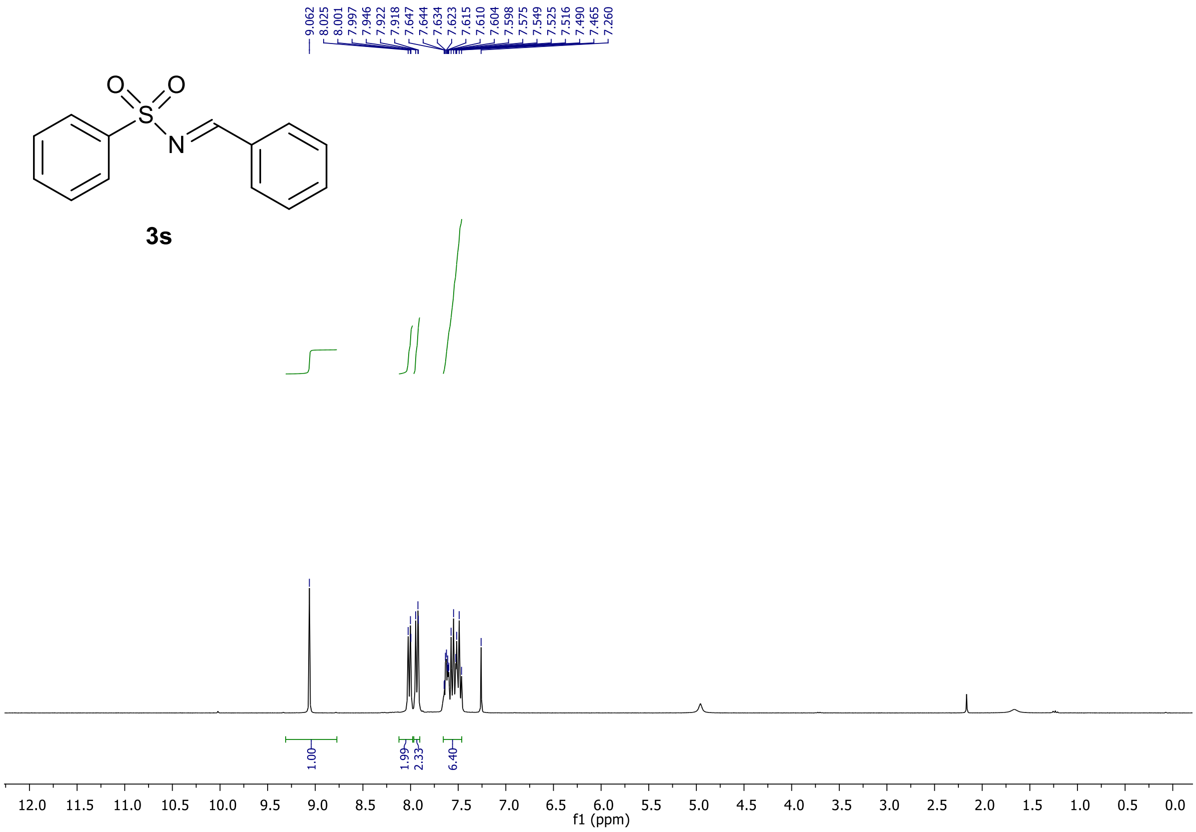
3s
9.062
8.025
8.001
7.997
7.946
7.922
7.918
7.647
7.644
7.634
7.623
7.615
7.610
7.604
7.598
7.575
7.549
7.525
7.516
7.490
7.465
7.260
1.00
1.99
2.33
6.40
12.0 11.5 11.0 10.5 10.0 9.5 9.0 8.5 8.0 7.5 7.0 6.5 6.0 5.5 5.0 4.5 4.0 3.5 3.0 2.5 2.0 1.5 1.0 0.5 0.0
f1 (ppm)

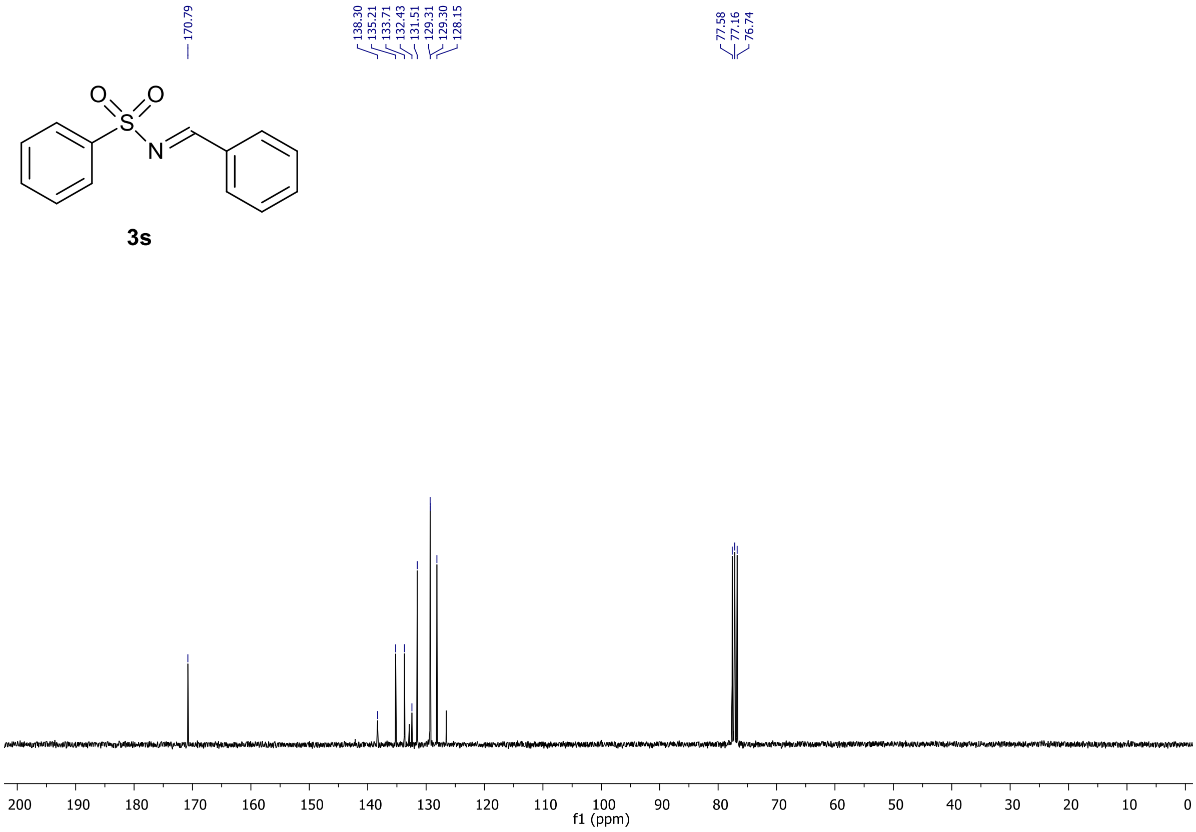
3s
170.79
138.30
135.21
133.71
132.43
131.51
129.31
129.30
128.15
77.58
77.16
76.74
200 190 180 170 160 150 140 130 120 110 100 90 80 70 60 50 40 30 20 10 0
f1 (ppm)

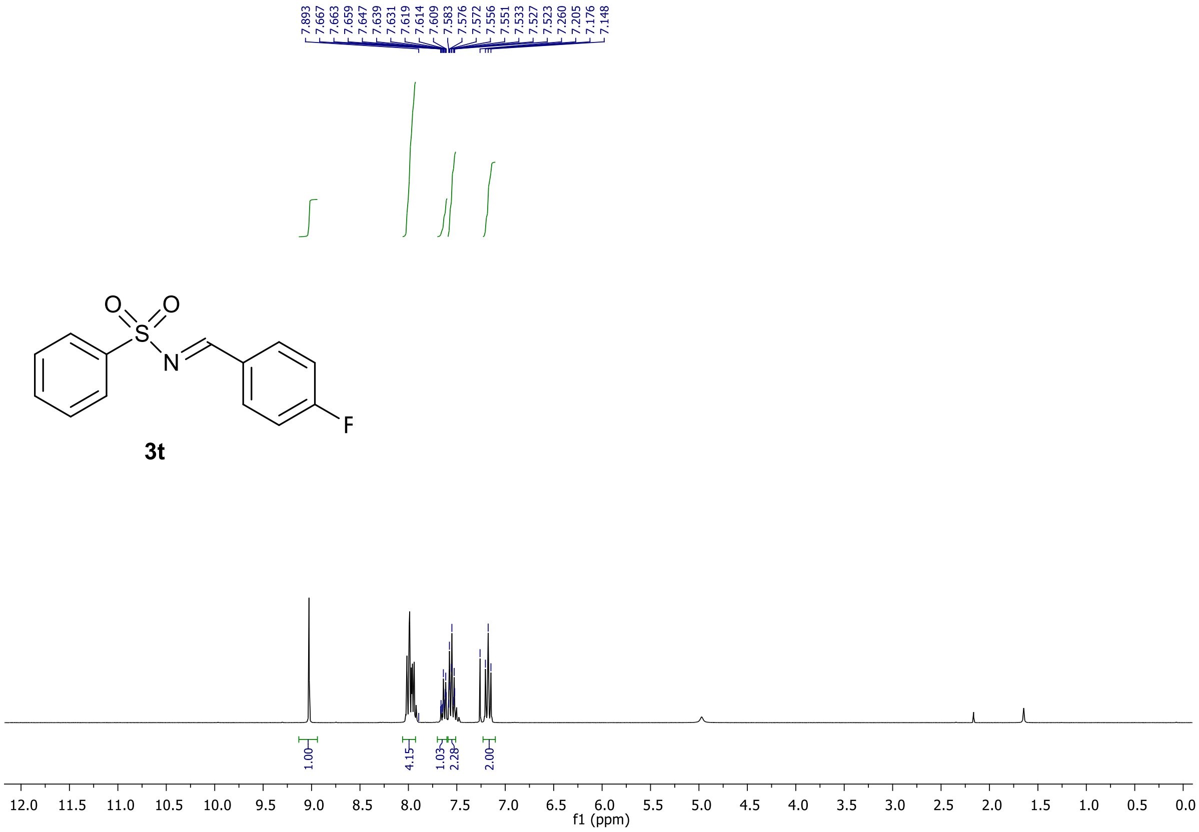
7.893
7.667
7.663
7.659
7.647
7.639
7.631
7.619
7.614
7.609
7.583
7.576
7.572
7.556
7.551
7.533
7.527
7.523
7.260
7.205
7.176
7.148
3t
1.00
4.15
1.03
2.28
2.00
f1 (ppm)

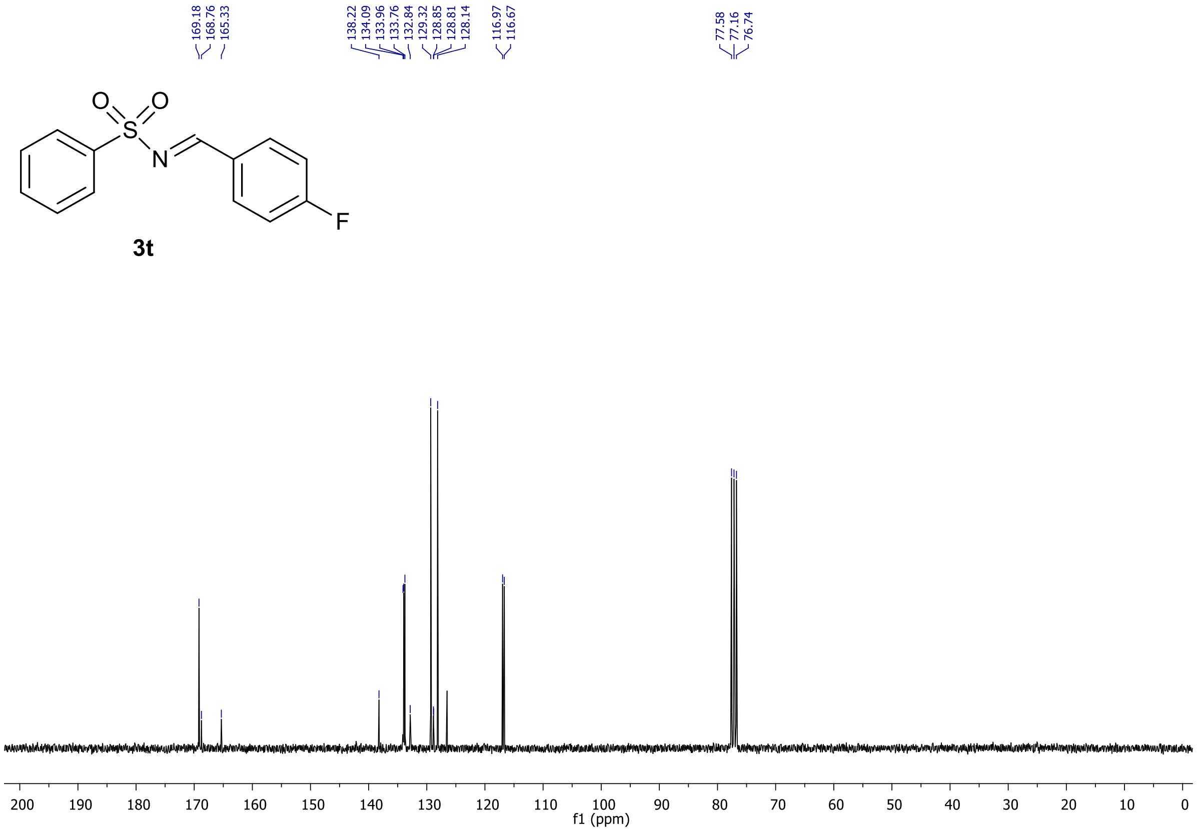
169.18
168.76
165.33
138.22
134.09
133.96
133.76
132.84
129.32
128.85
128.81
128.14
116.97
116.67
77.58
77.16
76.74
3t
f1 (ppm)

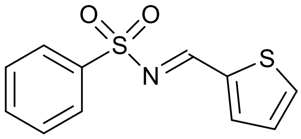

3u

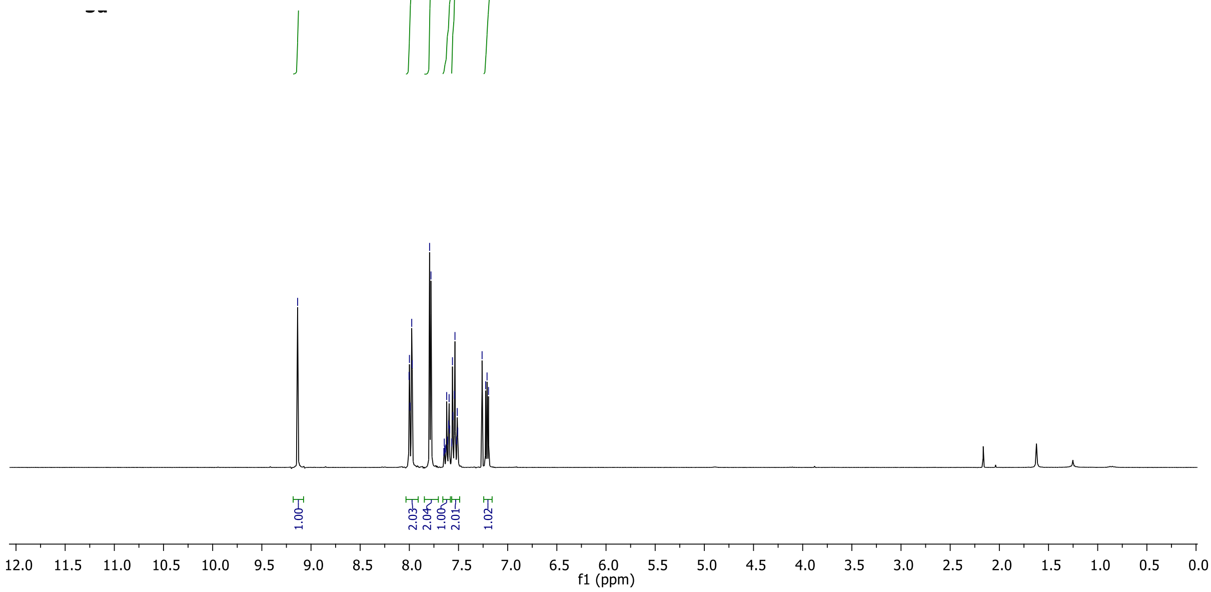
9.136
8.003
7.999
7.994
7.976
7.971
7.794
7.780
7.649
7.645
7.640
7.629
7.620
7.612
7.600
7.596
7.591
7.569
7.561
7.557
7.541
7.536
7.518
7.513
7.508
7.260
7.224
7.210
7.195
1.00
2.03
2.04
1.00
2.01
1.02
12.0 11.5 11.0 10.5 10.0 9.5 9.0 8.5 8.0 7.5 7.0 6.5 6.0 5.5 5.0 4.5 4.0 3.5 3.0 2.5 2.0 1.5 1.0 0.5 0.0
f1 (ppm)

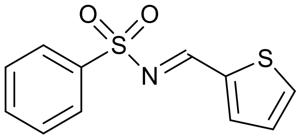

3u

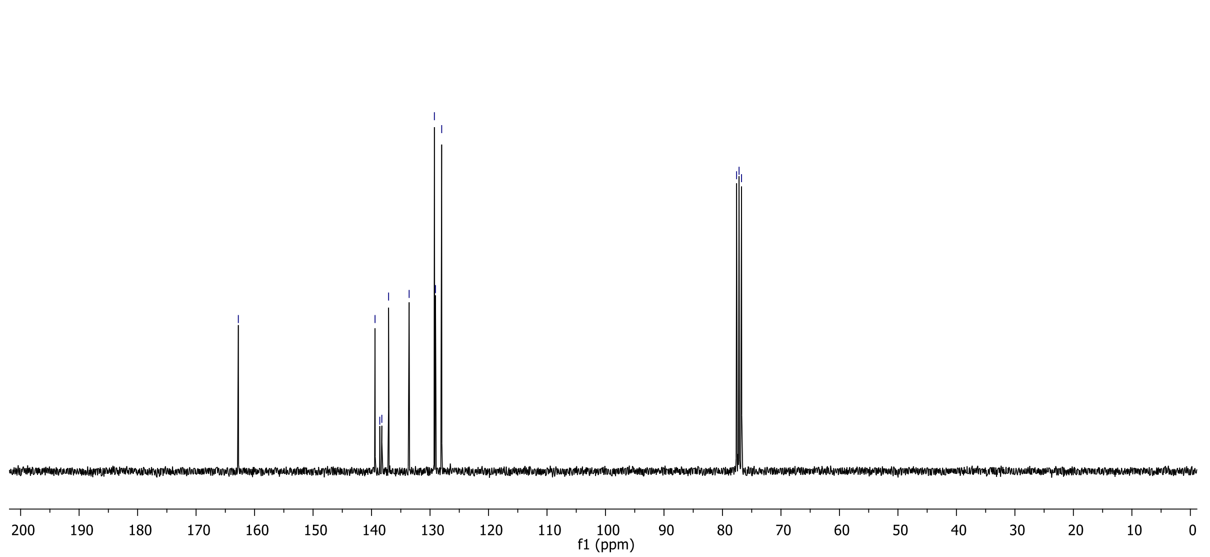
162.78
139.40
138.59
138.24
137.09
133.57
129.26
129.07
128.01
77.58
77.16
76.74
200 190 180 170 160 150 140 130 120 110 100 90 80 70 60 50 40 30 20 10 0
f1 (ppm)

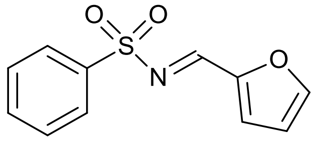
O
S
O
N
O

3v

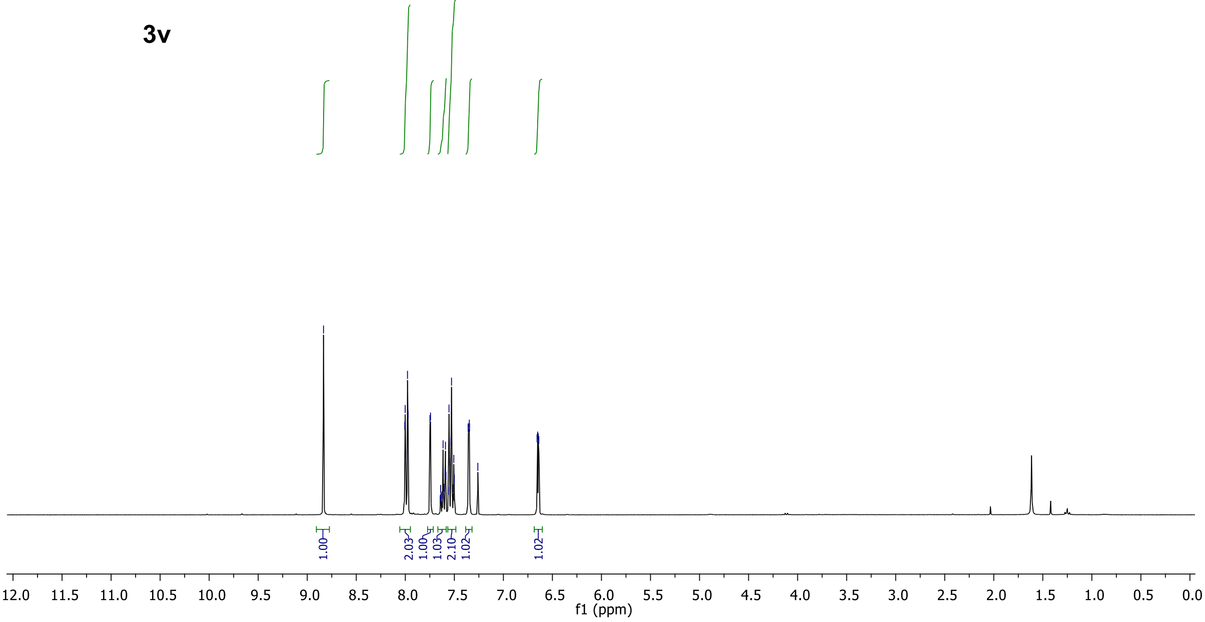
8.834
8.005
8.001
7.978
7.973
7.748
7.743
7.555
7.529
7.359
7.357
7.347
7.345
6.656
6.651
6.644
6.639
1.00
2.03
1.00
1.03
2.10
1.02
1.02
12.0 11.5 11.0 10.5 10.0 9.5 9.0 8.5 8.0 7.5 7.0 6.5 6.0 5.5 5.0 4.5 4.0 3.5 3.0 2.5 2.0 1.5 1.0 0.5 0.0
f1 (ppm)

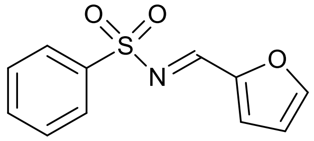
O
S
O
N
O

3v

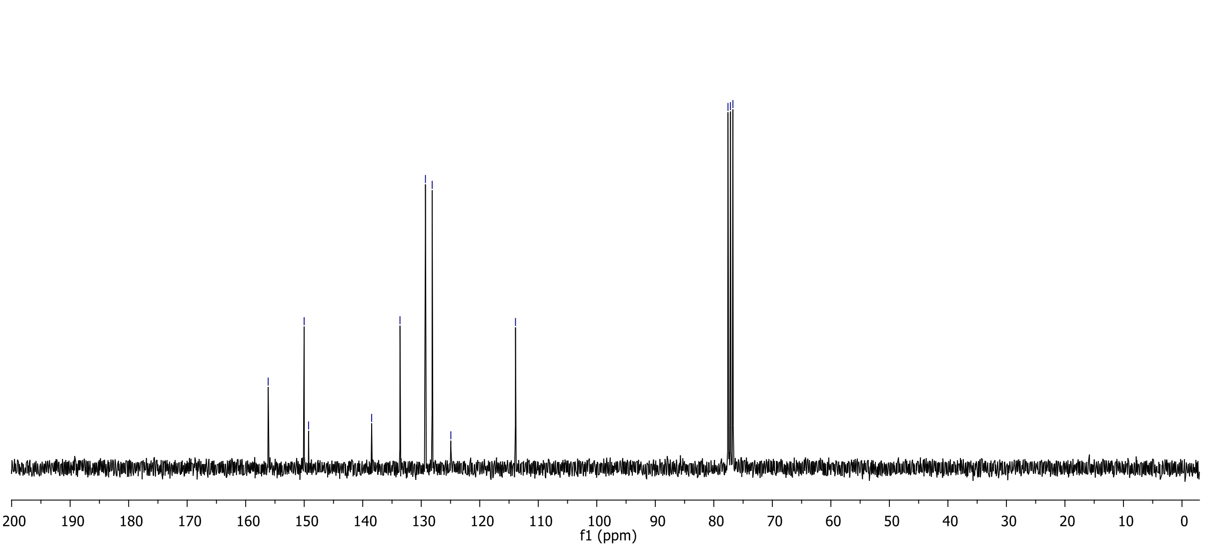
156.16
150.01
149.26
138.47
133.63
129.29
128.13
124.94
113.90
77.58
77.16
76.74
200 190 180 170 160 150 140 130 120 110 100 90 80 70 60 50 40 30 20 10 0
f1 (ppm)

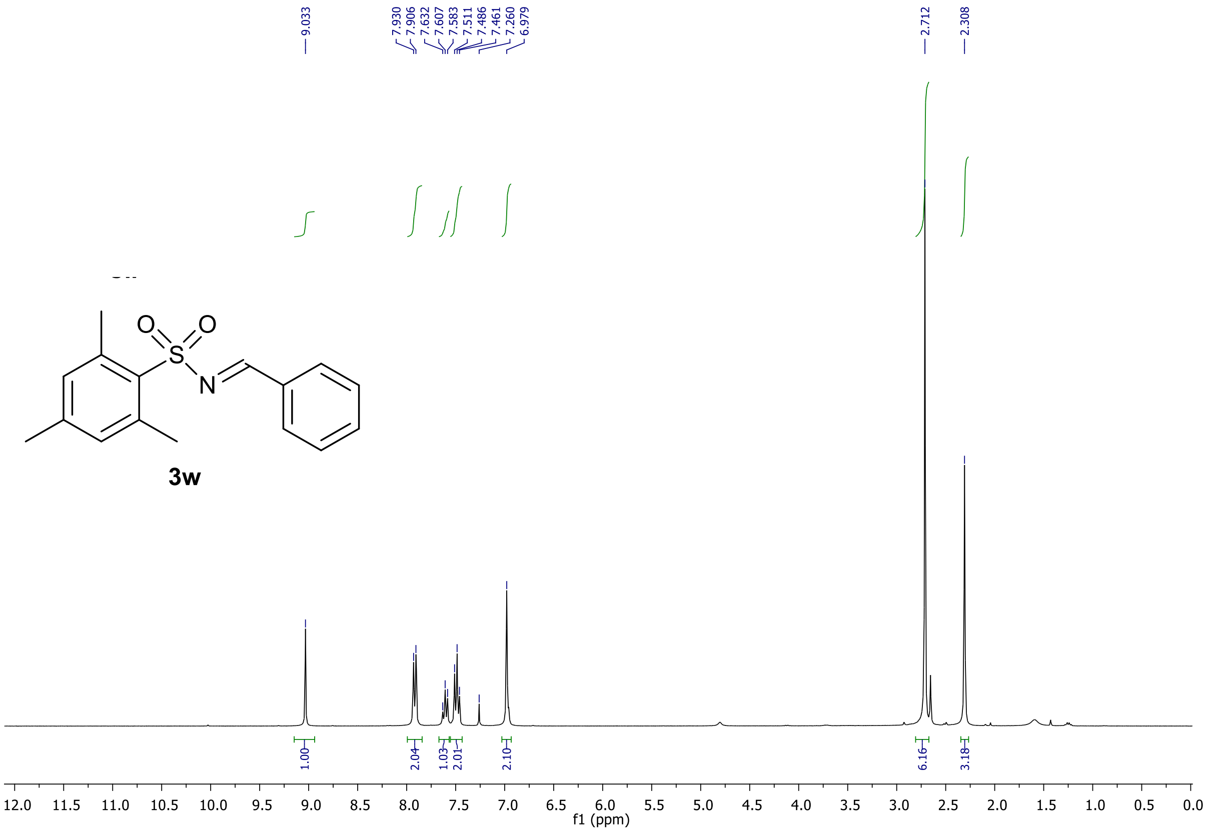
9.033
7.930
7.906
7.632
7.607
7.583
7.511
7.486
7.461
7.260
6.979
2.712
2.308
3w
1.00
2.04
1.03
2.01
2.10
6.16
3.18
12.0 11.5 11.0 10.5 10.0 9.5 9.0 8.5 8.0 7.5 7.0 6.5 6.0 5.5 5.0 4.5 4.0 3.5 3.0 2.5 2.0 1.5 1.0 0.5 0.0
f1 (ppm)

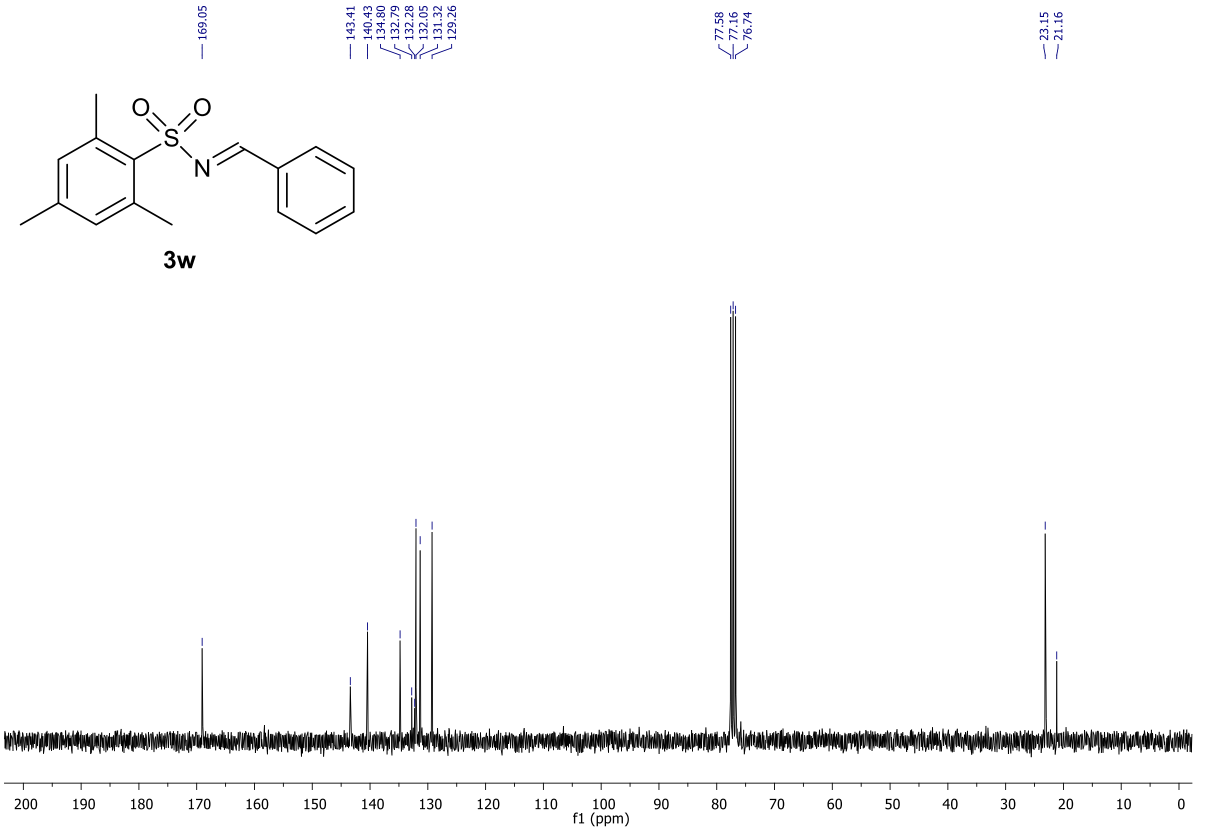
169.05
143.41
140.43
134.80
132.79
132.28
132.05
131.32
129.26
77.58
77.16
76.74
23.15
21.16
3w
200 190 180 170 160 150 140 130 120 110 100 90 80 70 60 50 40 30 20 10 0
f1 (ppm)

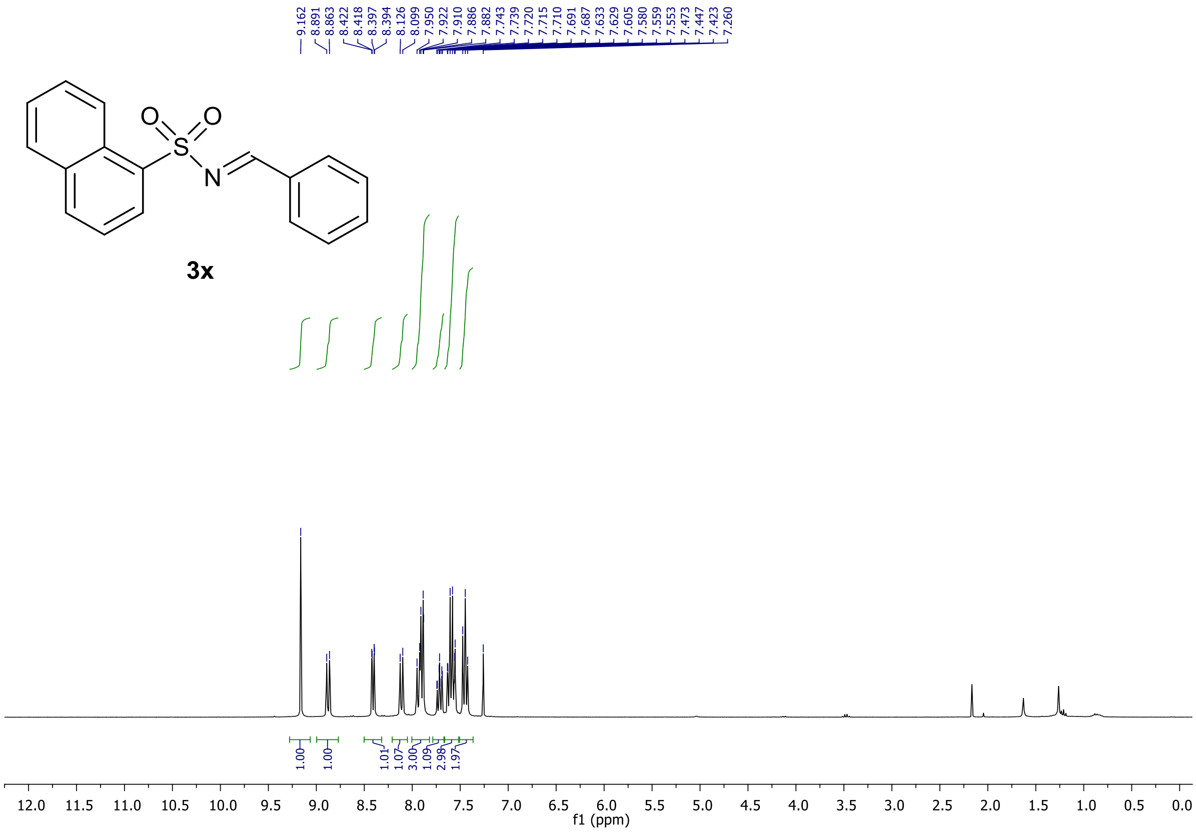

9.162
8.891
8.863
8.422
8.418
8.397
8.394
8.126
8.099
7.950
7.922
7.910
7.886
7.882
7.743
7.739
7.720
7.715
7.710
7.691
7.687
7.633
7.629
7.605
7.580
7.559
7.553
7.473
7.447
7.423
7.260
O
S
O
N
3x
1.00
1.00
1.01
1.07
3.00
1.09
2.98
1.97
12.0 11.5 11.0 10.5 10.0 9.5 9.0 8.5 8.0 7.5 7.0 6.5 6.0 5.5 5.0 4.5 4.0 3.5 3.0 2.5 2.0 1.5 1.0 0.5 0.0
f1 (ppm)

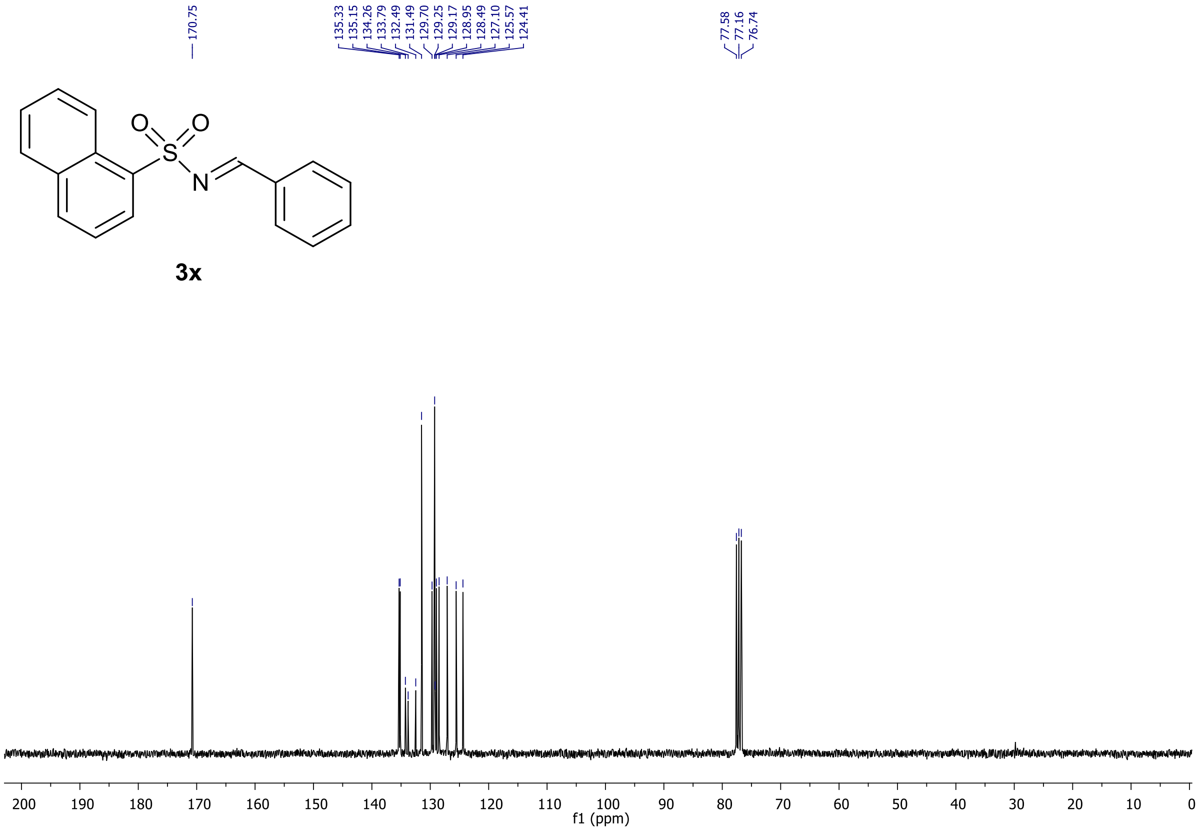

170.75
135.33
135.15
134.26
133.79
132.49
131.49
129.70
129.25
129.17
128.95
128.49
127.10
125.57
124.41
77.58
77.16
76.74
O
S
O
N
3x
200 190 180 170 160 150 140 130 120 110 100 90 80 70 60 50 40 30 20 10 0
f1 (ppm)

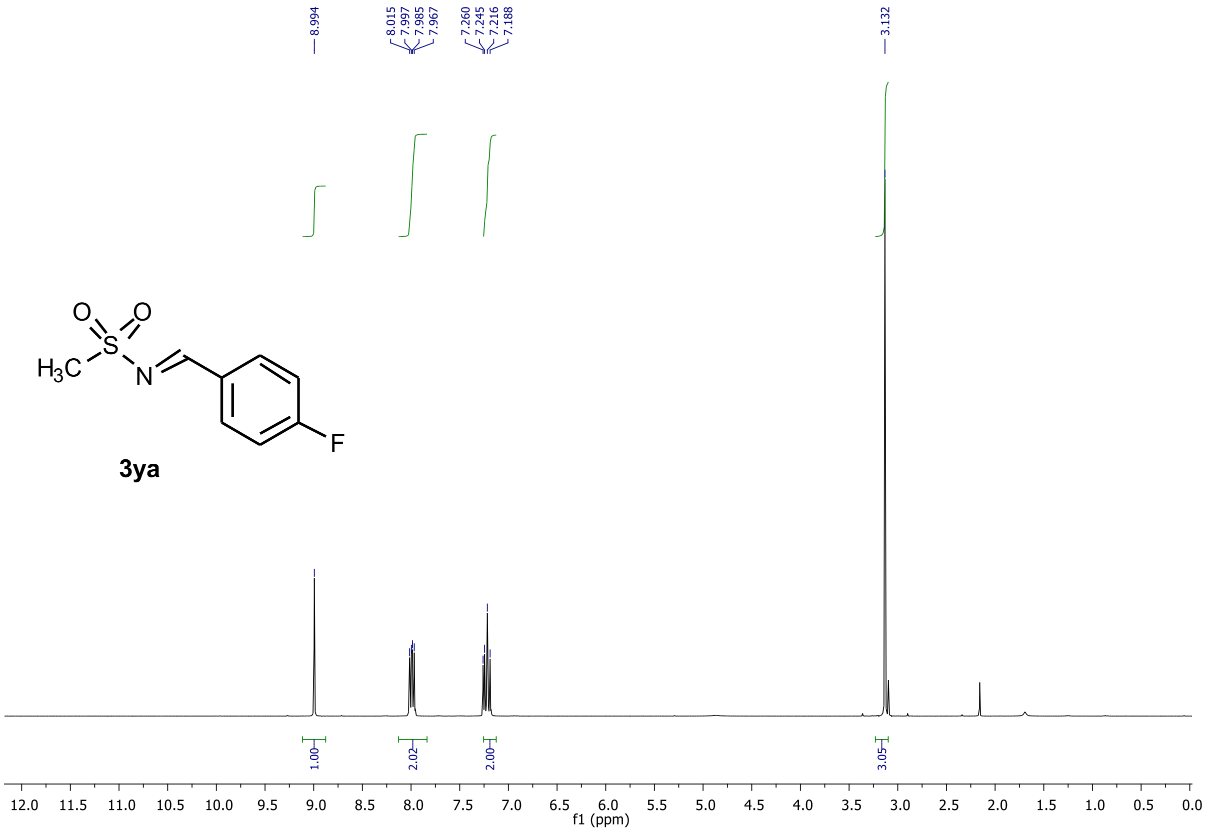

8.994
8.015
7.997
7.985
7.967
7.260
7.245
7.216
7.188
3.132
H_3C
S
O
O
N
F
3ya
1.00
2.02
2.00
3.05
12.0 11.5 11.0 10.5 10.0 9.5 9.0 8.5 8.0 7.5 7.0 6.5 6.0 5.5 5.0 4.5 4.0 3.5 3.0 2.5 2.0 1.5 1.0 0.5 0.0
f1 (ppm)

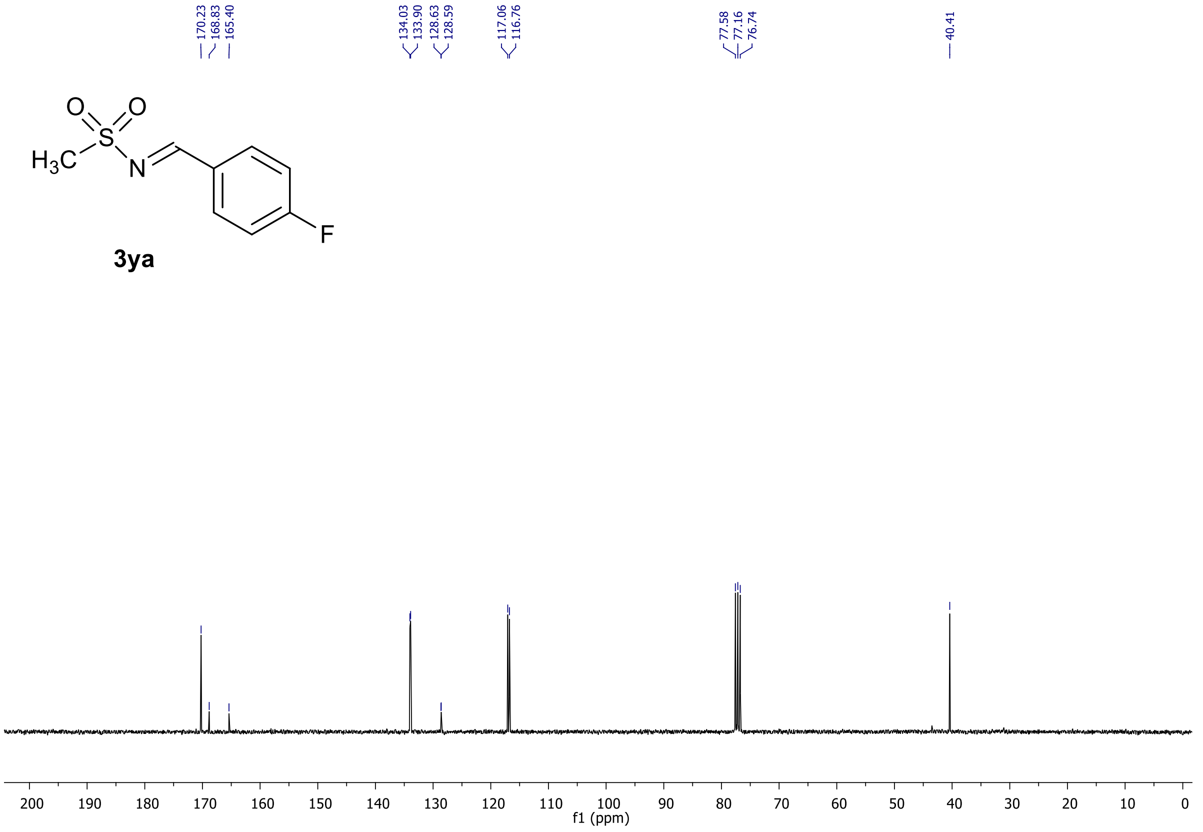

170.23
168.83
165.40
134.03
133.90
128.63
128.59
117.06
116.76
77.58
77.16
76.74
40.41
H_3C
S
O
O
N
F
3ya
200 190 180 170 160 150 140 130 120 110 100 90 80 70 60 50 40 30 20 10 0
f1 (ppm)

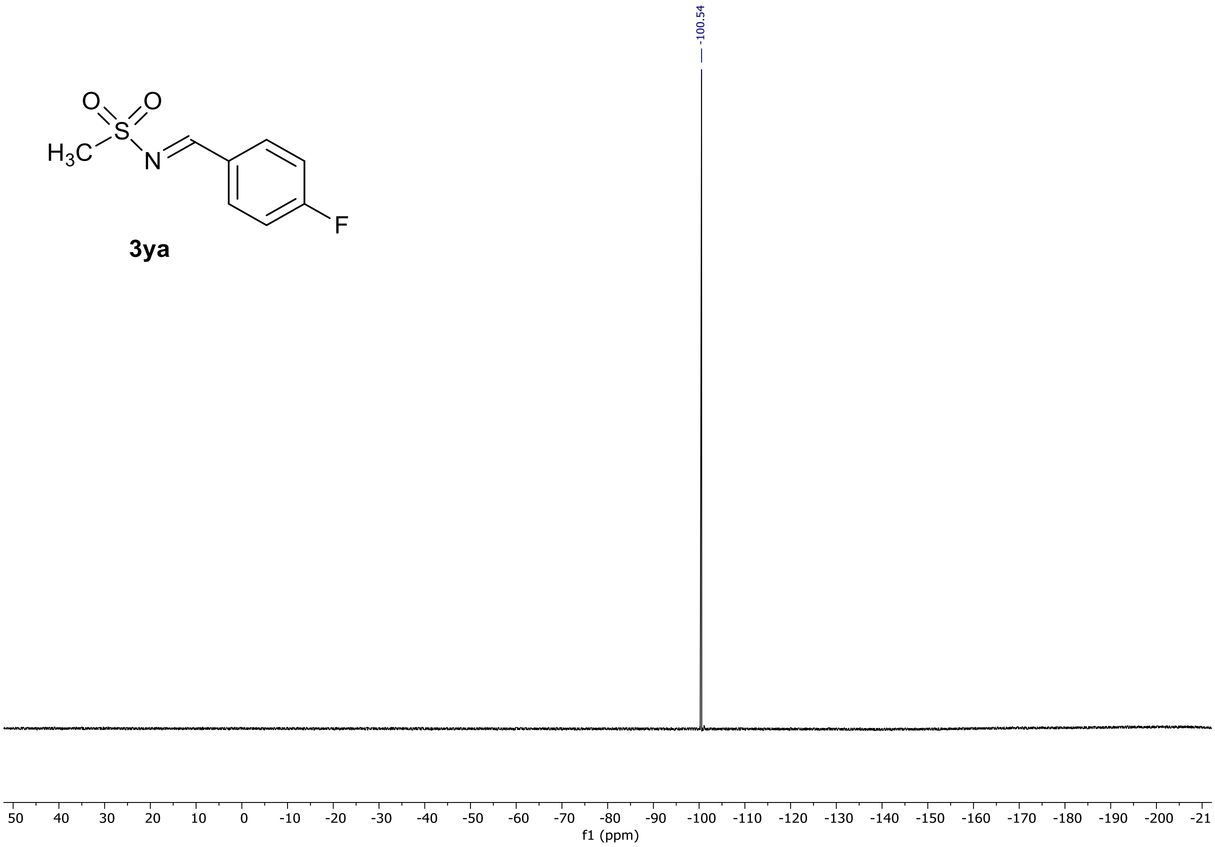
O
O
S
H3C
N
F
-100.54
50 40 30 20 10 0 -10 -20 -30 -40 -50 -60 -70 -80 -90 -100 -110 -120 -130 -140 -150 -160 -170 -180 -190 -200 -21
f1 (ppm)

3ya

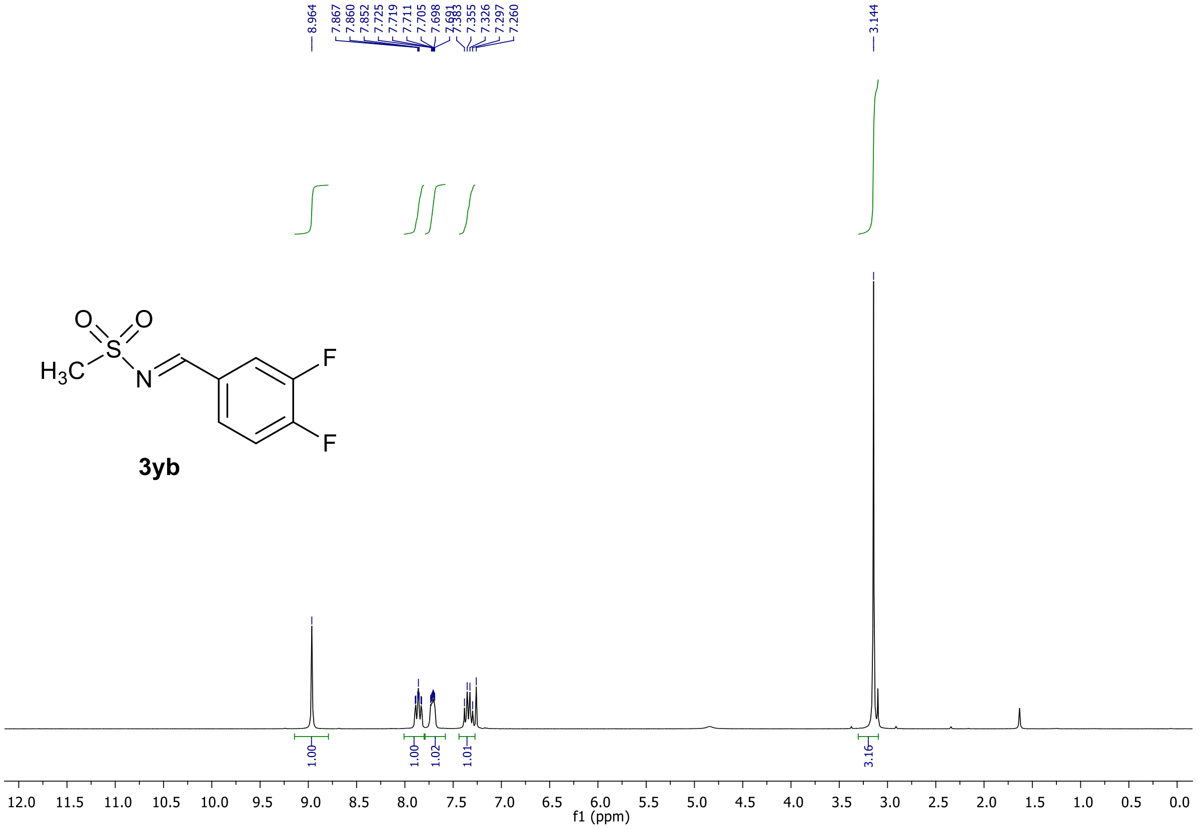
O
O
S
H3C
N
F
F
8.964
7.867
7.860
7.852
7.725
7.719
7.711
7.705
7.698
7.355
7.326
7.297
7.260
3.144
1.00
1.00
1.02
1.01
3.16
12.0 11.5 11.0 10.5 10.0 9.5 9.0 8.5 8.0 7.5 7.0 6.5 6.0 5.5 5.0 4.5 4.0 3.5 3.0 2.5 2.0 1.5 1.0 0.5 0.0
f1 (ppm)

3yb

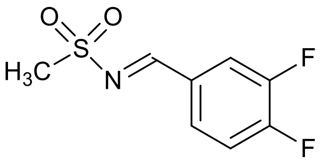
O
O
S
H_3C
N
F
F

3yb

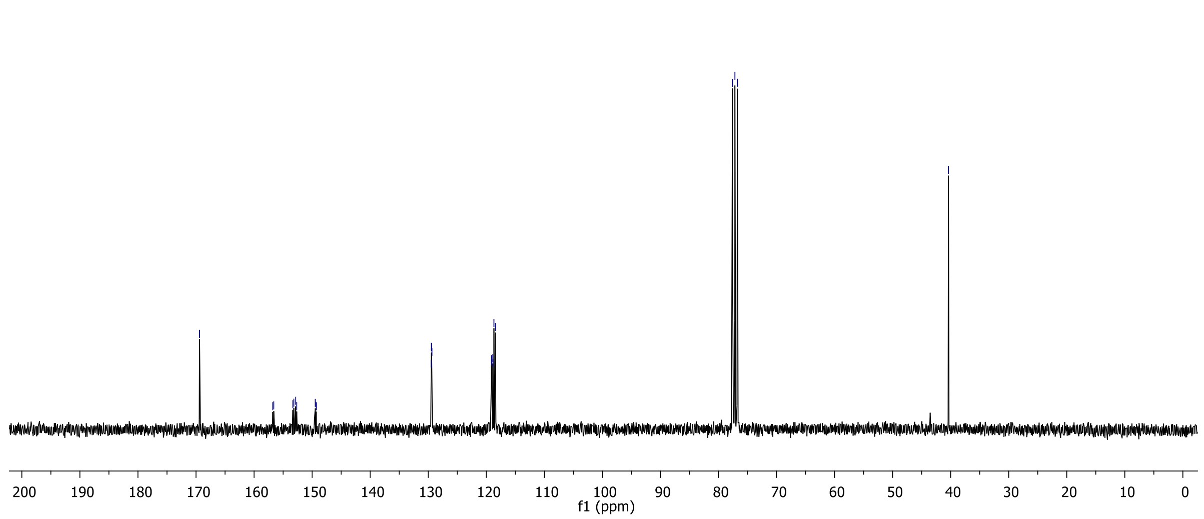
169.38
169.37
156.77
156.59
153.31
153.14
152.81
152.64
149.47
149.29
129.50
129.46
129.40
129.35
119.10
119.08
118.86
118.83
118.67
118.43
77.58
77.16
76.74
40.38
200 190 180 170 160 150 140 130 120 110 100 90 80 70 60 50 40 30 20 10 0
f1 (ppm)

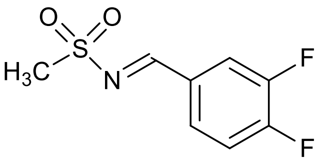
O
O
S
H_3C
N
F
F

3yb

3z

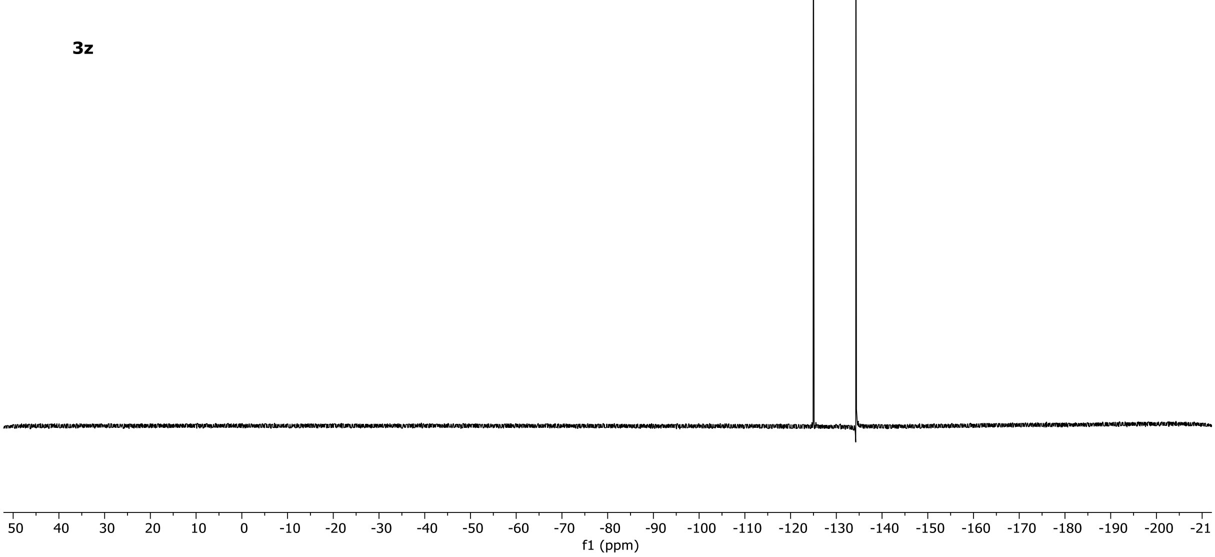
-124.98
-125.05
-134.28
-134.35
50 40 30 20 10 0 -10 -20 -30 -40 -50 -60 -70 -80 -90 -100 -110 -120 -130 -140 -150 -160 -170 -180 -190 -200 -21
f1 (ppm)

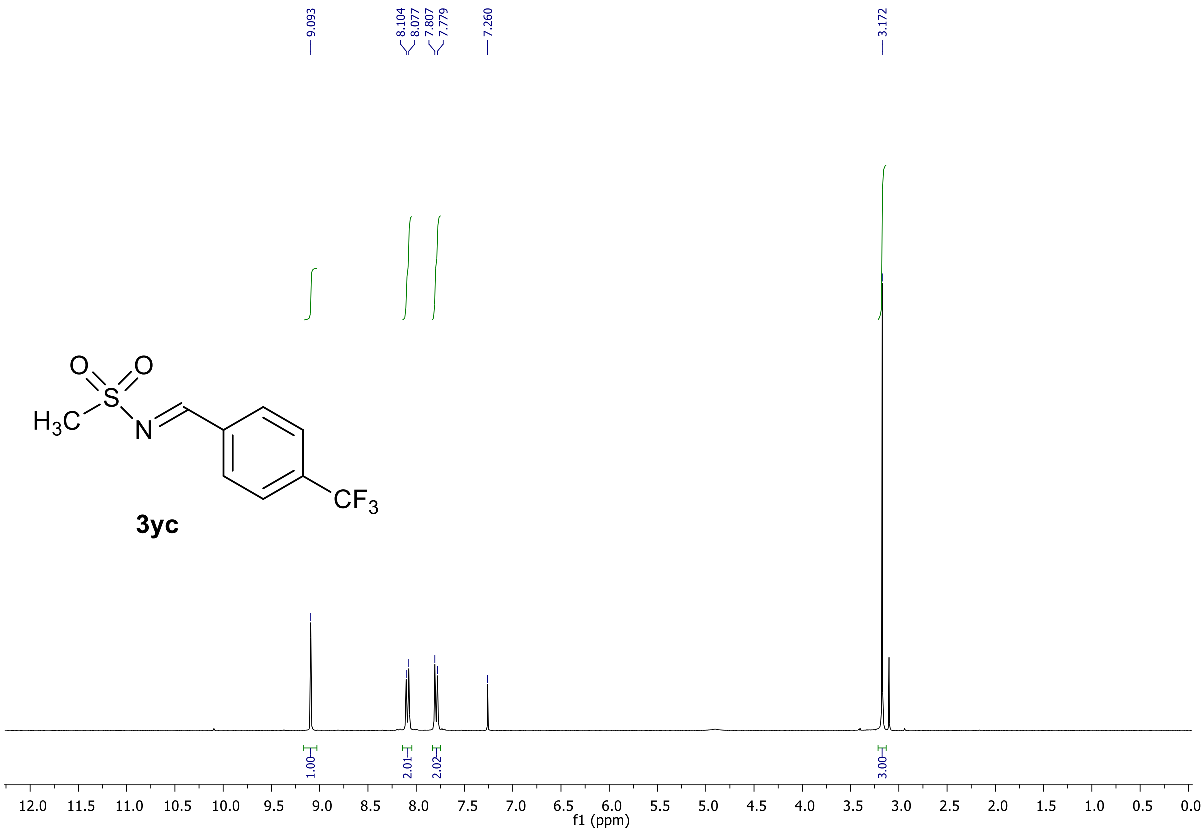
9.093
8.104
8.077
7.807
7.779
7.260
3.172
1.00
2.01
2.02
3.00
f1 (ppm)

3yc

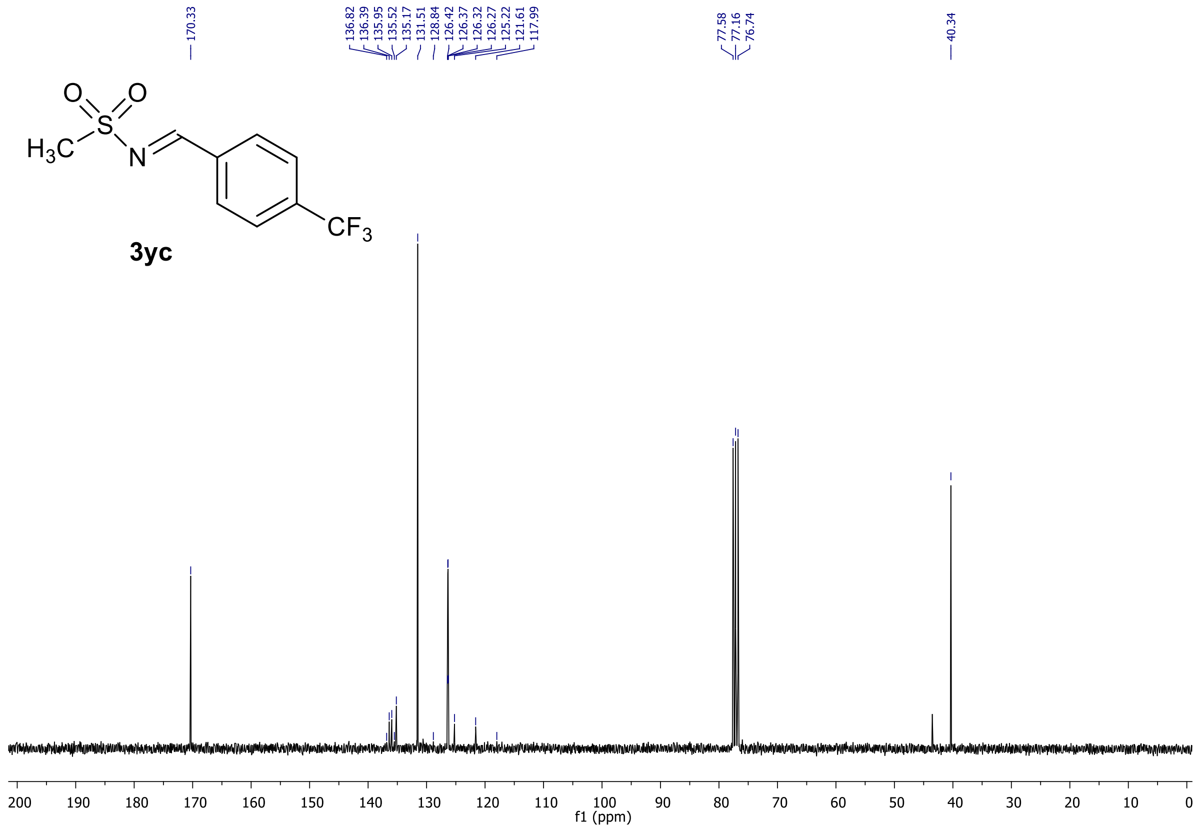
170.33
136.82
136.39
135.95
135.52
135.17
131.51
128.84
126.42
126.37
126.32
126.27
125.22
121.61
117.99
77.58
77.16
76.74
40.34
f1 (ppm)

3yc

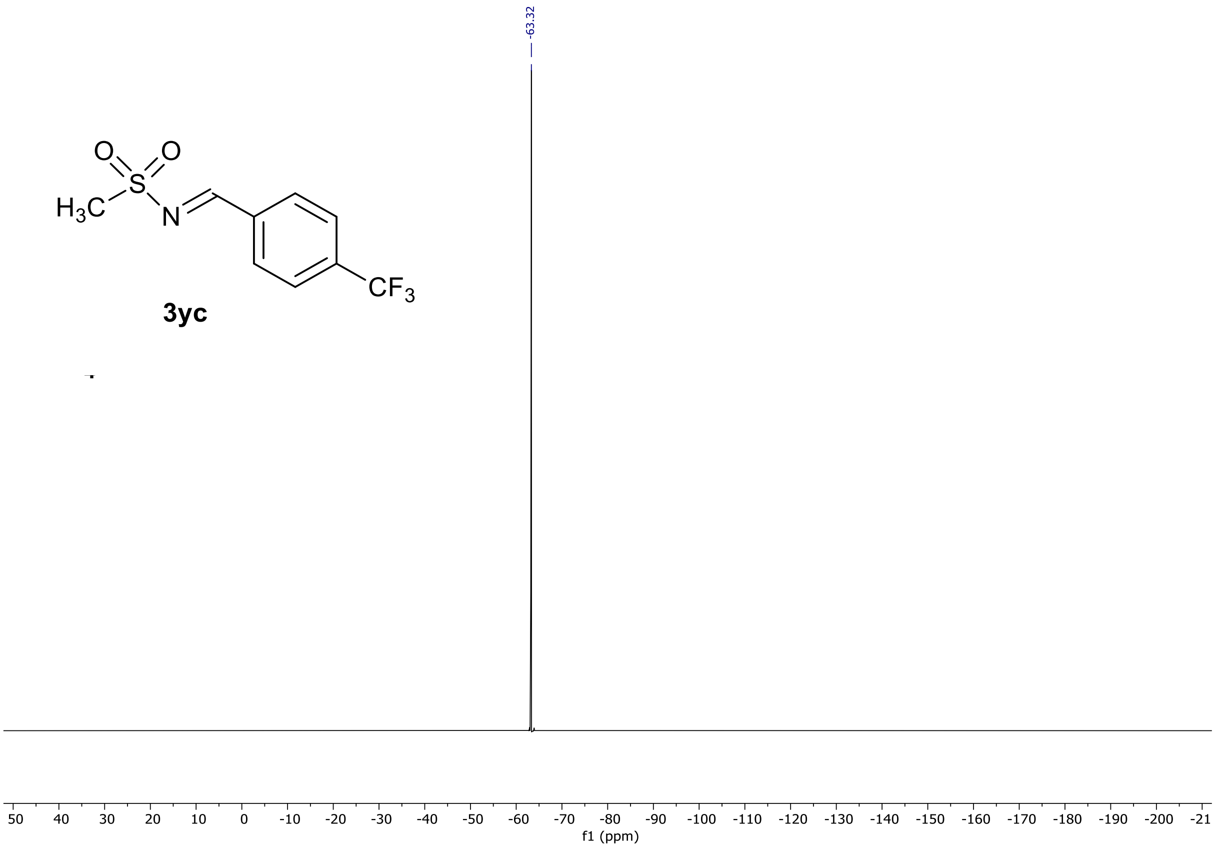
H3C
S
O
O
N
CF3
3yc
-63.32
50 40 30 20 10 0 -10 -20 -30 -40 -50 -60 -70 -80 -90 -100 -110 -120 -130 -140 -150 -160 -170 -180 -190 -200 -21
f1 (ppm)
